# Sequence- and structure-specific RNA oligonucleotide binding attenuates heterogeneous nuclear ribonucleoprotein A1 dysfunction

**DOI:** 10.3389/fmolb.2023.1178439

**Published:** 2023-06-22

**Authors:** Joseph P. Clarke, Patricia A. Thibault, Sakina Fatima, Hannah E. Salapa, Subha Kalyaanamoorthy, Aravindhan Ganesan, Michael C. Levin

**Affiliations:** ^1^ Department of Health Sciences, College of Medicine, University of Saskatchewan, Saskatoon, SK, Canada; ^2^ Office of the Saskatchewan Multiple Sclerosis Clinical Research Chair, University of Saskatchewan, Saskatoon, SK, Canada; ^3^ Department of Medicine, Neurology Division, University of Saskatchewan, Saskatoon, SK, Canada; ^4^ ArGan’s Lab, School of Pharmacy, Faculty of Science, University of Waterloo, Waterloo, ON, Canada; ^5^ Department of Chemistry, Faculty of Science, University of Waterloo, Waterloo, ON, Canada; ^6^ Department of Anatomy, Physiology and Pharmacology, University of Saskatchewan, Saskatoon, SK, Canada

**Keywords:** hnRNPA1, RNA oligonucleotide, optogenetics, stress granules, RNA-protein interaction

## Abstract

The RNA binding protein heterogeneous nuclear ribonucleoprotein A1 (A1) regulates RNA metabolism, which is crucial to maintaining cellular homeostasis. A1 dysfunction mechanistically contributes to reduced cell viability and loss, but molecular mechanisms of how A1 dysfunction affects cell viability and loss, and methodologies to attenuate its dysfunction, are lacking. Utilizing *in silico* molecular modeling and an *in vitro* optogenetic system, this study examined the consequences of RNA oligonucleotide (RNAO) treatment on attenuating A1 dysfunction and its downstream cellular effects. *In silico* and thermal shift experiments revealed that binding of RNAOs to the RNA Recognition Motif 1 of A1 is stabilized by sequence- and structure-specific RNAO-A1 interactions. Using optogenetics to model A1 cellular dysfunction, we show that sequence- and structure-specific RNAOs significantly attenuated abnormal cytoplasmic A1 self-association kinetics and A1 cytoplasmic clustering. Downstream of A1 dysfunction, we demonstrate that A1 clustering affects the formation of stress granules, activates cell stress, and inhibits protein translation. With RNAO treatment, we show that stress granule formation is attenuated, cell stress is inhibited, and protein translation is restored. This study provides evidence that sequence- and structure-specific RNAO treatment attenuates A1 dysfunction and its downstream effects, thus allowing for the development of A1-specific therapies that attenuate A1 dysfunction and restore cellular homeostasis.

## Introduction

RNA binding proteins (RBP) support cellular function by controlling several essential facets of RNA metabolism. The RBP heterogeneous nuclear ribonucleoprotein A1 (hnRNPA1 or A1), a member of the A/B subfamily of hnRNPs, performs vital roles in gene expression through transcriptional regulation, RNA splicing and RNA nucleocytoplasmic transport to ribosomal targets (Clarke et al., 2021a). These functions define A1 as an important regulator in controlling and maintaining normal cellular activities.

Under homeostatic conditions, A1 is predominantly nuclear where it maintains normal RNA metabolic functions (Clarke et al., 2021a). Pathologically, studies have shown that A1 dysfunction is characterized by cytoplasmic mislocalization of A1, altered A1 self-interaction dynamics and clustering, which affects RNA metabolism, protein translation and the formation of cytoplasmic stress granules (SGs) (Lee and Levin, 2014; Douglas et al., 2016; Salapa et al., 2018; Salapa et al., 2020a; Salapa et al., 2020b; Libner et al., 2020; Masaki et al., 2020; Anees et al., 2021; Clarke et al., 2021b). These studies are supported by reports demonstrating that the C-terminal prion-like domain (PrLD) of A1 specifically functions to mediate homo- and heteromeric protein-protein interactions and affects the liquid-liquid phase separation (LLPS) of A1. Further, mutations that dysregulate these interactions or alter A1 LLPS, lead to alterations of downstream functions, such as RNA metabolism (Ding et al., 1999; Rebane et al., 2004; Allemand et al., 2005; He and Smith, 2009; Kim et al., 2013; Molliex et al., 2015; Clarke et al., 2021a; Martin et al., 2021). Overall, A1 dysfunction has led to two proposed mechanisms of altered cellular function, whereby A1 loss-of-function in the nucleus or gain of toxicity in the cytoplasm promotes cellular pathology.

**FIGURE 1:**
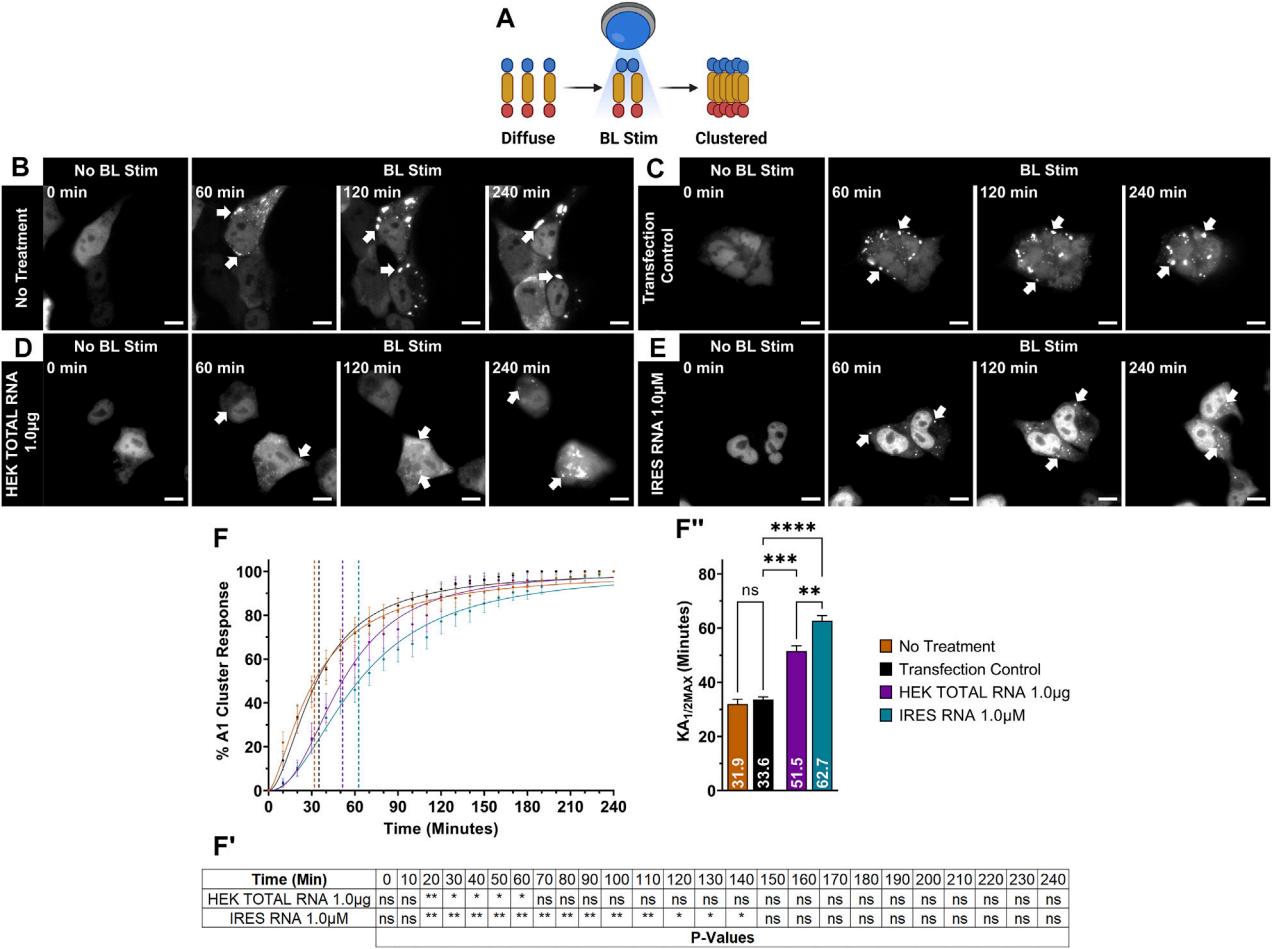
OptoA1 clustering is attenuated with the addition of non-sequence specific RNAs. **(A)** Schematic of BL-inducible A1 self-association clustering using the Cry2PHR optogene (blue), A1 protein (yellow) and mCherry fluorescent tag (red). Representative images of OptoA1 blue light (BL) stimulated cells treated with either **(B)** No RNA treatment, **(C)** RNA transfection control, **(D)** 1.0 µg HEK Total RNA or **(E)** 1.0 µM IRES RNA. **(F)** Quantification of A1 cluster formation during a 240-min BL stimulation protocol with the addition of either total RNA collected from HEK293T cells (HEK Total RNA 1.0 ug; Purple) or HIV-1 IRES (IRES) RNA 1.0 µM (Turquoise). Results are plotted as a percent maximum to the highest cluster response at 240 min for HEK Total RNA or IRES RNA treatments, resulting in a kinetics curve for association dynamics. No Treatment (Brown) = no treatment with RNA; Transfection Control (Black) = cells only transfected with RNAiMAX. Dashed lines indicate KA_1/2Max_. **(F** ^**I**^ **)** Tabular results of a two-way ANOVA, with a Bonferroni post-hoc test from the curves illustrated in **(F)**. **(F** ^**II**^ **)** Bar graphs and one-way ANOVA, with a Tukey post-hoc test analysis of KA_1/2Max_ from the curves illustrated in **(F)**. Data shown are mean±S.E.M. for three biological replicates. Scale bars = 10 µm *p < 0.05; **p < 0.01; ***p < 0.001; ****p < 0.0001; 95% Confidence Interval.

To develop methods to both inhibit and reverse A1 dysfunction, there is an urgent need to understand A1 function, but experimental systems that model A1 dysfunction *in vitro* to assess its downstream cellular effects are lacking. To accomplish this, we previously established an *in vitro* optogenetic A1 self-association paradigm that controls self-association of A1 with blue light utilizing the optogenetic protein Cryptochrome 2 (Cry2PHR) (Clarke et al., 2021b). This study demonstrated that optogenetic A1 self-association recapitulates aspects of A1 dysfunction observed in cells and established a model that assesses A1 function *in vitro*. Using our optogenetic A1 self-association paradigm (Figure 1A), this study sought to test RNA Recognition Motif (RRM)- and RNA sequence- and structure-specific RNA oligonucleotides (RNAOs) that could attenuate dysregulated A1 self-association, and thus modify A1 dysfunction and its downstream pathologic effects (Clarke et al., 2021b). Utilizing this optogenetics system, data-driven structural modelling, and thermodynamic protein stability analysis, we demonstrate that exogenous administration of sequence- and structural-specific RNAOs results in attenuation of A1 self-association cluster kinetics, as well as alters the quantity and size of A1 clusters within the cytoplasm. We further demonstrate that attenuating the clustering of A1 with RNAOs affects the characteristics of SG formation. Finally, we show that A1 clustering activates the integrated stress response pathway and inhibits protein translation, while treatment with RNAOs improves both effects. Together, these experiments provide important insights into the basic biology of A1 function and showcases an A1-specific method that can be utilized to study the effects of A1 dysfunction.

**Table:** 

Reagent or Resource	Source	Identifier
Antibodies		
Mouse monoclonal anti-β-Actin (8H10D10)	New England BioLabs	Cat# 3700S
Rabbit monoclonal anti-eIF2S1 [EPR23098-50]	Abcam	Cat# ab242148
Rabbit monoclonal anti-eIF2S1 (phospho S51) [E90]	Abcam	Cat# ab32157
Mouse monoclonal anti-eIF2S1	Abcam	Cat# ab5369
Rabbit polyclonal anti-G3BP [EPR13986(B)]	Abcam	Cat# ab217225
Rabbit polyclonal anti-mCherry	Cedarlane Labs	Cat# GTX128508
Mouse monoclonal anti-Digoxigenin	Millipore Sigma	Cat# 11333062910
Mouse monoclonal anti-hnRNPA1 (4B10)	Millipore Sigma	Cat# 05-1521
Mouse monoclonal anti-Puromycin [3RH11]	Kerafast	Cat# EQ0001
Alexa Fluor 488 Donkey anti-mouse IgG (H+L)	Jackson ImmunoResearch	Cat# 715-546-151
Alexa Fluor 488 Donkey anti-rabbit IgG (H+L)	Jackson ImmunoResearch	Cat# 711-546-152
Alexa Fluor 647 Goat anti-rabbit IgG (H+L)	Jackson ImmunoResearch	Cat# 111-605-047
Goat anti-mouse IgG (H+L) HRP Conjugate	Bio-Rad	Cat# 1706516
Goat anti-rabbit IgG (H+L) HRP Conjugate	Bio-Rad	Cat# 1706515
Chemicals		
Antifoam B Emulsion	Millipore Sigma	Cat# A5757
CelLytic B Cell Lysis Reagent	Millipore Sigma	Cat# C8740
cOmplete Mini, EDTA-free Protease Inhibitor Cocktail	Millipore Sigma	Cat# 4693159001
Cytobuster Protein Extraction Reagent	Fisher Scientific	Cat# 710093MI
Dithiothreitol (DTT)	Millipore Sigma	Cat# 233155
Dulbecco’s Modified Eagle Media (DMEM)	Fisher Scientific	Cat# MT-10-013-CV
FluoroBrite DMEM Media	Fisher Scientific	Cat# A1896701
Formaldehyde, 37% by weight (with preservative/certified ACS)	Fisher Scientific	Cat# F79-500
Gibco Fetal Bovine Growth Serum	ThermoFisher Scientific	Cat# 12483020
Halt Protease Inhibitor Cocktail (100X)	Fisher Scientific	Cat# PI87786
Halt Phosphatase Inhibitor Cocktail (100X)	Fisher Scientific	Cat# PI78420
Immobilon-FL PVDF Membrane	Millipore Sigma	Cat# IPVH00010
Invitrogen ProLong Gold Antifade Mountant with DAPI	Fisher Scientific	Cat# P36935
Isopropyl-β-D-Thiogalactopyranoside (IPTG)	Fisher Scientific	Cat# BP1755
Licor Chameleon Duo Pre-Stained Protein Ladder	Cedarlane	Cat# 928-60000
Lipofectamine 2000	Fisher Scientific	Cat# 11668-019
Lipofectamine RNAiMAX	Fisher Scientific	Cat# AAA1626622
D-(+)-Maltose monohydrate, 95%	ThermoFisher Scientific	Cat# 137780-150
Opti-MEM Reduced Serum Media	Fisher Scientific	Cat# 31985062
Poly-D-Lysine Hydrobromide	Millipore Sigma	Cat# P6407
Puromycin dihydrochloride from *Streptomyces alboniger*	Millipore Sigma	Cat# 58-58-2
Sodium Arsenite Solution	Millipore Sigma	Cat# 106277
Sodium Dodecyl Sulfate (SDS)	Fisher Scientific	Cat# BP166
SYPRO Orange Protein Gel Stain	ThermoFisher Scientific	Cat# S6650
Triton X-100	Fisher Scientific	Cat# AC215682500
Tween 20	Fisher Scientific	Cat# BP337
Commercial Assays		
Amicon Ultra-2 Centrifugal Filter Unit, 3K	Millipore Sigma	Cat# UFC200324
Clarity Western ECL Substrate	Bio-Rad	Cat# 1705060
Cytiva MBPTrap HP Prepacked Columns	Fisher Scientific	Cat# 45-001-540
NEBuilder HiFi DNA Assembly Cloning Kit	New England BioLabs	Cat# E5520S
RNeasy Plus Mini Kit	Qiagen	Cat# 74136
TranscriptAid T7 High Yield Transcription Kit	ThermoFisher Scientific	Cat# K0441
Experimental Models: Cell Lines		
HEK293T	ATCC	Cat# CRL-11268
Recombinant DNA Plasmids		
pmCry2PHR-A1WT-mCherry	Clarke et al. (2021b)	N/A
pmCry2PHR-A1(R55A)-mCherry	This paper	N/A
pmVenus-NcVV-LOV_231	Addgene	Cat# 58689
pHR-H13LTat-CD8a/d2eGFP-IRES-Nef	Addgene	Cat# 126552
pmCMV-T7-HIV-1-IRES	This paper	N/A
pmMBP-hnRNPA2_FL_WT	Addgene	Cat# 98662
pmMBP	This paper	N/A
pmMBP-TEV-A1WT	This paper	N/A
pmMBP-TEV-A1(RRMs)	This paper	N/A
pmMBP-TEV-A1(PrLD)	This paper	N/A
Bacterial Cloning		
BL21 (DE3) Chemically Competent *E. coli*	Millipore Sigma	Cat# CMC0014
Terrific Broth	Millipore Sigma	Cat# 71754
Primers		
Gibson Assembly Primers (See Supplementary Table S1)	IDT	N/A
RNA Oligonucleotides		
MAXIMUM (See Gibson Assembly Primers (See Supplementary Table S1)	IDT/Jain et al. (2017)	N/A
MEDIUM (See Gibson Assembly Primers (See Supplementary Table S1)	IDT/Jain et al. (2017)	N/A
LOW (See Gibson Assembly Primers (See Supplementary Table S1)	IDT/Jain et al. (2017)	N/A
Software		
Adobe Photoshop 2022	Adobe	https://www.adobe.com
GraphPad Prism 9.1.2	GraphPad Software Inc.	https://www.graphpad.com
Fiji 2.5.0	Rueden et al. (2017)	https//imagej.net/software/fiji
ZEN 3.1 Blue Edition	Carl Zeiss Microscopy, LLC	https://www.zeiss.com

Key Resources Table Listed are the reagents/resources utilized in this study. Their purchase source and catalogue identifiers are included.

## Materials and methods

### Cell culture

Human embryonic kidney 293T (HEK293T) cells (female, purchased from ATCC) were maintained in DMEM (ThermoFisher Scientific) supplemented with 10% fetal bovine serum (ThermoFisher Scientific) and 1% penicillin/streptomycin (ThermoFisher Scientific) at 37°C and 5% CO_2_. Cells were seeded onto 4-well 35 mm glass bottom imaging dishes (ibidi GmbH) or 8-well slides (Fisher Scientific) coated with poly-D-lysine (50 mg/mL) (Millipore Sigma), or 6-well plates (Fisher Scientific), and incubated for 24 h prior to transfections (Lipofectamine 2000; ThermoFisher Scientific) with 400 ng (4-well slides), 200 ng (8-well slides) or 2.5 µg (6-well plate) of DNA. Cell media was 50% exchanged 4 h post-transfection. Cell cultures were lightly covered with aluminum foil after transfection to provide protection from ambient light exposure and non-specific activation of the optogenetic protein, and all manipulations were performed in ambient light-free conditions.

### Cloning

We previously reported the isolation and cloning of the full-length A1 gene (Gene ID: 3,178; Accession #: NM_002136.4) into N-pmCry2PHR-mCherry-C (OptoA1) (Lee and Levin, 2014; Clarke et al., 2021b), which was used in this study. Additionally, for this study we mutated amino acid R55A in OptoA1 using HiFi DNA assembly cloning (NEBuilder HiFi DNA Assembly Cloning Kit, New England BioLabs) to generate N-pmCry2PHR-A1 (R55A)-mCherry-C. Briefly, we restriction digested N-pmCry2PHR-A1WT-mCherry-C with BamHI and AhdI, and PCR cloned overlapping regions from undigested N-pmCry2PHr-A1WT-mCherry-C that mutated amino acid R55 (AGG) to A55 (GCG).

N-pmCMV-T7-HIV-1-IRES-C was generated using HiFi DNA assembly cloning (NEBuilder HiFi DNA Assembly Cloning Kit, New England BioLabs). Briefly, the HIV-1 IRES sequence was PCR cloned out of N-pHR-H13LTat-CD8a/d2eGFP-IRES-Nef-C (a gift from Jonathan Karn, Addgene, Plasmid #126552) (Dobrowolski et al., 2019), and inserted into the N-pmVenus-NcVV-LOV_231-C plasmid (a gift from Harald Janovjak, Addgene, Plasmid #58689) (Grusch et al., 2014) between the NheI and BamHI restriction enzyme sites with two-fragment HiFi DNA assembly cloning. This cloning fully replaced the mVenus-NcVV-LOV_231 gene cassette with the HIV-1 IRES sequence and maintained the important upstream T7 promoter. Additionally, the HIV-1 IRES sequence did not have a start ATG site, as this plasmid was predominantly used to synthesize HIV-1 IRES RNA (see section below).

The N-MBP-TEV-A1WT-C (MBP: maltose binding protein; A1WT: wild-type A1), N-MBP-TEV-A1 (RRMs)-C (RRM: RNA Recognition Motif) and N-MBP-TEV-A1(PrLD)-C plasmids were constructed by PCR cloning A1WT, A1 (RRMs) and A1(PrLD) from the N-pmCry2PHR-A1WT-mCherry-C plasmid and cloning into the N-MBP-hnRNPA2_FL_WT-C base plasmid (a gift from Nicolas Fawzi, Addgene, Plasmid #98662) (Ryan et al., 2018) between the NdeI and XhoI restriction enzyme sites with two-fragment HiFi DNA assembly cloning. The N-MBP-C plasmid was generated by PCR cloning MBP from the N-MBP-hnRNPA2_FL_WT-C plasmid and inserted back into N-pmMBP-hnRNPA2_FL_WT-C between the XbaI and XhoI restriction enzyme sites with two-fragment HiFi DNA assembly cloning. This cloning fully replaced the MBP-hnRNPA2_FL_WT gene cassette with the MBP gene only.

The expression of the N-pmCry2PHR-A1WT-mCherry-C plasmid was driven by a CMV promoter. Restriction digest confirmation and subsequent sequencing (Nucleic Acid Solutions, National Research Council of Canada, Saskatoon, Saskatchewan, Canada) of all the plasmids was performed to confirm correct orientation, and to confirm the absence of unwanted mutations. All primer sequences used for cloning are listed in Supplementary Table S1.

### Recombinant protein production

For production and purification of MBP-tagged A1WT, A1 (RRMs) and A1(PrLD) proteins, as well as MBP alone, MBP-containing plasmids were first transformed in BL21 (DE3) *E. coli* and were grown overnight on agar plates at 37°C with kanamycin (Kan+). Multiple colonies were selected and grown overnight with shaking (300 rpm) in 5 mL Terrific Broth supplemented with 5% glycerol (TBG) and Kan+. Cultures were harvested and re-suspended in 1.0 mL of TGB with Kan+. 100 µL of resuspension was added to 50 mL of TGB with Kan+ and incubated at 37°C with shaking (300 rpm) for ∼2–3 h until a spectrophotometer OD_600_ reading of 0.3–0.4 was obtained. Cultures were then harvested and resuspended in 5.0 mL of TGB with Kan+, and then added to 250 mL of TGB with Kan+. Antifoam B emulsion was also added (0.008%, v/v) to prevent foam formation. Cultures were incubated at 37°C with shaking (300 rpm) for ∼ 4–5 h until a spectrophotometer OD_600_ reading of ∼1.0 was obtained. A new dose of Kan+ was then added, along with Isopropyl-β-D-Thiogalactopyranoside (IPTG) at a final concentration of 1.0 mM. Cultures were incubated at 37°C with shaking (300 rpm) for 4 h. Protein isolation was then performed by harvesting the final culture and lysing the cells with lysis buffer [700 µL CelLytic B, one cOmplete Mini, EDTA-free protease inhibitor cocktail tablet, 1.0 mM Dithiothreitol (DTT) and 10% Glycerol in 0.01M Phosphate Buffer Saline (PBS), pH 7.4]. Cell lysates were then freeze/thawed (−80°C, 5 min/37°C, 2 min) three times before centrifugation at 18,000 x g for 15 min at 4°C. Supernatants were then run through Cytiva MBPTrap HP prepacked columns, according to the manufacturer’s instructions. Columns were then washed with binding buffer (200 mM NaCl, 1.0 mM EDTA, 1.0 mM DTT, 10% Glycerol in 20 mM Tris-HCl, pH 7.4) and protein was eluted with elution buffer [200 mM NaCl, 1.0 mM EDTA, 1.0 mM DTT, 10% Glycerol, 10 mM D-(+)-Maltose in 20 mM Tris-HCl, pH 7.4]. Collected protein eluates were then concentrated and buffer exchanged (protein buffer: 150 mM NaCl in 10 mM Sodium Phosphate Buffer, pH 6.8) using Amicon Ultra-2 3 kDa centrifugal filter units, according to the manufacturer’s instructions. These were then snap frozen in liquid nitrogen and stored at −80°C until use.

### Total HEK293T RNA extraction and HIV-1 IRES RNA production

Total HEK293T RNA was extracted utilizing the RNeasy Plus Mini Kit (Qiagen) according to the manufacturer’s instructions, and its concentration was determined using Nucleic Acid, RNA-40 on a NanoDrop 1,000 Spectrophotometer (ThermoFisher Scientific). IRES RNA was synthesized from our designed plasmid (outlined above) utilizing the TranscriptAid T7 High Yield Transcription Kit (ThermoFisher Scientific) according to the manufacturer’s instructions, and its concentration was determined using Nucleic Acid, RNA-40 on a NanoDrop 1,000 Spectrophotometer.

### RNA and oligonucleotide treatments

Based upon previously published literature, A1-specific RNA oligonucleotides (RNAOs) were synthesized with a 2′OMe modification using Integrated DNA Technologies (IDT) (See Supplementary Table S1) (Jain et al., 2017). For treatments, HEK Total RNA (1.0 µg, 500 ng or 250 ng), IRES RNA [Stock = ∼36 µM (4.392 μg/μL); Working solution (calculated based upon the total volume of cell media) = 2.0 µM, 1.0 µM or 500 µM] or RNAOs [Stock = 100 µM (0.88 μg/μL); Working solution (calculated based upon total volume of cell media) = 2.0 µM, 1.0 µM or 500 µM] were transfected into HEK293T cells using Lipofectamine RNAiMAX reagent (ThermoFisher Scientific), according to the manufacturer’s instructions. Cells were transfected for 30 min prior to further experimentation. RNA transfection amounts (µg) for 8-well experiments (200 µL final volume): HEK Total RNA = 1.0 µg, 500 ng, 250 ng; IRES RNA = 2.0 µM (11.12 µg), 1.0 µM (5.56 µg), 0.5 µM (2.78 µg); RNAOs = 2.0 µM (3.52 µg), 1.0 µM (1.76 µg), 0.5 µM (0.88 µg). For 6-well plate experiments (2.0 mL final volume): HEK Total RNA = 1.0µg, 500ng, 250ng; IRES RNA = 2.0 µM (111.12 µg), 1.0 µM (55.6 µg), 0.5 µM (27.8 µg); RNAOs = 2.0 µM (35.2 µg), 1.0 µM (17.6 µg), 0.5 µM (8.8 µg).

To assess the transfection of RNAOs into HEK293T cells, 2′OMe modified MAX RNAO was tagged with digoxigenin (DIG) and transfected using Lipofectamine RNAiMAX reagent (ThermoFisher Scientific), according to the manufacturer’s instructions. As outlined above, cells were transfected for 30 min prior to further experimentation. Cells were then subsequently prepared for immunocytochemistry (see below), using mouse monoclonal anti-Digoxigenin to detect cellular MAX RNAO DIG (Key Resources Table). RNAO DIG transfection amount (µg) for an 8-well experiment (200 µL final volume) = 1.0 µM (1.76 µg).

### Blue light (BL) treatments

BL stimulation was performed in poly-D-lysine (Millipore-Sigma) coated 4-well 35 mm glass bottom imaging dishes (live-cell imaging), 8-well slides (fixed-cell imaging), or 6-well plates using custom-built LED arrays designed to fit multiple cell culture vessel dimensions and withstand common temperature/humidity requirements of cell culture incubators (Clarke et al., 2021b). LED arrays were positioned 5.0 cm above the culture surface, and 10,000 lux (measurement of luminous energy per unit of time, per unit area) of 465 nm BL was used to stimulate cultured cells.

### Stress treatments

Sodium arsenite (NaAsO_2_, 0.5 mM, 30 min) treatment was used as a positive control for cell stress where indicated.

### SDS-PAGE/western immunoblotting

Soluble protein extraction from HEK293T cells was performed using Cytobuster Protein Extraction Reagent (Fisher Scientific) according to the manufacturer’s instructions. Protein extraction solutions were supplemented with 1X Halt Protease Inhibitor (Fisher Scientific) and 1X Halt Phosphatase Inhibitor (Fisher Scientific) Cocktails. Protein concentrations were determined using A280 on a NanoDrop 1,000 Spectrophotometer and subsequently analyzed by Western immunoblotting. Equivalent amounts of protein (40 µg) were separated by 10% SDS-PAGE and transferred to PVDF membranes (Millipore Sigma) using the Trans-Blot Semi-Dry Electrophoretic Transfer Cell System (Bio-Rad); Ponceau S staining was used to assess the equivalency of protein loading after SDS-PAGE transfer. Membranes were washed with 1.0M Tris-buffered saline (TBS), pH 7.4, blocked with 5% Bovine Serum Albumin (BSA) in TBS for 1 hour at room temperature, and then incubated with primary antibodies (Key Resources Table) in 5% BSA in TBS-T (0.1% Tween 20) overnight at 4°C. Following TBS-T washes, membranes were incubated with peroxidase-conjugated secondary antibodies in TBS-T (Key Resources Table) and protein bands were detected using Clarity Western ECL Substrate (Bio-Rad) and visualized using the ChemiDoc Imaging System (Bio-Rad).

### Immunocytochemistry

Cells were prepared for immunocytochemistry (ICC) by initial fixation using 4.0% formaldehyde (prepared from a 37% stock; Millipore Sigma) in 1.0M phosphate-buffer saline (PBS), pH 7.4, for 15 min at room temperature. After fixation, and three washes with PBS, cells were permeabilized with 0.3% Triton X-100 (Millipore Sigma)/PBS for 30 min. Cells were washed three times with PBS and then blocked with Sea Block Blocking Buffer (ThermoFisher Scientific) for 1 hour at room temperature. Primary antibodies (Key Resources Table) were diluted in 0.1% Triton X‐100/PBS with 10% Sea Block and incubated overnight at 4°C. Cells were then washed three times with PBS before incubation with secondary antibodies diluted in 0.1% Triton X‐100/PBS with 5% Sea Block (Key Resources Table) for 1 hour at room temperature. Cells were then washed three times with PBS and mounted in ProLong Gold Antifade Mountant with DAPI (Fisher Scientific). All images and quantification are representative of three biological replicates. 40X random fields of view (Plan Apochromat ×40 Oil objective, with a 1.40 numerical aperture; 5 per experiment) were acquired for quantification, and 50 cells were analyzed per experiment. All images were acquired using identical settings within each experiment.

### Cellular translation imaging

Cells were BL stimulated for 240 min and treated with Puromycin (1.0 μg/mL) during the last 30 min of stimulation. Cells were then prepared for ICC, with anti-Puromycin (3RH11) (Key Resources Table) used to detect Puromycin incorporation. All images and quantification are representative of three biological replicates. 40X random fields of view (Plan Apochromat ×40 Oil objective, with a 1.40 numerical aperture; 5 per experiment) were acquired for quantification, and 50 cells were analyzed per experiment. All images were acquired using identical settings within each experiment.

### Live-cell imaging

Live-cell imaging was performed using an Axio Observer 7, inverted fluorescent microscope (Carl Zeiss Microscopy, LLC), mounted with a P Lab-Tek^TM^ S1 heating insert (Carl Zeiss Microscopy, LLC) that was regulated by both a CO_2_ Module S1 and a TempModule S1 (Carl Zeiss Microscopy, LLC). Following DNA transfections and prior to RNA treatments, medium was changed to FluoroBrite phenol red-free DMEM (ThermoFisher Scientific), supplemented with 10% fetal bovine growth serum and 1% penicillin/streptomycin. After RNA transfections, cells were equilibrated in the preheated heating insert (37°C and 5% CO_2_) for 30 min prior to imaging. BL stimulation was performed using a custom-built blue light LED array that was positioned above the heating insert to stimulate cells in the live-cell incubation unit (5.0 cm above the culture surface). Cells were stimulated with BL (465 nm, 10,000 lux) for a period of 240 min to study A1 cluster formation dynamics. Live-cell images were captured using a Plan Apochromat ×40 Oil objective, with a 1.40 numerical aperture, in 5-min intervals over the total 240 min. During the 240-min stimulation period, the BL LED was turned off for 30 s for imaging every 300 s, and the Zeiss ZEN 3.1 Blue microscope software captured mCherry signal in the 594 nm channel. This was performed by synchronizing the LED and microscope using a Nearpow Multifunctional Infinite Cycle Programmable Plug-in Digital Timer Switch (Nearpow) attached to and controlling the LED array, and the Experimental Designer Module (Carl Zeiss Microscopy, LLC) in ZEN 3.1 Blue Edition (Carl Zeiss Microscopy, LLC) controlling the 594 nm stimulation and capture. Data presented are representative of three biological replicates, utilizing 10 random fields of view per experiment. Overall, 100 cells were analyzed per biological replicate.

### Differential scanning fluorimetry

Differential Scanning Fluorimetry (DSF) was performed using the Quant Studio 3 Real-Time PCR System from Life Technologies, following the SYBR green Melt Curve protocol to obtain a T_m_ value. Briefly, in a single well of a 96-well PCR plate, a 20 µL reaction solution was placed that contained 10 µg of either MBP alone, MBP-A1WT, MBP-RRMs or MBP-PrLD, an RNAO at an experimental concentration, 10X SYPRO Orange, and a 10 mM sodium phosphate buffer containing 150 mM NaCl (pH 6.8). The melt curve program in Quant Studio 3 was set to SYBR green and run with a temperature scan from 25°C to 99°C, with the ramp temperature set to 0.05°C/s. Upon completion, the thermal unfolding curve data was displayed as first derivatives of fluorescence/temperature (dF/dT) by the Quant Studio 3 software.

### Computational methods

An array of computational methods including RNA modelling, molecular docking, molecular dynamics (MD) simulation, binding affinity calculations, and energy decomposition analysis was employed for building the three-dimensional (3D) structures of RNA-A1 RRM complexes and characterizing their interactions at the molecular level.

#### (i) RNA modeling

The secondary structural information of the RNA was predicted using the RNAFold program available within the Vienna RNA package (Gruber et al., 2008). We employed both the minimum free energy (MFE) and partition function (PF) algorithms for predicting the optimal secondary structures of RNAOs with minimum free energies that are calculated using dynamic programming (Zuker and Stiegler, 1981). A recent study (Sato et al., 2021) compared the performance of different RNA prediction programs and noted that RNAFold was able to calculate and accurately predict the secondary structures of RNA and that its folding scores were in good agreement with observed free energies. The secondary structural information of RNAOs (MAX, MED, and LOW RNAs) were used to model their 3D structures using RNAComposer program (Popenda et al., 2012) that functions based on a fragment assembly approach. In RNAComposer, the input secondary structural information is broken into multiple fragments with overlapping native base pairs, which are then matched with the known 3D structural fragments available within RNA FRABASE database (Popenda et al., 2007). The identified fragments are then assembled using the overlapping pairs to build the complete RNA 3D models. The predicted 3D structural models of RNAOs were used to model their interactions against the A1 protein.

#### (ii) RNA-protein docking

RNA-protein docking calculations were performed to model the structural complexes of RNAOs with the A1 RRMs. While the structures of RNAOs in this study were modelled computationally, a previously existing high resolution X-ray crystal structure of a monomeric hnRNPA1 RRM (Morgan et al., 2015) (PBD: 4YOE) was used for docking. This structure was chosen as it represented the complete RRM domain (including both RRM1 and RRM2) of hnRNPA1 and was also co-crystallized with a 3-nucleotide long RNA made up of AGU–a motif that is part of some of the oligos in this work. It should be noted that the RNAs or ssDNAs in all the reported experimental 3D structures of bound-A1 RRM complexes contain the signature 5′-UAG-3′ motif. Further, irrespective of the sequence length of RNA (or ssDNA), AG of the 5′-UAG-3′ motif was particularly involved in the interactions with the RNPs of A1 RRM1. This confirms the highly conserved and specific nature of the binding of RNAs with a 5′-UAG-3′ motif. It is expected that our RNAOs containing an AG rich apical loop will primarily bind to the well-conserved RNA binding site in A1 RRM1. Therefore, we docked our RNAOs into the known binding pocket within the A1 RRMs.

The docking calculations were carried out using the MDockPP program (Huang and Zou, 2010), which employs a 2-tier screening approach: In the first step, it involves an altered Fast Fourier Transform (FFT) algorithm to identify putative binding poses based on the shape complementarity within the target molecules; and the initial binding poses are reassessed using an ensemble docking algorithm accounting for molecular flexibility and a knowledge-based scoring function (ITScorePP). The RNAOs in this work are 27-nucleotide long sequences with only variations seen in the 7 nucleotides (from 11–17 positions), which suggests that the binding affinity differences amongst the RNAOs are plausibly driven by this short stretch of sequence. Therefore, we selected these 7 nucleotides as active residues for docking against A1. Whereas, for the protein residues, we defined PHE17, PHE59 and HIS101 as the active residues for docking as they were found to interact with RNAs and DNAs in the previously reported structures in the Protein Data Bank (PDB) (Morgan et al., 2015; Thibault et al., 2021) (e.g., PDB: 4YOE). We further specified the interactions of adenines and guanines from the apical loop (i.e., 11–17 positions) with that of the selected protein residues as optional interface residues. The binding poses from molecular docking were visually inspected to choose only the complexes that resembled the native RNAOs-A1 RRM interactions in the PDB, in which an adenine and/or a guanine engaged with the active site residues in A1 RRM1.

The docking scores and the docking poses of the RNAO-RRM complexes in Supplementary Figures S1–S3 (1: MAX-RRM; 2: MED-RRM; 3: Low-RRM). For each RNAO-RRM complex, we selected the first best scoring pose that exhibited the native contacts that are known from the available experimental 3D structures of RNA bound A1 complexes. These native contacts include the stacking of an adenine (from the apical loop) in between PHE17 and HIS101 in RRM1, and positioning of a guanine (from the apical loop) at proximity to PHE59 residue from RRM1, as described in Supplementary Figure S4. For example, as shown in Supplementary Figure S1, we obtained 16 docking poses from docking MAX RNAO with A1 RRM, among which the first 4 poses did not satisfy our filtering criteria as the RNAO in these poses bound parallel to RRM1, engaging in non-specific contacts without the involvement of the apical loop. Therefore, these poses were discarded. This is not surprising as RNAs are known to make non-specific interactions driven by electrostatic contacts between nucleotides and residues, which often lead to false-positive hits (Basu et al., 2021; Arney and Weeks, 2022). Unlike the top 4 poses, the fifth ranking pose of MAX-RRM1 complex exhibited native contacts through the AG motif in the apical loop. A close-up image of this binding pose is also shown in Supplementary Figure S4. A similar filtering strategy was employed in the selection of the best complex poses for MED and LOW RNAOs (Supplementary Figures S2, S3). The selected RNAO-RRM complexes were subjected to further MD optimization and binding free energy analyses.

#### (iii) MD simulation and binding free energy calculations of RNA-A1 complexes

The RNA-A1 complexes from docking were relaxed under physiological conditions using 200 ns long MD simulation each performed with the AMBER 20 package (Case et al., 2020) and pmemd. cuda engine (Le Grand et al., 2013). We employed a combination of FF14SB (Maier et al., 2015) and the recent RNA Amber force field developed by a Rochester team (i.e., RNA-ROC) (Aytenfisu et al., 2017) for describing the structural parameters of protein and RNA, respectively, for MD simulation. Each complex was solvated in a cubic box of explicit TIP3P water molecules with 10 Å between the solute and the edge of the box. The solvated systems were charge neutralized and brought to 150 mM concentration of NaCl. All system preparation was performed using the tleap program available within the AMBER package.

The prepared complexes were initially energy minimized in 6 stages with each stage involving 1,000 steps of steepest descent minimization and 10,000 steps of conjugate gradient minimization with a pre-defined harmonic restraint. In the initial stage, a 100 kcal/mol Å−2 restraint was applied on the solute atoms which was gradually decreased to 70 > 50>40 > 30>0 kcal/mol Å−2 in the subsequent rounds of minimization. The energy minimized systems were gradually heated to 310 K (with a 15 kcal/mol Å−2 on the solute atoms) over a duration of 100 ps and, subsequently, subjected to 5 × 0.4 ns equilibration cycles that were performed under isothermal-isobaric (NPT) conditions with periodic boundary conditions. Again, the equilibration was performed with an implied restraints on the solute atoms that gradually reduced as 15 > 10>5 > 3>2 kcal/mol Å−2 in each phase. The equilibrated complexes underwent a 10 ns long MD simulation with a low restraint of 0.5 kcal/mol Å^−2^ on the solute atoms and a 10 ns long MD simulation with a lowered restraint of 0.2 kcal/mol Å^−2^ only on the RNA atoms and a PHE17 residue that was reported to be a key player in RNA recognition in the earlier studies (Morgan et al., 2015; Thibault et al., 2021). Subsequently, another 10 ns long simulation with restraints applied only on the apical loops of the RNAOs (11–17 nucleotide positions). This multi-stage MD simulation protocol was employed to allow the A1 and RNAOs to adapt their interactions (“induced-fit” effects) by minimizing non-specific electrostatic interactions that are commonly seen in RNA-protein complexes. Finally, an unrestrained production MD simulation of the three systems were conducted for 200 ns time scale to probe their molecular interactions and to predict their binding affinities. The stability of the protein and RNAOs during simulation was assessed by computing the evolution of root mean square deviation (RMSD). MD trajectory analyses were performed using the CPPTRAJ module (Roe and Cheatham, 2013) in Amber and the VMD (Humphrey et al., 1996) and UCSF Chimera (Pettersen et al., 2004) programs.

Following the MD simulation, the last 10 ns of the 200-ns long MD trajectories of the RNA-A1 complexes were used to compute their (relative) binding free energies that were computed using the MM-GBSA method with the implicit solvent model of Onufriev and Case (igb = 2) (Onufriev et al., 2004). The snapshots were sampled at a constant interval of 100 ps from the last 10 ns of the MD trajectory for these calculations. The pairwise decomposition analyses (idecomp = 2) were performed to identify the key nucleotides and amino acids that contribute to the binding free energies of the complexes. All computations were performed using MMPBSA. py.MPI script (Miller et al., 2012) included in the AmberTools 20 (Case et al., 2020).

## Quantification and statistical analysis

### SDS-PAGE/western immunoblotting

Densitometric analyses of triplicate Western immunoblots were conducted using Fiji and a calibrated grey value scale (https://imagej.nih.gov/ij/docs/examples/calibration/, NIH, Bethesda, Maryland); ratios of eIF2S1 expression (total and phosphorylated) were compared to β-actin expression.

### Immunocytochemistry

All image visualization and cell counting of transfected, non-cluster, and cluster OptoA1 containing cells were performed using ZEN 3.1 Blue Edition (Carl Zeiss Microscopy, LLC). Fixed-cell image quantification of mCherry OptoA1 cluster and Alexa Fluor 488 nm G3BP1 puncta size and numbers were examined using Fiji, with 5 fields of view analyzed per condition, encompassing 100 cells, 400 A1 clusters and 200 G3BP1 puncta analyzed over triplicate experiments. Briefly, fixed cell images were imported into Fiji as individual 16-bit.TIFF images. These were then converted to 8-bit images and processed using the Fiji fast Fourier transform (FFT) bandpass filter (larger structures down filtered to 40 pixels; small structures filtered up to 1 pixel) to smooth out small structures for analysis. Analysis scales were set based upon the magnification of the images taken. The OptoA1 clusters and G3BP1 puncta were analyzed by thresholding individual cytoplasmic clusters and puncta and using the Analyze Particles tool in Fiji. Output data gave the area (µm^2^) of each individual cluster and puncta.

For phosphorylated eIF2S1 (p-eIF2S1) quantification, fixed cell images were imported into Fiji as individual 16-bit.TIFF images. These were then converted to 8-bit images. Analysis scales were set based upon the magnification of the images taken. Cells containing OptoA1 clusters and G3BP1 puncta were outlined, and fluorescence measurements of p-eIF2S1 were obtained. Output p-eIF2S1 fluorescence data gave the integrated density (i.e., the sum of the values of the pixels in the image or selection) of fluorescence. These measurements were then background corrected using the Correct Total Cell Fluorescence (CTCF) formula:

$$CTCF=Integrated\;Density\;–\;\left(Area\;of\;Selected\;Cell\;x\;Mean\;Fluorescence\;of\;Background\;Readings\right)$$

Puromycin incorporation quantification, measuring for puromycin fluorescence, was performed similarly as phosphorylated eIF2S1.

### Live-cell imaging

Time-lapse image sequences acquired during high-throughput LED screening were visualized using ZEN 3.1 Blue Edition (Carl Zeiss Microscopy, LLC). Analysis of cells containing A1 clusters was performed manually. Only cells that contained two distinctly observable A1 clusters at each time-point in data analyses were included in the final data analysis. The number of cells containing detectable A1 clusters were tracked over time and divided by the total number of cells within the imaging field to generate a percentage of cell with inclusions for each time-point within the imaging sequence.

Quantification of light-induced A1 cluster formation was performed manually using ZEN 3.1 Blue Edition (Carl Zeiss Microscopy, LLC), again analyzing only cells that contained two distinctly observable A1 clusters at each time-point in data analysis. For A1 cluster formation rate quantification, cells with clusters were initially counted for each time-point. Each time-point cluster value was then converted to a percentage of the total cellular cluster maximum at the height of the stimulation cycle. Mean percentages for each time-point, from three biological replicates, were then plotted over-time for each stimulation cycle.

### Differential scanning fluorimetry

Differential Scanning Fluorimetry (DSF) was performed in triplicate, with first derivative (dF/dT) thermal unfolding curve data from Quant Studio 3 software analyzed and graphed as melting curves and as melting temperature bar graphs in GraphPad Prism software (Version 9.1.2).

### Statistics

Statistical significance was calculated in GraphPad Prism software (Version 9.1.2) and resulting *p* values less than or equal to 0.05 (95% Confidence Interval) were determined to be significant. One-way ANOVAs, with Tukey post-hoc analysis, and two-way ANOVAs, with Bonferroni post-hoc analysis, were used where indicated.

To determine kinetic, half-maximal formation responses, graphs were subjected to a non-linear regression curve fit, using an (agonist) vs normalized response with a variable slope least squares fit equation. *n* values described in the text and figure legends represent number of cells per experimental group, unless otherwise indicated, across multiple independent, biological experiments. Representative images and videos were prepared using Adobe Photoshop 2022 (Adobe).

## Results

### RNA treatment attenuates A1 protein self-association kinetics

We previously established the utility of our OptoA1 optogenetics system for examining the biophysical properties of A1 self-association as a model to study A1 dysfunction (Figure 1A) (Clarke et al., 2021b). Previous reports show RBP oligomerization and aggregation are dependent upon the availability of RNA to bind to the RNA Recognition Motifs (RRMs) of RBPs, and that RNA inhibits liquid-liquid phase separation (LLPS) of PrLD containing RBPs (Kuo et al., 2009; Sun et al., 2014; Molliex et al., 2015; Maharana et al., 2018; French et al., 2019; Loganathan et al., 2019; Clarke et al., 2021b). Thus, we sought to utilize our OptoA1 optogenetic system to examine the effect of RNAOs on A1 self-association, similar to what has been reported for other RBPs (Mann et al., 2019).

First, we examined whether adding exogenous, non-sequence specific RNA could attenuate A1 self-association. As A1 is an essential and universal RBP involved in all aspects of RNA metabolism and can bind to a plethora of RNA species, we initially examined the effect of non-structure and non-sequence specific total RNA isolated from HEK293T cells (HEK TOTAL RNA). We first carried out our blue light (BL) stimulation live-cell imaging paradigm on OptoA1 transfected cells without the addition of HEK TOTAL RNA over 240 min (No Treatment; NT) and observed OptoA1 cytoplasmic clusters formed at a half-maximal kinetic association rate (KA_1/2MAX_) of 32 min (Figures 1B,F, F^II^; Supplementary Video S1). Additionally, we treated the cells with RNAiMAX alone as a Transfection Control (TC) and observed a KA_1/2MAX_ of 34 min, which was comparable to no treatment, demonstrating that any self-association kinetic effects observed with RNA treatment are specifically due to the addition of exogenous RNA and not RNAiMAX (Figures 1C,F, F^II^; Supplementary Video S2). This is the control used to compare all further treatments. Upon treatment with three separate concentrations of HEK TOTAL RNA (1.0 µg, 0.5 µg or 0.25 µg), we found that OptoA1 clusters associated significantly slower in all doses compared to TC, increasing KA_1/2MAX_ by ∼ 15.4 min on average (Figures 1D,F, F^I^, F^II^; Supplementary Figure S5; Supplementary Video S3).

As HEK TOTAL RNA also interacts with all other RBPs in the cell, we next examined OptoA1 cluster formation in the presence of a distinct RNA with a limited range of interactions, and that A1 is known to bind: the internal ribosomal entry site (IRES) of human immunodeficiency virus-1 (HIV-1) (Monette et al., 2009; Gendron et al., 2011; Vallejos et al., 2012; Rollins et al., 2014; Barrera et al., 2020). We treated cells with three concentrations (2.0 µM, 1.0 µM or 0.5 µM) of *in vitro* transcribed HIV-1 IRES RNA and found that OptoA1 clusters had a significantly higher KA_1/2MAX_, by ∼23 min on average, indicating slower cluster formation compared to TC (Figures 1E,F, F^I^, F^II^; Supplementary Figure S6; Supplementary Video S4). Cumulatively, these results show that the addition of non- and semi-specific exogenous RNA impacts OptoA1 association kinetics, and the overall response of OptoA1 clustering in cells.

### Sequence- and structure-specific RNA oligonucleotides bind to and stabilize A1

We next focused on the interaction dynamics between sequence- and structure-specific RNA and A1. Previous reports have demonstrated *in vitro* and *in silico* that the isolated RRMs of A1, referred to as UP1, interact with the HIV exon splicing silencer 3 stem loop (HIV SL3^ESS3^) in the HIV-1 genome, and a report from Jain et al. (2017) evaluated sequence combinations of the 7-nucleotide loop for sequences with varying affinity for UP1 in a series of biochemical assays (Morgan et al., 2015; Jain et al., 2017). From this report, we synthesized three 2′OMe- RNAOs that we labelled as MAX, MED, and LOW based upon their reported dissociation constants to evaluate in our OptoA1 self-association paradigm.

**FIGURE 2:**
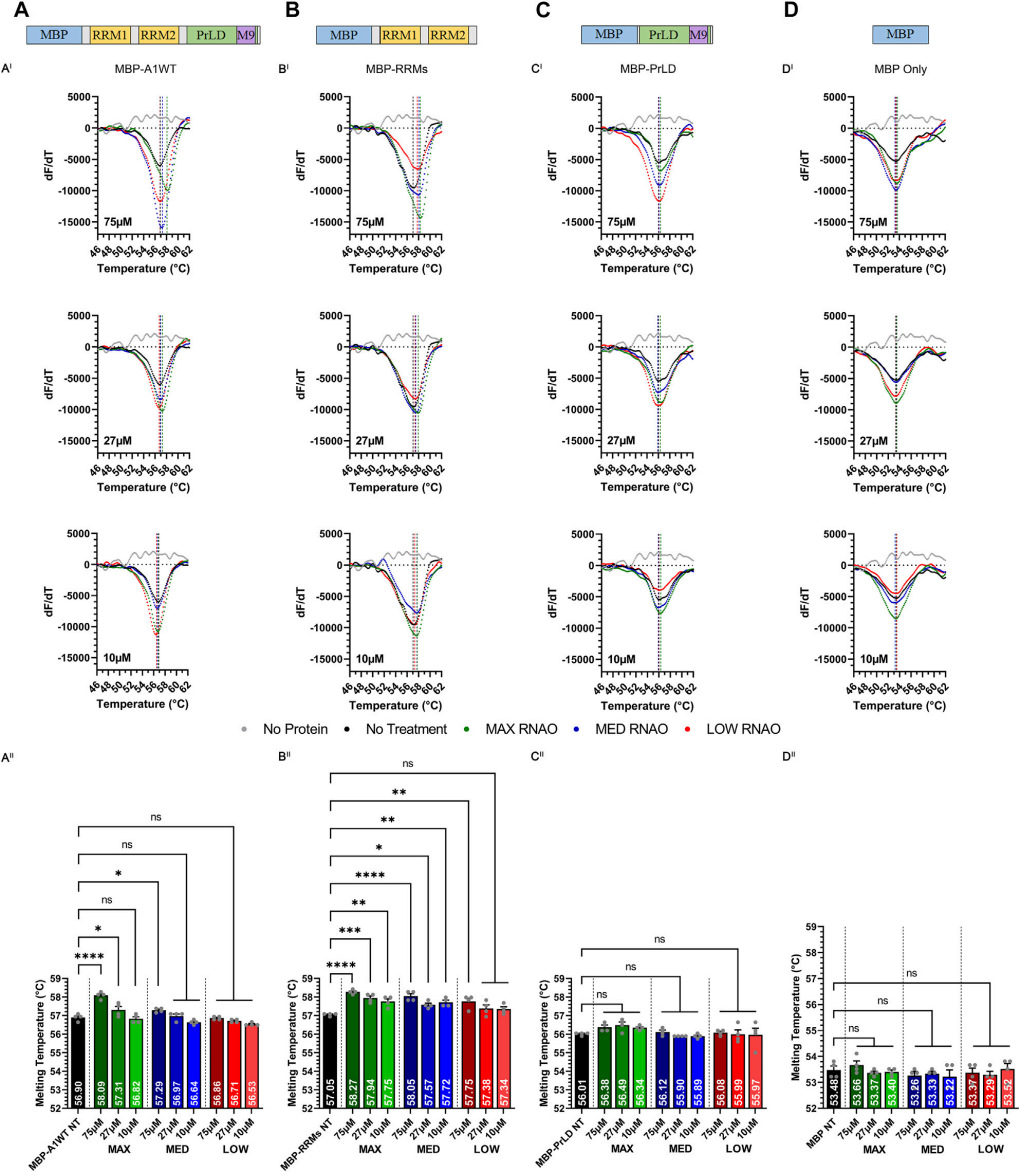
Sequence- and structure-specific RNAO binding to the RRM1 region of A1 alters the thermal stability of A1. Illustrations of **(A)** MBP-A1WT, **(B)** MBP-RRMs, **(C)** MBP-PrLD and **(D)** MBP Only. DSF melting curves of the thermal unfolding of **(A** ^**I**^ **)** MBP-A1WT, **(B** ^**I**^ **)** MBP-RRMs, **(C** ^**I**^ **)** MBP-PrLD and **(D** ^**I**^ **)** MBP Only in the presence of MAX, MED, and LOW RNAOs at different concentrations. Data shown are mean±S.E.M. for three biological replicates. Error bars are omitted for graphical clarity. Dashed lines indicate the melting temperature (T_m_). **(A** ^**II**^ **)**, **(B** ^**II**^ **)**, **(C** ^**II**^ **)** and **(D** ^**II**^ **)** Bar graphs and one-way ANOVA, with a Tukey post-hoc test analysis of Tm’s from the curves illustrated in **(A** ^**I**^ **)**, **(B** ^**I**^ **)**, **(C** ^**I**^ **)** and **(D** ^**I**^ **)**. NT = No Treatment. *p < 0.05; **p < 0.01; ***p < 0.001; ****p < 0.0001; 95% Confidence Interval.

We first evaluated whether the A1-RNA interaction could alter the structure of full-length, wild-type A1 (A1WT) in an affinity- and concentration-based manner. We conducted thermal shift assays examining differential scanning fluorimetry on recombinant A1WT tagged with maltose binding protein (MBP) to ensure its solubility (MBP-A1WT; Figure 2A), and then treated with different concentrations of RNAOs (75µM, 27 µM or 10 µM) (Figure 2A^I^). We initially found that untreated A1WT thermally destabilized (T_m_) at 56.9°C, but with the addition of MAX RNA, we observed a significant dose-dependent shift in thermal destabilization, with 75 µM shifting to a T_m_ of 58.1°C, and 27 µM shifting to 57.3°C, while 10 µM did not significantly affect stability (Figure 2A^II^). Since the T_m_ increased, we hypothesized that the binding of MAX RNA promoted A1WT to adopt a more stable conformation. A similar result was obtained with MED RNA, but only at its maximum concentration, with 75 µM shifting to 57.3°C, while treatment with LOW RNA had no effect on A1WT protein stability at any tested concentration (Figure 2A^II^). These data indicate that the predicted and modelled RNAO affinities correlate with their effect on the structure and stability of A1WT.

We next tested whether the thermal shifts in A1WT with MAX and MED RNAOs were due to binding specifically to the RRMs of A1 (MBP-RRMs) (Figure 2B), or due to off-target binding within the intrinsically disordered A1 PrLD (MBP-PrLD) (Figure 2C). Our results with MBP-RRMs demonstrate that MAX and MED RNAOs significantly affected the thermal stability of the A1 RRMs, over all concentrations (Figures 2B^I^, B^II^). Interestingly, the thermal shifts observed with MAX and MED RNA were similar, with an average thermal shift of 57.9°C observed (Figure 2B^II^), but there was still a clear dose-dependent effect. LOW RNA, however, only affected A1 RRM thermal stability at its highest dose, with 75 µM shifting to 57.8°C (Figure 2B^II^). Together, these data indicate that the RRMs alone are more responsive to RNAO binding than the full-length protein. Testing MBP-PrLD with the RNAOs, over all concentrations, resulted in no change in thermal stability, confirming that the RNAOs were only binding and altering the structure of proteins containing the A1 RRM domains (Figure 2C^I^, C^II^). Finally, we also confirmed that the MBP solubility tag did not respond to any RNAO at any concentration, indicating that our findings were due to an A1-RNA specific interaction (Figure 2D, D^I^ and D^II^). Cumulatively, these results demonstrate that binding of sequence- and structure-specific RNAOs with A1WT is RRM- and RNA sequence-dependent and alters the structure of A1WT.

To gain insights into the binding interactions between RNAOs and A1 RRMs at a molecular-level, we modelled the RNAO-bound complexes. The structural models of the complexes were initially predicted through RNA-protein docking and were optimized through 200 ns long molecular dynamics (MD) simulation to account for induced-fit effects and overall conformational dynamics from RNA binding to A1 RRM1. Assessment of root mean square deviation (RMSD) evolution during MD confirmed the overall stability of the complexes (Supplementary Figure S7). While the protein backbone and the RNAO structures in the MAX and LOW complexes reached a plateau >80 ns, the MED complex exhibited slightly higher fluctuations indicating the minor changes in the RNA-protein interactions.

Binding free energy values (ΔG_bind_) (Table 1), predicted from the last 10 ns of the equilibrated MD trajectories of the complexes, suggested that the MAX RNAO has the strongest affinity to A1 RRM1 with a ΔG_bind_ of −53.9 kcal/mol, as compared to that of the MED RNAO (−49.6 kcal/mol) and LOW RNAO (−23.5 kcal/mol). This relative ranking of the predicted binding affinity agrees with the previously reported K_d_ values (Rollins et al., 2014) and the thermal shift data from our experiments, confirming the validity of our models. These binding free energy values, and those previously reported, are based upon RNA interaction with the A1 UP1, as this domain of A1 has a known crystal structure (Sun et al., 2020). Other interactions within A1, however, may also affect its binding to RNA species, and thus affect binding free energy values.

**TABLE 1:** Comparison of the predicted binding free energies of RNAO-A1 RRM complexes against the previously reported K_d_ values and the corresponding T_m_ values from thermal shift assay (expressed as full T_m_ and delta T_m_ (ΔT_m_) compared to NT) experiments from this work.

Complex	Binding Affinity(k cal/mol)	K_d_(nm)^a^	T_m_(in°C)^b^	∆ T_m_(in°C)^b^
MAX	−53.9+/−5.3	19.4	58.3	1.22
MED	−49.6+/−5.8	27.8	58.1	1.00
LOW	−23.5+/−4.4	598	57.8	0.70

^a^ The K_d_ values are from Jain et al. (2017) and Rolins et al. (2014). These values represent the binding of only the 7-nucleotide loop from the RNAOs against A1 RRM.

^b^ Melting temperature and delta melting temperature of A1 RRMs, compared to NT, when treated with full-length RNAOs at 75 µM in this work (See Figure 2Bii for dose-dependent response).

**FIGURE 3:**
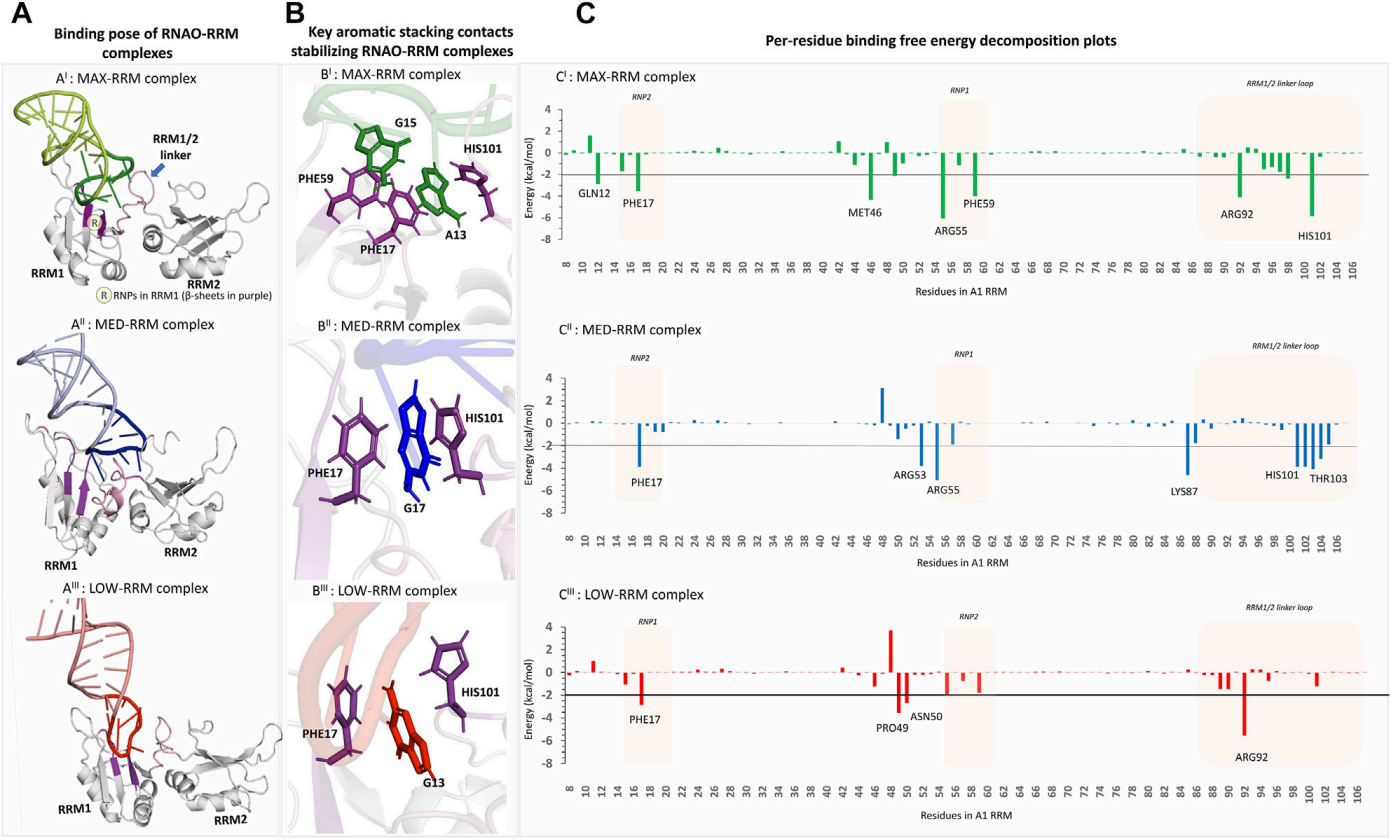
3D structural models of the RNAO-RRM complexes and the key residues contributing to their binding free energies **(A** ^**I**^ **–A** ^**III**^ **)** The structures of the RNAO-A1 RRM complexes describe that the binding of RNAOs with A1 RRMs were mediated through the interactions of the apical loops of the RNAOs with that of the RNPs from RRM1 and the RRM1/2 linker loop (coloured in purple and shown by arrow in **(A** ^**I**^ **)**. **(B)** Close-up views of the binding sites of the complexes reveal key aromatic stacking interactions between RNAOs and RRM. MAX RNAO made two aromatic interactions with PHE17-A13-HIS101 and PHE59-G15, which are consistent with the interactions reported in the known oligos-A1 RRM complexes in PDB **(B** ^**I**^ **)**. The MED **(B** ^**II**^ **)** and LOW **(B** ^**III**^ **)** RNAOs exhibit only a single aromatic stacking rendered by a guanine and PHE17. **(C)** Per-residue decomposition analyses identified other key residues in RRM1 that contributed to the binding free energies of the RNAO-RRM complexes. Several residues from RNPs and the RRM1/2 linker loop contribute to the binding free energy of the MAX complex **(C** ^**I**^ **)**. The binding free energy of MED-RRM complex is driven mostly by residues from RRM1/2 linker loop and fewer residues from RNPs **(C** ^**II**^ **)**. Apart from the stacking contact with PHE17, the binding free energy of the LOW-RRM complex is dominated by non-specific electrostatic interactions of residues not part of RNPs **(C** ^**III**^ **)**.

Binding mode analyses of the RNAO-A1 complexes (Figure 3A; Supplementary Figure S8) revealed that the RNAOs bound to the RRM1 domain of A1 and their RNA-protein interactions were driven through the 7-nucleotide apical loop (11–17 sequence positions) from RNAOs and the ribonucleoprotein motifs (RNP) (residues 15–20 and 55–60) in RRM1 and the RRM1/2 linker loop in A1. The stem segment outside the apical loop in the RNAOs, however, did not make significant contact with A1’s RRM1. The _13_AGG_15_ fragment in MAX RNAO played a central role in its recognition by the A1 RRM1, with the A13 and G15 nucleotides from MAX forming aromatic stacking interactions with upward facing PHE17 and PHE59, respectively (Figure 3B^I^; Supplementary Figure S8). These interactions can be considered as native signature contacts for nucleotide binding to A1, as confirmed by the binding poses of different DNA and RNA molecules against A1 RRMs reported in the PDB (Supplementary Figure S4) (Ding et al., 1999; Myers et al., 2003; Myers and Shamoo, 2004; Morgan et al., 2015; Thibault et al., 2021). Further, it was previously reported that specific binding of RNA to RRMs is supported by the aromatic sidechains in RNPs that adapt an upward conformation (Diarra dit Konté et al., 2017). Per-residue energy decomposition analyses revealed additional key residues that contributed (<−2 kcal/mol) to the MAX RNAO-A1 RRM1 binding free energy (Figure 3C^I^). The decomposition energies for the RNAO are provided in Supplementary Figure S9. For MAX RNAO, these include T-shaped aromatic stacking of HIS101 with A13 and salt-bridge interactions of ARG55 and GLN12 with those of the phosphate groups from G14 and the purine ring of G15, respectively (Supplemental Figure 10). Notably, arginine-phosphate group salt-bridge contacts have been suggested to play a crucial role in peptide recognition by RNA molecules (Levintov and Vashisth, 2021). Additionally, the described binding mode and the key interactions of MAX RNAO-A1 RRM were consistent with those found in the crystal structure of an “AGU” RNA in complex with A1 RRM (PDB: 4YOE). These findings together explain the superior binding and activity rendered by MAX RNAO against A1.

In MED and LOW RNAOs, since the adenine side chains are located inwards, a guanine residue in each RNA molecule (G16 and G13, respectively) engaged in the aromatic stacking contacts with PHE17 and HIS101 (Figure 3B^II−III^), which is a role consistently taken up by an adenine residue, as seen in our MAX RNAO model and the previous PDB RNA/DNA-bound A1 RRM structures. The apical loop of MED RNAO engaged mostly with the RRM1/2 linker loop formed by residues HIS101-V104, rather than the RNP motifs (refer to decomposition plot in Figure 3C^II^). For example, we observed a stable hydrogen bond between THR103 (from the linker loop) and U14 in the MED RNAO-RRM complex. Nevertheless, apart from the PHE17-G17 stacking contact, there were only few contacts between RNPs in A1 and MED RNAO, and even these contacts were highly dynamic and were seen between nucleotides outside of the apical loop in MED, such as A7 and U8 with ARG55 and ARG53 residues in A1 RRM1, respectively (Supplementary Figures S8, S11). As a result, the MED RNAO exhibited weaker affinity to the A1 RRM when compared to MAX. With respect to the LOW RNAO, although it was able to interact with A1 RRMs through G13-PHE17 aromatic contact, this complex did not display any significant native contacts, but only participated in non-specific electrostatic interactions with a small set of residues (see in decomposition plot in Figure 3C^III^; Supplementary Figure S8). Consequently, LOW RNAO had the weakest affinity to the RRMs. A comparison of the evolution of the total number of hydrogen bonds (criteria based upon Itoh et al. (2019): distance cut-off of 3.0Å; angle cut-off of 120) between RNAOs and A1 RRM complexes during MD simulation is provided in Supplemental Figure S12. This figure indicates that MAX and MED RNAOs make a total of 7-8 hydrogen bonds with the protein, whereas the LOW RNAO makes lesser interactions (average number of hydrogen bonds = 5.71).

Overall, structural insights from our models helped to explain the differential binding affinities and variable thermal shift response of the RNAOs against A1 in this study. Cumulatively, our *in vitro* and *in silico* results demonstrate that the binding of sequence- and structure-specific RNAOs with A1WT is RRM- and RNA sequence-dependent and alters the stabilization and structure of A1WT.

### RNAOs attenuate A1 protein self-association in an RNA sequence-specific and dose dependent manner

**FIGURE 4:**
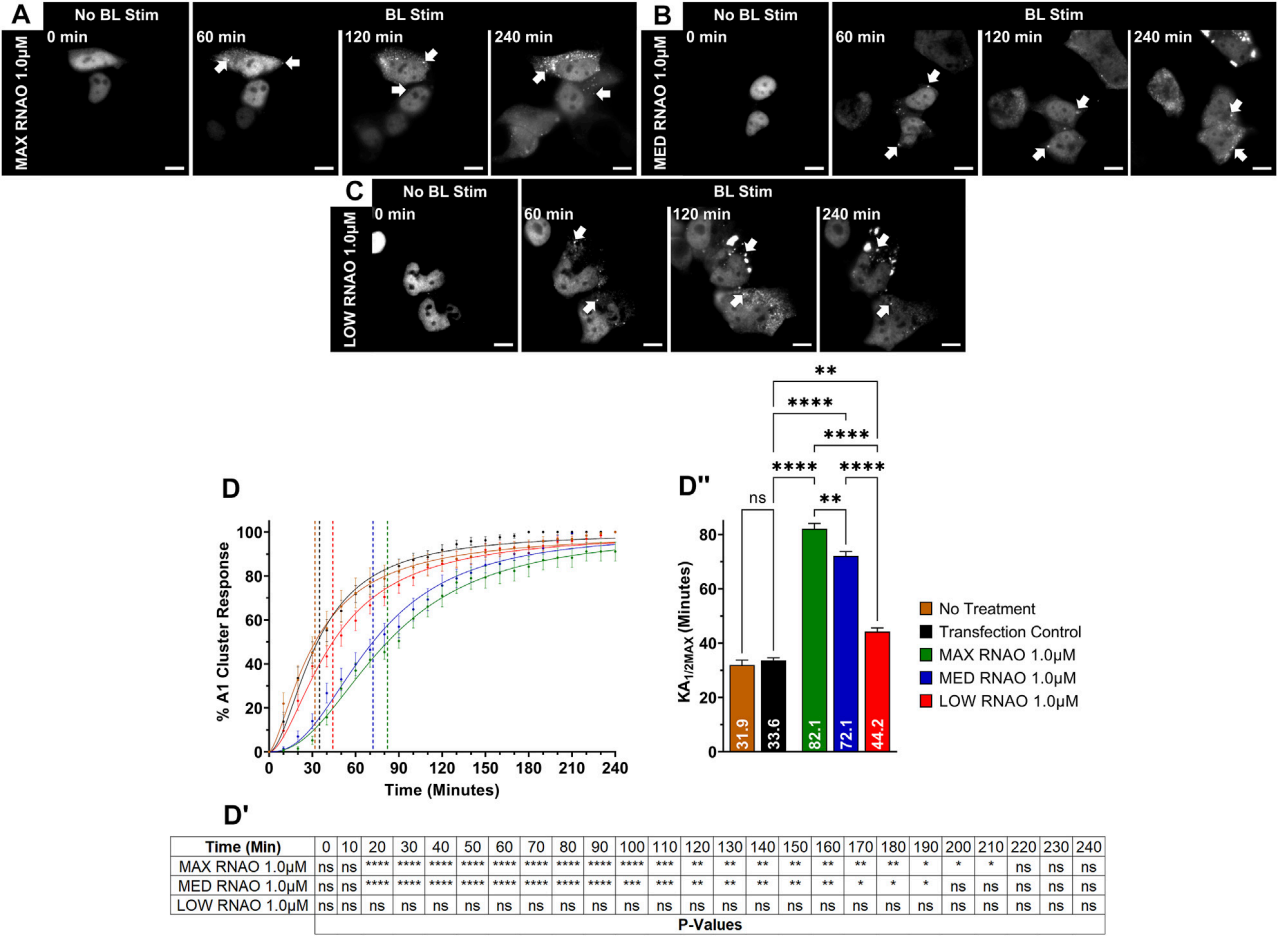
OptoA1 clustering is attenuated with the addition of sequence- and structure-specific RNAOs. Representative images of OptoA1 blue light (BL) stimulated cells treated with either **(A)** 1.0 µM MAX RNAO, **(B)** 1.0 µM MED RNAO or **(C)** 1.0 µM LOW RNAO. **(D)** Quantification of A1 cluster formation during a 240-min BL stimulation protocol with the addition of either MAX RNAO (Green), MED RNAO (Blue) or LOW RNAO (Red). Results are plotted as a percent maximum to the highest cluster response at 240 min for each RNA treatment, resulting in a kinetics curve for association dynamics. No Treatment = no treatment with RNA; Transfection Control = cells only transfected with RNAiMAX. Dashed lines indicate KA_1/2Max_. **(D** ^**I**^ **)** Tabular results of a two-way ANOVA, with a Bonferroni post-hoc test from the curves illustrated in **(D)**. **(D** ^**II**^ **)** Bar graphs and one-way ANOVA, with a Tukey post-hoc test analysis of KA_1/2Max_ from the curves illustrated in **(D)**. Data shown are mean ± S.E.M. for three biological replicates. Arrows indicate the formation of OptoA1 clusters. Scale bars = 10 µm *p < 0.05; **p < 0.01; ***p < 0.001; ****p < 0.0001; 95% Confidence Interval.

After verifying the affinities of the three 27-nucleotide RNAOs for A1 by computational methods and biochemical assays, confirming RNAO specificity for the RRM domain of A1, and demonstrating that RNAO binding in the RRMs alters the structure of the full-length protein *in vitro*, we then sought to evaluate the effect of RNAOs on OptoA1 clustering in live cells. Initially, we assessed the transfection of RNAOs into HEK293T cells by tagging MAX RNAO with digoxigenin (DIG), and subsequently performing an ICC (Supplemental Figure S13). Our results demonstrate that RNAO is ubiquitously transfected in HEK293T cells, regardless of OptoA1 transfection, and is located both in the nucleus and cytoplasm, as is expected for RNA molecules (Supplemental Figure S13). For our subsequent experimentation, we did not utilize tagged RNAOs, as were uncertain whether the DIG tag would interfere with binding to A1 and thus further complicate the interpretation of our results. With this knowledge, we experimentally tested untagged MAX RNAO, with concentrations of 2.0 µM, 1.0 µM or 0.5 µM, and found that OptoA1 clusters associated significantly slower in all concentrations compared to TC, increasing KA_1/2MAX_ by 13.3 min with 2.0 µM, 48.5 min with 1.0 µM and 25.1 min with 0.5 µM (Figures 4A,D, D^I^, D^II^; Supplementary Figure S14; Supplementary Video S5). MED RNAO treatment also showed a significantly slower OptoA1 cluster association response, increasing KA_1/2MAX_ by 38.5 min with 1.0 µM and 19.0 min with 0.5 µM, however, 2.0 µM was not significantly different from TC (KA_1/2MAX_ = 40 min) (Figures 4B,D, D^I^, D^II^; Supplementary Figure S15; Supplementary Video S6). Interestingly, the KA_1/2MAX_ result for the 2.0 µM concentration was significantly lower than both the 1.0 µM and 0.5 µM concentrations with both MAX and MED RNAOs, suggesting that there is an optimal concentration of RNAO that directly modulates OptoA1 clustering kinetics without having conflicting cell-wide effects that indirectly accelerate OptoA1 clustering (Supplementary Figure S15). Finally, while treatment with LOW RNAO showed a significantly slower OptoA1 cluster association response in all concentrations compared to TC, increasing KA_1/2MAX_ by 8.5 min with 2.0 µM, 10.6 min with 1.0 µM and 12.0 min with 0.5 µM, the magnitude of this response was much weaker than the RNAOs with a higher predicted and biochemically-confirmed affinity for A1 (Figures 4C,D, D^I^, D^II^; Supplementary Figure S16; Supplementary Video S7). Unlike MAX and MED RNAOs, the LOW RNAO did not show a dose response, as all three concentrations responded similarly (Supplementary Figure S16). When we compared the effects of RNAOs on OptoA1 clustering with previous RNA treatments (i.e., HEK TOTAL RNA and IRES RNA), we found that MAX and MED RNAOs performed significantly better (Supplementary Figure S17). LOW RNAO treatment, however, performed less significantly than IRES RNA and similarly as HEK TOTAL RNA (Supplementary Figure S17). Finally, to further demonstrate *in vitro* that the RNAOs were altering the biophysical properties of A1 self-association through a direct interaction with A1 in our optogenetic system, we mutated RRM1 [ARG55 to ALA55 (R55A)] and measured the kinetics of OptoA1 cluster formation with the addition of MAX RNAO. We chose this mutation based upon the work described in this manuscript that shows ARG55 is essential for RNAO binding in A1 RRM1 (Figure 3; Supplementary Figures S1–S4, Supplementary Figures S7–S12), as well as previous work published by Beusch et al., who showed that mutating residues in the RRMs of A1, specifically ARG55, resulted in significant RNA binding affinity loss (Beusch et al., 2017). In these experiments, we found that unlike wild-type, R55A OptoA1 did not respond to 1.0 µM MAX RNAO treatment, as there was no significant alteration in cluster formation kinetics (Supplementary Figure S18). Together, these results show that the addition of sequence- and structure-specific RNAOs impacts OptoA1 association kinetics more specifically than non-sequence and structure-specific RNA and reduces the overall response of OptoA1 clustering in cells to a greater extent.

### A1 protein cluster characteristics are altered by RNAO treatment

**FIGURE 5:**
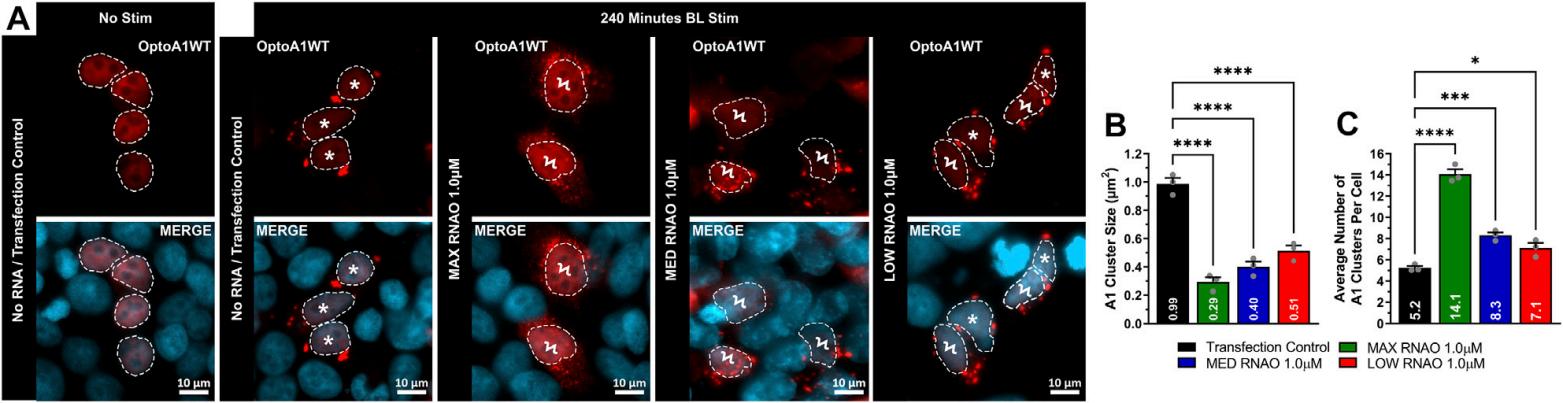
RNAOs alter the characteristics of OptoA1 clusters. **(A)** Representative images of OptoA1 blue light (BL) stimulated (240 min) cells treated with either 1.0µM MAX RNAO, 1.0 µM MED RNAO or 1.0 µM LOW RNAO. * indicate cells that contain few, large A1 clusters. Ϟ indicate cells that contain abundant, small A1 clusters. Dashed lines outline cellular nuclei. Scale bars = 10 µm. Quantification of **(B)** BL stimulated OptoA1 average cluster size and **(C)** BL stimulated OptoA1 average clusters per cell, combined from three biological experiments. All results were analyzed using a one-way ANOVA, with a Tukey post-hoc test. Data shown are mean±S.E.M. for three biological replicates. *p < 0.05; ***p < 0.001; ****p < 0.0001; 95% Confidence Interval.

We next examined whether RNAO treatment resulted in morphological changes in OptoA1 clustering in fixed-cell imaging after 240 min of BL stimulation. For these analyses, we used 1.0 µM concentrations for all RNAO treatments, as they yielded the most robust effects on OptoA1 association kinetics for all RNAOs. Analyses of OptoA1 cluster average size demonstrated that all RNAO treatments caused the formation of significantly smaller clusters, as compared to TC (Figures 5A,B). We then analyzed the average number of cytoplasmic OptoA1 clusters formed per cell during the BL induction experiment and observed significantly more clusters per cell with RNAO treatment, as compared to TC (Figures 5A,C). Interestingly, MAX RNAO treatment caused the formation of the smallest, and the most OptoA1 clusters per cell, while the MED and LOW RNAOs showed similar results (Figures 5A–C). These data indicate that RNAO treatment not only influences OptoA1 self-association dynamics, but also affects the phenotypic characteristics of OptoA1 clusters.

### RNAO treatment attenuates A1 cluster-induced SG characteristics

Other published reports suggest that dysregulated clustering of RBPs may have effects on SG formation and function, potentially by abnormally affecting cell stress responses, and we previously demonstrated that A1 clustering directly affects SG puncta formation (Molliex et al., 2015; Salapa et al., 2018; Gui et al., 2019; Anees et al., 2021; Clarke et al., 2021b).

**FIGURE 6:**
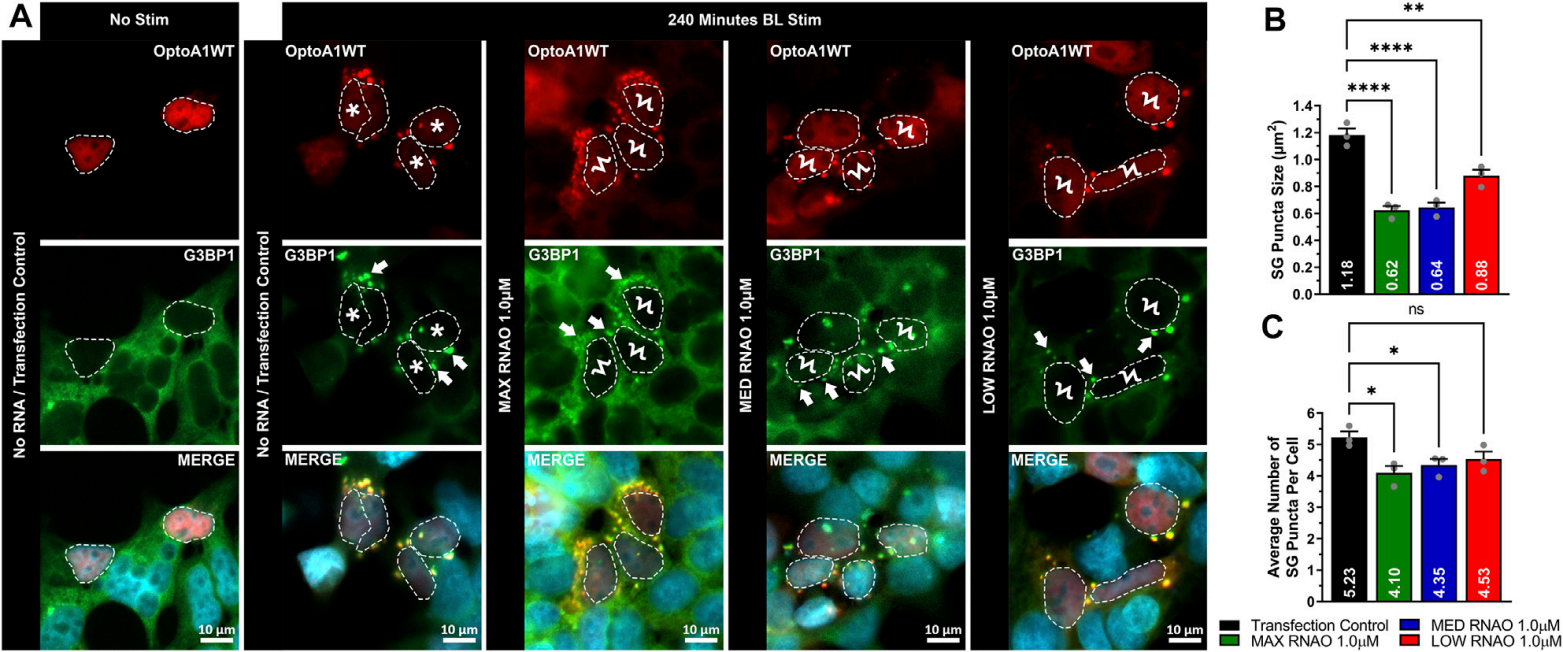
RNAOs affect the characteristics of SG puncta. **(A)** Representative images of OptoA1 blue light (BL) stimulated (240 min) cells containing SG puncta treated with and without the co-transfection of either 1.0 µM MAX RNAO, 1.0 µM MED RNAO or 1.0 µM LOW RNAO. * indicate cells that contain both few, large A1 clusters, and large SG puncta. Ϟ indicate cells that contain both abundant, small A1 clusters and small SG puncta. Arrows indicate the formation of SG puncta. Scale bars = 10 µm. Quantification of **(B)** SG puncta size and **(C)** SG puncta per cell, combined from three biological experiments. All results were analyzed using a one-way ANOVA, with a Tukey post-hoc test. Data shown are mean±S.E.M. for three biological replicates. *p < 0.05; ***p < 0.001; ****p < 0.0001; 95% Confidence Interval.

To determine whether attenuation of OptoA1 clustering with RNAO treatment had a physiological effect in cells, we next assessed whether there was a downstream effect on SGs. Here, we found that 1.0 µM RNAO concentrations altered SG morphology in BL induced OptoA1 clustering (Figure 6). Specifically, the effect of RNAOs on SG puncta size parallels their effects on A1 cluster size, resulting in significantly smaller SG puncta with MAX (reduced by 0.56 um^2^) and MED (reduced by 0.54 um^2^) RNAO treatments, as compared to TC, although the LOW RNAO had less of an impact (reduced by 0.30 um^2^) (Figures 6A,B). In contrast to the effect of RNAOs on A1 clusters, the average number of SG puncta per cell, in cells that also contained OptoA1 clusters, were significantly fewer with MAX and MED RNAO treatment (reduced by 1.13 and 0.88, respectively), while LOW RNAO treatment reduced them non-significantly by 0.70 puncta per cell (Figures 6A,C). To further assess whether these effects on SG puncta were due to RNAOs themselves, or whether they were due to RNAO modulation of A1 clustering, we also treated the cells with 1.0 µM MAX RNAO and induced SG formation with sodium arsenite treatment (Supplementary Figure S19). Our results demonstrate that cells treated with MAX RNAO, without sodium arsenite treatment, did not induce SG formation (Supplementary Figure S19). With sodium arsenite treatment, however, we found there was no significant difference in SG puncta size or average number of SG puncta per cell, indicating that RNAO treatment did not have an off-target, non-specific effect on SGs, and that our initial SG results were due to altered A1 clustering by RNAO treatment (Supplementary Figure S19). Altogether, this data suggests that RNAO modulation of OptoA1 clusters impacts the formation of SGs, and that larger OptoA1 clusters are the likely inducers of SG formation, while small OptoA1 clusters do not drive SG formation.

### RNAO treatment attenuates A1 cluster induced cell stress and improves cellular translation

**FIGURE 7:**
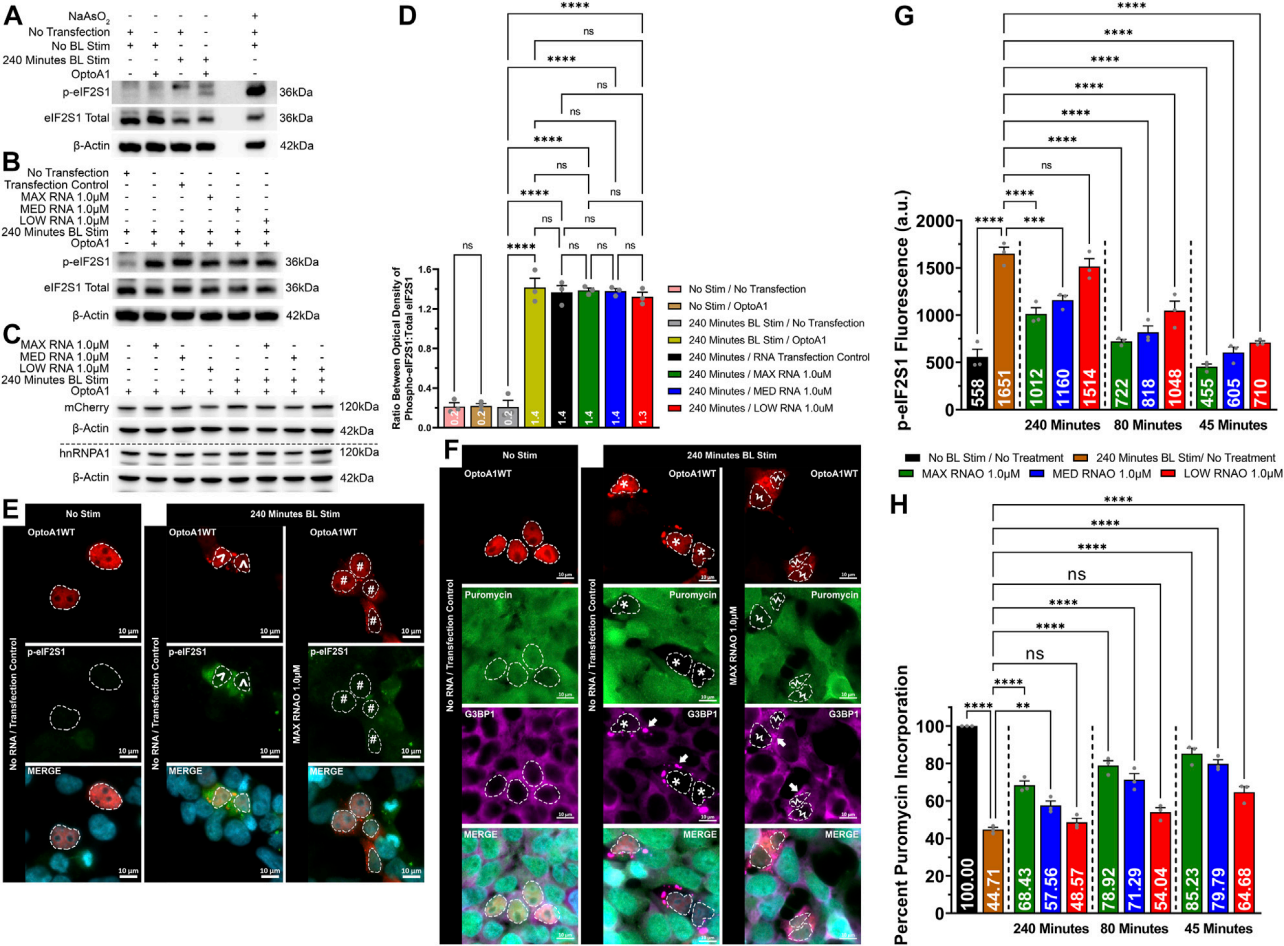
RNAO treatment attenuates OptoA1 cluster induced cell stress and improves cellular translation. OptoA1 transfected HEK293T cells were BL stimulated for 240 min either without RNAO **(A)** or with RNAO **(B)** co-transfection, protein was extracted, and Western blots were probed with phospho-eIF2S1, total eIF2S1 and β-actin antibodies. Additionally, the RNAO co-transfected samples were also probed with mCherry and hnRNPA1 antibodies to detect any changes in OptoA1 in the samples **(C)**. **(D)** Densitometric analysis of Western blots in **(A)** and **(B)**. Representative images of OptoA1 BL stimulated cells containing p-eIF2S1 **(E)** or puromycin incorporation **(F)** treated with and without the co-transfection of 1.0µM MAX RNAO. ^ indicate cells that contain few, large A1 clusters. # indicate cells that contain abundant, small A1 clusters. * indicate cells that contain both few, large A1 clusters, and large SG puncta. Ϟ indicate cells that contain both abundant, small A1 clusters and small SG puncta. Arrows indicate the formation of SG puncta. Scale bars = 10 µm. **(G)** Quantification of cell stress activation via p-eIF2S1 fluorescence expression at 45, 80 and 240 min of BL stimulation from cells transfected with 1.0 µM of either MAX, MED or LOW RNAO (images analyzed for MED and MIN RNAOs are like those in **(E)**, which are representative of MAX RNAO). **(H)** Quantification of cellular translation via percent puromycin incorporation from cells transfected with 1.0 µM of either MAX, MED or LOW RNAO (images analyzed for MED and MIN RNAOs are like those in **(E)**, which are representative of MAX RNAO). All results were analyzed using a one-way ANOVA, with a Tukey post-hoc test. Data shown are mean ± S.E.M. for three biological replicates. *p < 0.05; ***p < 0.001; ****p < 0.0001; 95% Confidence Interval.

We finally wanted to explore the connection between altered A1 clustering, attenuated SG puncta formation and characteristics, and the integrated cell stress response. We first determined that without RNAO treatment, A1 clustering induces cell stress, as measured by the expression of phosphorylated eIF2S1 (p-eIF2S1) via Western immunoblotting (Figures 7A,D; Supplementary Figure S20). To assess whether RNAO treatment attenuated this effect, we treated cells with 1.0 µM of either MAX, MED or LOW RNAO and measured the expression of p-eIF2S1 and found no significant difference between RNAO and non-treated samples (Figures 7B,D; Supplementary Figure S21). Additionally, we assessed whether these results were due to differences in the expression of OptoA1 in our samples and found no change in OptoA1 expression in any sample (Figure 7C; Supplementary Figure S22). Theorizing that the non-significant results of the RNAOs were because samples collected for Western blotting contained a mixture of transfected and non-transfected cells, we next assessed p-eIF2S1 expression via immunocytochemical imaging and analysis (Figure 7E). We analyzed cells that were a) transfected with OptoA1, b) formed OptoA1 clusters with BL stimulation and c) contained SGs. Additionally, we analyzed three separate time-points (45, 80 and 240 min) to assess the effect of RNAO treatment over time. These time-points were chosen as they aligned with the KA_1/2MAX_ of the RNAO treatments and the endpoint of the experiment (Figure 4). We compared the RNAO samples to the maximal p-eIF2S1 expression observed at 240 min (Figure 7G) and found that p-eIF2S1 expression was significantly decreased at all time-points with MAX or MED RNAO treatment, while LOW RNAO failed to show a significant difference only at 240 min (Figure 7G).

Since the inhibition of cellular translation is linked to the activation of cell stress, we also measured the activity of cellular translation via a puromycin incorporation assay in cells treated with 1.0 µM of either MAX, MED or LOW RNAO (Figures 7F,H). To initially assess whether RNAOs alone affected protein translation, we treated HEK293T cells with MAX RNAO without OptoA1 transfection (Supplementary Figure S23). In addition, the cells were treated with and without BL stimulation (Supplementary Figure S23). Our results demonstrate that the RNAOs alone did not affect protein translation, and without OptoA1 transfection, BL stimulation also did not affect protein translation (Supplementary Figure S23). From this, we experimentally analyzed cells that were a) transfected with OptoA1, b) formed OptoA1 clusters with BL stimulation and c) contained SGs. Furthermore, we analyzed three separate time-points (45, 80 and 240 min) to assess the effect of RNAO treatment over time. Again, these time-points were chosen as they aligned with the KA_1/2MAX_ of the RNAO treatments and the endpoint of the experiment (Figure 4). Without RNAO treatment, we found that A1 clustering significantly reduced cellular translation by ∼55% (Figures 7F,H). However, addition of the RNAOs significantly restored cellular translation (Figures 7F,H). Specifically, at 45 min of BL stimulation, all three RNAOs significantly improved cellular translation, by ∼30% on average (MAX = ∼40%; MED = ∼35%; LOW = ∼20%) (Figure 7H). By 80 min of BL stimulation, only MAX and MED RNAOs maintained a significant increase in translation, again by ∼30% on average (MAX = ∼34%; MED = ∼27%) (Figure 7H). Finally, at 240 min, again, MAX and MED RNAOs showed a significant increase in translation by ∼18% on average (MAX = ∼24%; MED = ∼13%) (Figure 7H).

These results demonstrate that A1 clustering induces cell stress, but by attenuating its self-interaction using RNAOs, we observed a significant reduction in cell stress, as measured by p-eIF2S1 expression, and improved cellular translation, as measured by puromycin incorporation (Figure 7).

## Discussion

In this study, we examined the effects of RNAOs on A1 self-association and the downstream molecular mechanisms perturbed by A1 dysfunction. To accomplish this, we utilized an optogenetically inducible A1 self-association system that we previously established, allowing us to model and examine A1 dysfunction and its downstream cellular effects (Clarke et al., 2021b). These experiments are important because they allow for the study of dysregulated proteins *in vitro* and further characterizes the role that dysfunctional RBPs perform in altering cellular outcomes. Since previous studies have established an interaction between RNA and A1 and have described the influence of RNA on an aspect of A1 self-interaction known as liquid-liquid phase separation (LLPS), we tested the effect of RNAOs on A1 dysfunction and its physiological impacts in the cell (Molliex et al., 2015; Jain et al., 2017). To our knowledge, this study is the first to examine RNAOs as a tool to modulate self-association for the prevention of full-length, wild-type A1 dysfunction. This work aligns with recent studies that use different methods to study how endogenous cellular materials (e.g., RNA, nuclear-import receptors) can prevent and reverse the aggregation of RBPs (Guo et al., 2018; Van Treeck and Parker, 2018; Mann et al., 2019; Hutten et al., 2020).

Combining optogenetics, structural modelling, and thermodynamic protein stability analysis, we demonstrate that treatment of wild-type A1 with sequence- and structure-specific RNAOs attenuated A1 self-association cluster kinetics. To note, in these *in silico* and *in vitro* readouts we did not directly assess endogenous functionality of A1 (e.g., changes in RNA metabolism; expression and fold changes in target RNAs), nor did we describe the structure of purified A1. Additionally, only the N-terminal UP1of A1 has been structurally defined to date, as the disordered nature of the C-terminal portion of A1 has proven difficult to analyze confidently using protein structural analysis methods (Sun et al., 2020). Since A1 is not an enzyme, conventional *in vitro* enzymatic readouts are difficult to apply and interpret. Cellular readouts (e.g., changes in RNA metabolism), however, are best applied to assess functionality changes. Further, we were intently interested in assessing binding of RNAOs to A1, to further assess self-association prevention in cells. However, as A1 is an RBP, its binding to RNAOs is a readout of functionality; if the protein were non-functional, it should not associate with RNA, and thus not interact with RNAOs. This readout is demonstrated by our PrLD results (Figure 2), which confirmed that the RNAOs were only binding and altering the structure of A1 containing the A1 RRM domains (Figure 2). Future studies using our system and RNAOs, however, will assess functionality changes in A1 in cells, with a focus on changes in RNA metabolism.

In live cells, we explored a continuum of RNA sequences and structures to modulate A1 clustering. HEK TOTAL RNA, which is neither sequence- nor structure-specific for A1, showed the lowest A1 cluster formation shift (Figure 1). This was followed by moderate A1 cluster formation shifts due to the highly structured IRES RNA, where its secondary hairpin and stem-loop structures are known to drive its interaction with A1, rather than its sequence (Figure 1). Since other RBPs have been shown to also interact with this RNA, IRES RNA was not an ideal tool to directly assess the impact of RNA binding to A1 alone on A1 self-association (Ramos et al., 2022a; Ramos et al., 2022b). In contrast, the MED and MAX RNAOs, which use a hairpin structure to present an RNA loop with high sequence specificity for A1, showed the most robust effects on A1 cluster formation, with MAX RNAO showing both the strongest affinity in our molecular dynamics simulations, and displaying the greatest shift in A1 cluster formation, increasing the KA_1/2MAX_ by 48.5 min (Figure 2; Figure 3; Figure 4). Interestingly, LOW RNAO A1 cluster formation attenuation was not as effective as IRES RNA treatment (Figure 2; Figure 3; Figure 4; Supplementary Figure S17). Since IRES RNA has a complex secondary structure, with multiple hairpin loops that have not yet been tested in isolation with A1, we cannot completely rule out the possibility that the other hairpin loops may interact with A1 in a sequence-specific manner and not just by structure, which may describe the discrepancy in the results between LOW RNAO and IRES RNA (Gendron et al., 2011; Vallejos et al., 2012; Barrera et al., 2020). Conversely, the results may also be based solely upon the binding efficiency of LOW RNAO to A1, as its binding free energy was found to be weaker than MED and MAX RNAO (Figure 3; Table 1; Supplementary Figure S9), and as found in a previous report, its dissociation constant was the lowest for all tested RNAO sequences (Jain et al., 2017). In addition, based upon preliminary results (data not shown), the effect of RNAO treatment is predominantly on the initial clustering of A1 and not on the dissociation of pre-formed A1 clusters, as the dissociation kinetics of A1 did not change utilizing the treatment paradigm described in this study. This, however, requires further testing, as either a subsequent treatment with RNAOs may be required, or its addition may need to be timed accordingly with the ending of A1 association. Finally, we demonstrate that the effects observed in this study with RNAOs are due to a direct interaction with A1. With mutation of ARG55 to ALA55 in RRM1 of A1 (R55A), we observed no significant alteration in cluster formation kinetics with RNAO treatment (Supplementary Figure S18). Overall, our results confirm that RNAO sequence and binding plays an integral role in attenuating A1 dysfunction.

To note, with MAX and MED RNAO treatments, we observed an attenuation in the reduction of A1 cluster formation with our highest concentration (2.0 µM), as compared to our middle concentration (1.0 µM) (Supplementary Figures S14, S15). However, for MAX RNAO, this reduction was still significantly different from our controls. To elaborate on this discrepancy, current studies suggests that an RNA concentration threshold dictates whether RNA will prohibit or promote A1 self-association (Martin et al., 2021; Ritsch et al., 2022; Tejedor et al., 2022). Specifically, current publications suggest the low complexity domain (LCD) of A1 interacts with its RRMs, and RNA binding attenuates this interaction (Martin et al., 2021; Tejedor et al., 2022). In such a case, the LCD would be exposed to the aqueous cytoplasmic environment, and undergo LLPS, thereby forming A1 clusters (Ritsch et al., 2022). However, our results from this study show that at low concentrations of RNA (i.e., 1.0 and 0.5 µM) attenuate A1 clustering, suggesting that the proposed above mechanism may not be occurring, or may be occurring at a rate that is not affecting overall A1 clustering. At higher concentrations (i.e., 2.0 µM), the attenuation effects of RNA may be superseded by the LLPS effects of RNA binding. Future studies would need to be performed to assess such a threshold effect.

Based upon a thermodynamic study showing that high concentrations of ATP binding to A1 can cause a change in A1 structural stability, we theorize and show supporting data that our observed shift in A1 cluster formation is likely due to structural changes in A1 upon binding of RNAOs (Dang et al., 2021) (Figure 2; Figure 3; Supplementary Figure S1–S3, Supplementary Figure S8, Supplementary Figures S10–S12). We found that binding of the RNAOs to A1 significantly altered the melting temperature of A1 indicating a shift in A1 structural stability (Figure 2). We demonstrate that this was RNAO-sequence-specific, as MAX RNAO induced the largest shift creating the most stabilized version of A1, followed by MED RNAO, and with LOW RNAO having no effect. Additionally, we demonstrate that this effect is due to the RNAOs binding to the UP1 (or RRM domains) of A1, as in isolation, RNAOs did not influence the melting temperature of the PrLD of A1 (Figure 2). We cannot, however, fully rule out an effect by the PrLD in full-length wild-type A1, as this region contains an intrinsically disordered RGG (glycine-arginine rich) motif-containing domain that has been shown to bind to RNA (Ozdilek et al., 2017; Ghosh and Singh, 2018; Ghosh and Singh, 2020). To understand the molecular factors that cause variability in the binding affinity and thermal shift response of RNAOs in this study, we built molecular models of RNAO-bound A1 RRM complexes and probed their dynamic interactions stabilizing the complexes (Figure 3; Table 1; Supplementary Figures S1–S3, Supplementary Figures S6–S12). We noted that MAX and LOW RNAOs exhibited their binding affinities by engaging with the RRM1 binding site that is formed by its RNPs and the RRM1/2 linker loop (Figure 2; Supplementary Figure S8). Additionally, MAX RNAO with an AGG nucleotide motif engaged more native-like contacts with RNPs in RRM1, whereas MED RNAO interacted predominantly with the RRM1/2 linker loop thus exhibiting weaker affinity than the former (Figure 2; Supplementary Figure S8). LOW RNAO, which lacks the AG nucleotide motif, did not interact with the binding pocket and their affinity in the models mostly came from non-specific electrostatic interactions (Figure 2; Supplementary Figure S8). These results together indicate that it is possible to target A1 RRMs through structure- and sequence-specific RNAOs.

When we examined the characteristics of A1 clusters in cells with RNAO treatment, we found they were significantly smaller and more numerous (Figure 5). These observations are supported in two ways. Firstly, a recent report established there is a salt-sensitive electrostatic interaction between the UP1 region and the PrLD of A1, and that this interaction influences A1 LLPS, and thus potentially self-association (Martin et al., 2021). Briefly, the authors found that their conformational ensembles demonstrated extensive UP1-PrLD interaction at low sodium chloride concentrations that were progressively disrupted at higher concentrations, suggesting that increasing salt concentrations shift the equilibrium between conformations where the PrLD associates with the UP1 and conformation where the PrLD is unassociated (Martin et al., 2021). Although we cannot ascertain whether RNAO binding to A1 enhances or attenuates this intramolecular interaction specifically, we theorize that RNAO binding influences this interaction in some way, which results in smaller and increased numbers of clusters. Secondly, it is well established that protein condensates mature over time, initially forming smaller, soluble species that eventually develop into larger, insoluble aggregates (Lin et al., 2015). Our data show similar results, wherein we hypothesize that the smaller and greater numbers of A1 clusters may represent intermediate protein condensates that have been prohibited to form larger, insoluble aggregates due to RNAO binding.

It is theorized that RBP dysregulation affects SG biology, but the mechanisms through which this is controlled are still not completely characterized (Vanderweyde et al., 2013; Wolozin and Ivanov, 2019; Baradaran-Heravi et al., 2020; Marcelo et al., 2021). Previous evidence indicates that A1 influences SG formation through unknown pathways (Guil et al., 2006; Molliex et al., 2015; Douglas et al., 2016; Purice and Taylor, 2018; Salapa et al., 2018; Anees et al., 2021; Clarke et al., 2021b). Additionally, we previously found that OptoA1 clustering influences SG formation, and that mutations in the PrLD of A1 increase both A1 clustering and SG formation (Clarke et al., 2021b). In this study, we re-assessed the interaction between OptoA1 and SG formation following treatment with RNAOs. We initially show that in addition to altering aspects of A1 cluster formation, RNAO treatment of A1 also alters SG formation (Figure 6). Particularly, we found smaller and fewer SG puncta per cell, and fewer cells had both A1 clusters and SG puncta. This also leads us to suggest that the smaller and more numerous A1 clusters formed in the presence of RNAOs are likely to be less pathogenic since they induce fewer SGs. To confirm that our SG observations were not due to an off-target effect with RNAO treatment, we performed experiments with RNAOs and SGs only and found that RNAOs do not induce SG formation, and have no effect on SG characteristics, further demonstrating the effects we observed were due to A1 cluster attenuation with RNAOs (Supplementary Figure S19).

Based upon these observations, we next assessed whether A1 clustering affected the activation of cell stress, and whether RNAO treatment perturbed the effect of A1 clustering on cell stress (Figure 7). It is theorized that dysregulated protein condensates induce cell stress and trigger erroneous SG formation, and therefore we hypothesized that A1 clusters may induce cell stress, possibly by activating molecular stress pathways. In this model, continued aberrant formation and accumulation of A1 clusters causes sustained cell stress, leading to the continual formation of SGs. Recent reports from our lab and others corroborate this idea, as we have shown that a decrease in A1 expression in Neuro-2A cells resulted in a reduction of SGs that formed with sodium arsenite induced cell stress, and in an A1 depleted HeLa cell line, osmotic cell stress resulted in less viable and poorly recovered cells, likely due to an inability to form SGs (Guil et al., 2006; Anees et al., 2021). From our results in this report, we show that A1 clustering induces cell stress (Figure 7). With the addition of RNAOs during A1 cluster formation, we demonstrate that cell stress is attenuated and cellular translational activity, which is downstream of cell stress activation, is improved (Figure 7). Interestingly, if compared together, both results show an inverse correlation (i.e., increased p-eIF2S1/decreased protein translation), which is an innate feature of the integrated stress response. Furthermore, this data illustrates the differing capacity of the three RNAOs to attenuate the integrated stress response, with MAX RNAO eliciting the longest attenuation (Figure 7). Together, these results demonstrate that RNAO treatment improves cellular function by altering the formation of A1 clusters, and in turn, the formation of SGs. However, it is unclear if A1 clusters directly activate cell stress mechanisms, or they affect SG formation that then activates downstream cell stress mechanisms. Although mechanistically unknown, we propose that RNAOs affect A1 LLPS, which in turn influences the LLPS and formation of SGs. This hypothesis is strengthened by a report that found RNA facilitates the LLPS of A1 using phase droplet separation, and that transient expression of the A1 PrLD in HeLa cells resulted in an increased cytoplasmic concentration of A1, and an observed increase in the assembly of SGs (Molliex et al., 2015). Additionally, previous reports have suggested that SG formation is driven by concentration-dependent A1 LLPS that may require both a cytoplasmic A1 protein concentration threshold to form and may be enhanced by clustered forms of A1 (Guil et al., 2006; Molliex et al., 2015; Gui et al., 2019; Hofmann et al., 2021).

In summary, our data provide novel methodological and mechanistic insights into how sequence- and structure-specific RNAOs bind A1, influence the self-associative properties of A1, and how the downstream effects of A1 dysregulation can be attenuated by perturbing A1 clustering using RNAOs, thereby creating potential therapies designed to decrease the deleterious effects of A1 dysfunction on cells.

## Data Availability

The original contributions presented in the study are included in the article/Supplementary Material, further inquiries can be directed to the corresponding authors.

## Supplementary material

Supplementary Table S1 DNA Primer and RNA Oligonucleotide Sequences.

Supplementary Figure S1 The docking poses and their respective scores for MAX RNAO against A1 RRM. **(A)** All the complex poses obtained from docking are superposed, while showing all the top RNAO poses and only single RRM1 (grey ribbon). The docking scores of all the poses are shown in the embedded table. The complex coloured in green (complex 5) in this table is chosen for further analyses as it was the first top pose that exhibited native contacts against A1 RRM **(B)** when compared to the other poses. The orientation for complex 1, a false-positive hit, exhibiting non-specific binding of RNAO is also shown **(C)**.

Supplementary Figure S2 The docking poses and their respective scores for MED RNAO against A1 RRM. **(A)** All the complex poses obtained from docking are superposed, while showing all the top RNAO poses and only single RRM1 (white ribbon). The docking scores of all the poses are shown in the embedded table. The complex coloured in green (complex 8) in this table is chosen for further analyses as it was the first top pose that exhibited native contacts against A1 RRM **(B)** when compared to the other poses. The orientation for complex 1, a false-positive hit, exhibiting non-specific binding of RNAO is also shown **(C)**.

Supplementary Figure S3 The docking poses and their respective scores for LOW RNAO against A1 RRM. **(A)** All the complex poses obtained from docking are superposed, while showing all the top RNAO poses and only single RRM1 (white ribbon). The docking scores of all the poses are shown in the embedded table. The complex coloured in green (complex 12) in this table is chosen for further analyses as it was the first top pose that exhibited native contacts against A1 RRM **(B)** when compared to the other poses. The orientation for complex 1, a false-positive hit, exhibiting non-specific binding of RNAO is also shown **(C)**.

Supplementary Figure S4 Stacking interactions in the MAX RNAO-RRM model compared to the previously reported structures in PDB. **(A)** Superposed binding poses of oligonucleotide- A1 RRM complexes from crystal structures in PDB (4YOE[28], 1PGZ[55], 1PO6[55], 1U1K[56] and 2UP1[54]) revealed a pair of highly conserved aromatic stacking interactions rendered by an adenine nucleotide with PHE17 and HIS101, and a guanine with PHE59 in A1 RRM1. A, G, T = nucleotides. **(B)** 3D binding poses of the modelled MAX-A1 RRM complex confirmed that this complex formed the two conserved stacking interactions through PHE17-A13-HIS101 network and the G15-PHE19 pair.

Supplementary Figure S5 Quantification of OptoA1cluster formation during 240-minute BL stimulation with the addition of HEK TOTAL RNA at three concentrations (1.0 µg, 0.5 µg or 0.25 µg). **(A)** Results are plotted as a percent maximum to the highest cluster response at 240 minutes for each RNA treatment, resulting in kinetics curves for association dynamics. No Treatment = no treatment with RNA; Transfection Control = cells only transfected with RNAiMAX. Dashed lines indicate KA_1/2Max_. A^I^) Tabular results of a two-way ANOVA, with a Bonferroni post-hoc test from the curves illustrated in **(A)**. A^II^) Bar graphs and one-way ANOVA, with a Tukey post-hoc test analysis of KA_1/2Max_ from the curves illustrated in **(A)**. Data shown are mean ± SEM for three biological replicates. **p* < 0.05; ***p* < 0.01; ****p* < 0.001; *****p* < 0.0001; 95% Confidence Interval.

Supplementary Figure S6 Quantification of OptoA1 cluster formation during 240-minute BL stimulation with the addition of IRES RNA at three separate concentrations (2.0 µM, 1.0 µM or 0.5 µM). **(A)** Results are plotted as a percent maximum to the highest cluster response at 240 minutes for each RNA treatment, resulting in kinetics curves for association dynamics. No Treatment = no treatment with RNA; Transfection Control = cells only transfected with RNAiMAX. Dashed lines indicate KA_1/2Max_. A^I^) Tabular results of a two-way ANOVA, with a Bonferroni post-hoc test from the curves illustrated in **(A)**. A^II^) Bar graphs and one-way ANOVA, with a Tukey post-hoc test analysis of KA_1/2Max_ from the curves illustrated in **(A)**. Data shown are mean ± SEM for three biological replicates. **p* < 0.05; ***p* < 0.01; ****p* < 0.001; *****p* < 0.0001; 95% Confidence Interval.

Supplementary Figure S7 Evolution of RMSD of the RNAO-A1 complexes. The stability of the complexes during MD simulation was assessed by plotting the evolution of backbone RMSD of A1 RRMs (on the left-side panel) and all the atoms of RNAOs (on the right-side panel). As seen in the plots on the left, the protein has much lower RMSD values than those of RNAOs (right) for all three complexes. RNAOs underwent more conformation changes to adapt to its binding with A1 RRMs that was stable during simulation.

Supplementary Figure S8 Overview of the interactions contributing significantly to the stabilization of each RNAO molecule in the RRM1 binding pocket. **(A)** As summarized in Figure 3 and Supplementary Figure S12, the MAX RNAO has the greatest number of pi-pi stacking as well as electrostatic interactions that are responsible for its highest binding-free energy value. This is attributed to the two AG motifs available within the apical loop for native RNA-RRM1 contacts. **(B)** The MED RNAO, although having fewer interactions than MAX, is still able to form some pi-pi contacts as it still has an AG motif in the RNP site, as well as some notable hydrogen bonds. **(C)** Since the LOW RNAO lacks an AG motif, it is not able to form the signature interactions as noted for MAX or MED RNAOs. However, it is still able to bind using nonspecific contacts from the rest of the RNA. This is also apparent with the apical loop residues forming self-stacking interactions and not being available for extensive interactions with RNPs other than G13.

Supplementary Figure S9 Energy decomposition plots show the key nucleotides from RNAOs that interact with A1 RRM. The per-nucleotide energy decomposition analyses showed that most of the seven-nucleotide long apical loop (11–17 nucleotide positions) in MAX RNAO contributed to the free energy of its complex with RRMs (top-panel). Only adenines and guanines contributed to the interactions of MAX RNAO with RRMs. Wherein, the MED RNAO-RRM complex (in the middle panel), only U14 and G16 from the apical loop of RNAO contributed significantly to the complex stability. In the bottom-panel, only G13 nucleotide from the LOW RNAO made a significant contribution to its complex with RRMs; other nucleotides exhibited a marginal role in this process.

Supplementary Figure S10 Illustration of the prominent electrostatic interactions in the MAX RNAO-A1 complex. **(A)** A 3D representation of key salt-bridge and hydrogen bond interactions between nucleotides such as G14, G15 and G17 against ARG55, GLN12 and ARG92, respectively. The selected amino acids and nucleotides are shown as stick representations as the other segments of the binding pose are shown as cartoon representations in the background. Evolution of distances between the side-chain carbonyl group of GLN12 and purine ring of G15 **(B)** confirmed that these interactions formed after ∼60 ns and remained mostly stable until the end of simulation. Similarly, the distance evolution between the side-chain amino groups of ARG55 and phosphate group of G15 **(C)** described that this pair established a salt-bridge (N-OP1 shown in orange) and a hydrogen bond (NH-OP1 shown in green) after 20 ns and maintained them throughout the course of MD simulation.

Supplementary Figure S11 Illustration of the prominent electrostatic interactions in the MED RNAO-A1 complex. 3D representations of key salt-bridge and hydrogen bond interactions between key nucleotides from MED RNAO and amino acids from RRMs are shown in A–B. ARG53 and ARG55 formed dynamic salt-bridge contacts with A7 and U8 nucleotides from MED RNAO **(A)**; while the side-chain amino group of LYS87 and backbone of THR103 made hydrogen bond contacts with A15 and U14, respectively, **(B)**. The evolution of distance between the backbone carbonyl oxygen atom in THR103 and pyrimidine ring of U14 confirmed the stability of their hydrogen bond during MD simulation.

Supplementary Figure S12 The total number of hydrogen bonds calculated for the MAX, MED, and LOW RNAO-A1 complexes. While MAX and MED RNAOs display similar number of hydrogen bonds, LOW RNAO has reduced capacity for them.

Supplementary Figure S13 RNAOs ubiquitously transfect into HEK293T cells, regardless of OptoA1 transfection. Representative images of HEK293T cells transfected with OptoA1, and MAX RNAO tagged with digoxigenin (DIG). Top Panel: MAX RNAO DIG was detected using primary mouse monoclonal anti-digoxigenin and secondary mouse monoclonal Alexa Fluor 488. Bottom Panel: For negative control, background only cells, cells were transfected with MAX RNAO DIG, but were only incubated with secondary mouse monoclonal Alexa Fluor 488. Dashed lines outline cellular nuclei in OptoA1 transfected cells. Scale bars = 10 µm.

Supplementary Figure S14 Quantification of OptoA1 cluster formation during 240-minute BL stimulation with the addition of MAX RNAO at three concentrations (2.0 µM, 1.0 µM or 0.5 µM). **(A)** Results are plotted as a percent maximum to the highest cluster response at 240 minutes for each RNA treatment, resulting in kinetics curves for association dynamics. No Treatment = no treatment with RNA; Transfection Control = cells only transfected with RNAiMAX. Dashed lines indicate KA_1/2Max_. A^I^) Tabular results of a two-way ANOVA, with a Bonferroni post-hoc test from the curves illustrated in **(A)**. A^II^) Bar graphs and one-way ANOVA, with a Tukey post-hoc test analysis of KA_1/2Max_ from the curves illustrated in **(A)**. Data shown are mean ± SEM for three biological replicates. **p* < 0.05; ***p* < 0.01; ****p* < 0.001; *****p* < 0.0001; 95% Confidence Interval.

Supplementary Figure S15 Quantification of OptoA1 cluster formation during 240-minute BL stimulation with the addition of MED RNAO at three concentrations (2.0 µM, 1.0 µM or 0.5 µM). **(A)** Results are plotted as a percent maximum to the highest cluster response at 240 minutes for each RNA treatment, resulting in kinetics curves for association dynamics. No Treatment = no treatment with RNA; Transfection Control = cells only transfected with RNAiMAX. Dashed lines indicate KA_1/2Max_. A^I^) Tabular results of a two-way ANOVA, with a Bonferroni post-hoc test from the curves illustrated in **(A)**. A^II^) Bar graphs and one-way ANOVA, with a Tukey post-hoc test analysis of KA_1/2Max_ from the curves illustrated in **(A)**. Data shown are mean ± SEM for three biological replicates. **p* < 0.05; ***p* < 0.01; ****p* < 0.001; *****p* < 0.0001; 95% Confidence Interval.

Supplementary Figure S16 Quantification of OptoA1 cluster formation during 240-minute BL stimulation with the addition of LOW RNAO at three concentrations (2.0 µM, 1.0 µM or 0.5 µM). **(A)** Results are plotted as a percent maximum to the highest cluster response at 240 minutes for each RNA treatment, resulting in kinetics curves for association dynamics. No Treatment = no treatment with RNA; Transfection Control = cells only transfected with RNAiMAX. Dashed lines indicate KA_1/2Max_. A^I^) Tabular results of a two-way ANOVA, with a Bonferroni post-hoc test from the curves illustrated in **(A)**. A^II^) Bar graphs and one-way ANOVA, with a Tukey post-hoc test analysis of KA**1/2Max** from the curves illustrated in **(A)**. Data shown are mean ± SEM for three biological replicates. **p* < 0.05; ***p* < 0.01; ****p* < 0.001; *****p* < 0.0001; 95% Confidence Interval.

Supplementary Figure S17 Comparison of OptoA1 KA_1/2MAX_ during 240-minute BL stimulation with the addition of HEK TOTAL RNA, IRES RNA, MAX RNAO, MED RNAO, or LOW RNAO. Bar graphs and one-way ANOVA, with a Tukey post-hoc test analysis of KA_1/2Max_ from the curves illustrated in Supplementary Figures S5,6,14,15,16. Data shown are mean ± SEM for three biological replicates. **p* < 0.05; ***p* < 0.01; ****p* < 0.001; *****p* < 0.0001; 95% Confidence Interval.

Supplementary Figure S18 Mutation of RRM1 inhibits RNAO binding. Representative images of blue light (BL) stimulated R55A OptoA1 mutant cells with either **(A)** No Treatment or **(B)** treated with 1.0 µM MAX RNAO. **(C)** Quantification of A1 cluster formation during a 240-minute BL stimulation protocol with No Treatment (Green) or MAX RNAO (Red). Results are plotted as a percent maximum to the highest cluster response at 240 minutes for each RNA treatment, resulting in a kinetics curve for association dynamics. No Treatment OptoA1 wild-type cells (Brown); Transfection Control OptoA1 wild-type cell (Black) = cells only transfected with RNAiMAX. Dashed lines indicate KA_1/2Max_. C^I^) Tabular results of a two-way ANOVA, with a Bonferroni post-hoc test from the curves illustrated in **(C)**. C^II^) Bar graphs and one-way ANOVA, with a Tukey post-hoc test analysis of KA_1/2Max_ from the curves illustrated in **(C)**. Data shown are mean ± SEM for three biological replicates. **p* < 0.05; ***p* < 0.01; ****p* < 0.001; *****p* < 0.0001; 95% Confidence Interval.

Supplementary Figure S19 RNAOs do not affect SG formation nor SG puncta characteristics. **(A)** Representative images of untreated HEK293T cells transfected with 1.0 µM MAX RNA. **(B)** Representative images of NaAsO_2_ treated HEK293T cells transfected with and without 1.0 µM MAX RNA. Arrows indicate the formation of SG puncta. Scale bars = 10 µm. Quantification of **(C)** average SG puncta size and **(D)** average number of SG puncta per cell, from one biological experiment. All results analyzed using a one-way ANOVA, with a Tukey post-hoc test. Data shown are mean ± SEM.

Supplementary Figure S20 Full, unedited Western immunoblot images from Figure 7A. Red star indicates the bands corresponding to phospho-eIF2S1 and eIF2S1 Total (36kDa). To note, the Western immunoblots were probed sequentially: phospho-eIF2S1 → eIF2S1 Total → β-Actin.

Supplementary Figure S21 Full, unedited Western immunoblot images from Figure 7B. Red star indicates the bands corresponding to phospho-eIF2S1 and eIF2S1 (36kDa). To note, the Western immunoblots were probed sequentially: phospho-eIF2S1 → eIF2S1 Total → β-Actin.

Supplementary Figure S22 Full, unedited Western immunoblot images from Figure 7C. Bands corresponding to OptoA1—120kDa; bands corresponding to endogenous A1—36kDa. To note, the Western immunoblots were probed sequentially: mCherry → β-Actin; hnRNPA1 (4B10) → β-Actin.

Supplementary Figure S23 MAX RNAO treatment alone does not perturb protein translation. Representative images of HEK293T cells transfected with MAX RNAO, without OptoA1 transfection, probed for puromycin incorporation and treated with (Bottom Panel) and without (Top Panel) BL stimulation for 240 minutes. Dashed lines outline cellular nuclei. Scale bars = 10 µm.

Supplementary Video S1 No treatment, OptoA1 cluster formation over the time-course of a BL experimental paradigm.

Supplementary Video S2 Transfection control, OptoA1 cluster formation over the time-course of a BL experimental paradigm.

Supplementary Video S3 HEK TOTAL RNA treated, OptoA1 cluster formation over the time-course of a BL experimental paradigm.

Supplementary Video S4 IRES RNA treated, OptoA1 cluster formation over the time-course of a BL experimental paradigm.

Supplementary Video S5 MAX RNAO treated, OptoA1 cluster formation over the time-course of a BL experimental paradigm.

Supplementary Video S6 MED RNAO treated, OptoA1 cluster formation over the time-course of a BL experimental paradigm.

Supplementary Video S7 LOW RNAO treated, OptoA1 cluster formation over the time-course of a BL experimental paradigm.
