# Supplementary material for: Sequence- and structure-specific RNA oligonucleotide binding attenuates heterogeneous nuclear ribonucleoprotein A1 dysfunction
Source: Front Mol Biosci. 2023 Jun 22;10:1178439. doi: 10.3389/fmolb.2023.1178439 (PMC10325567; doi:10.3389/fmolb.2023.1178439)
Supplement: Supplementary file 3 [file Presentation1.pptx]

## Slide 1
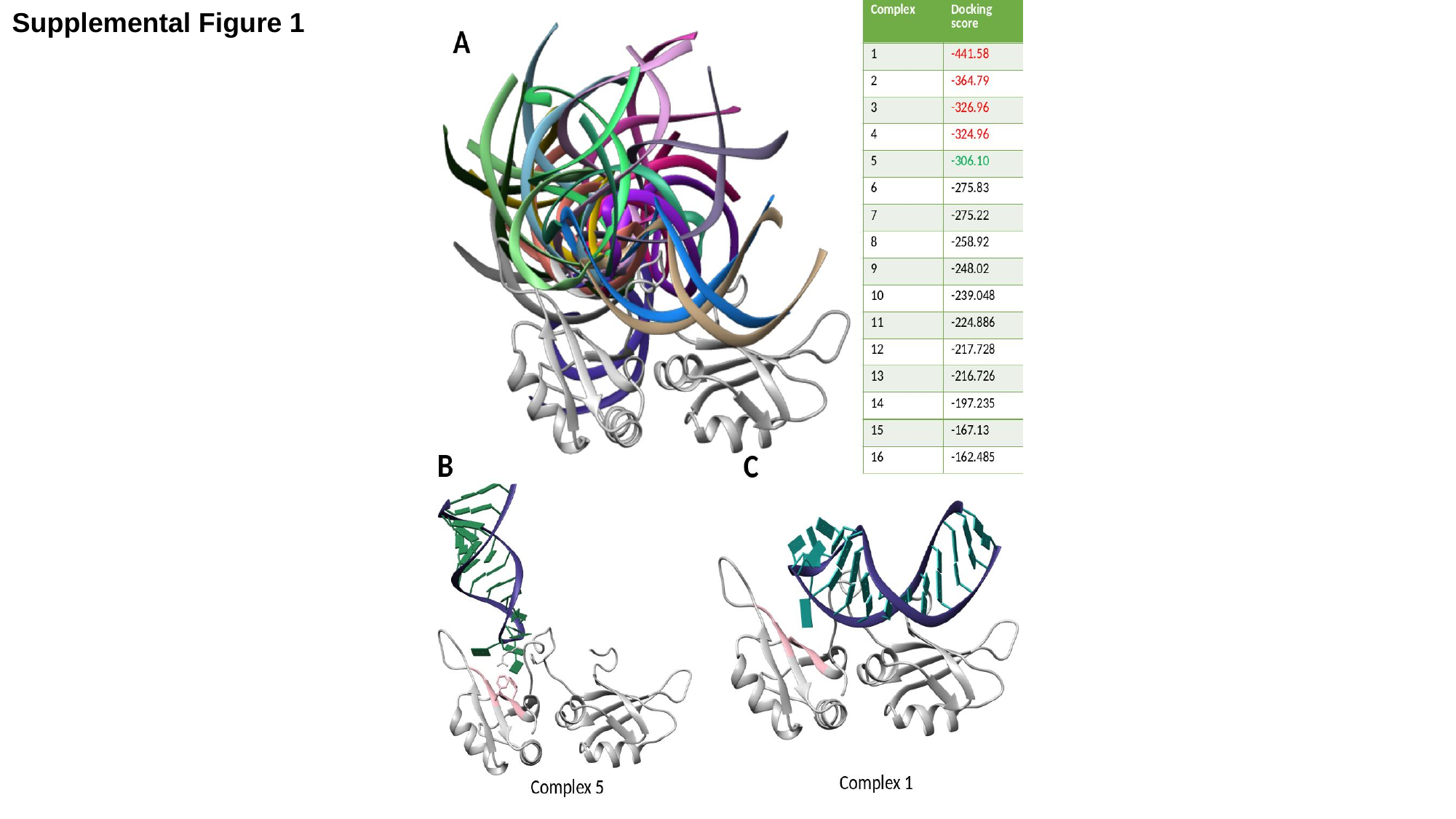

Supplemental Figure 1

## Slide 2
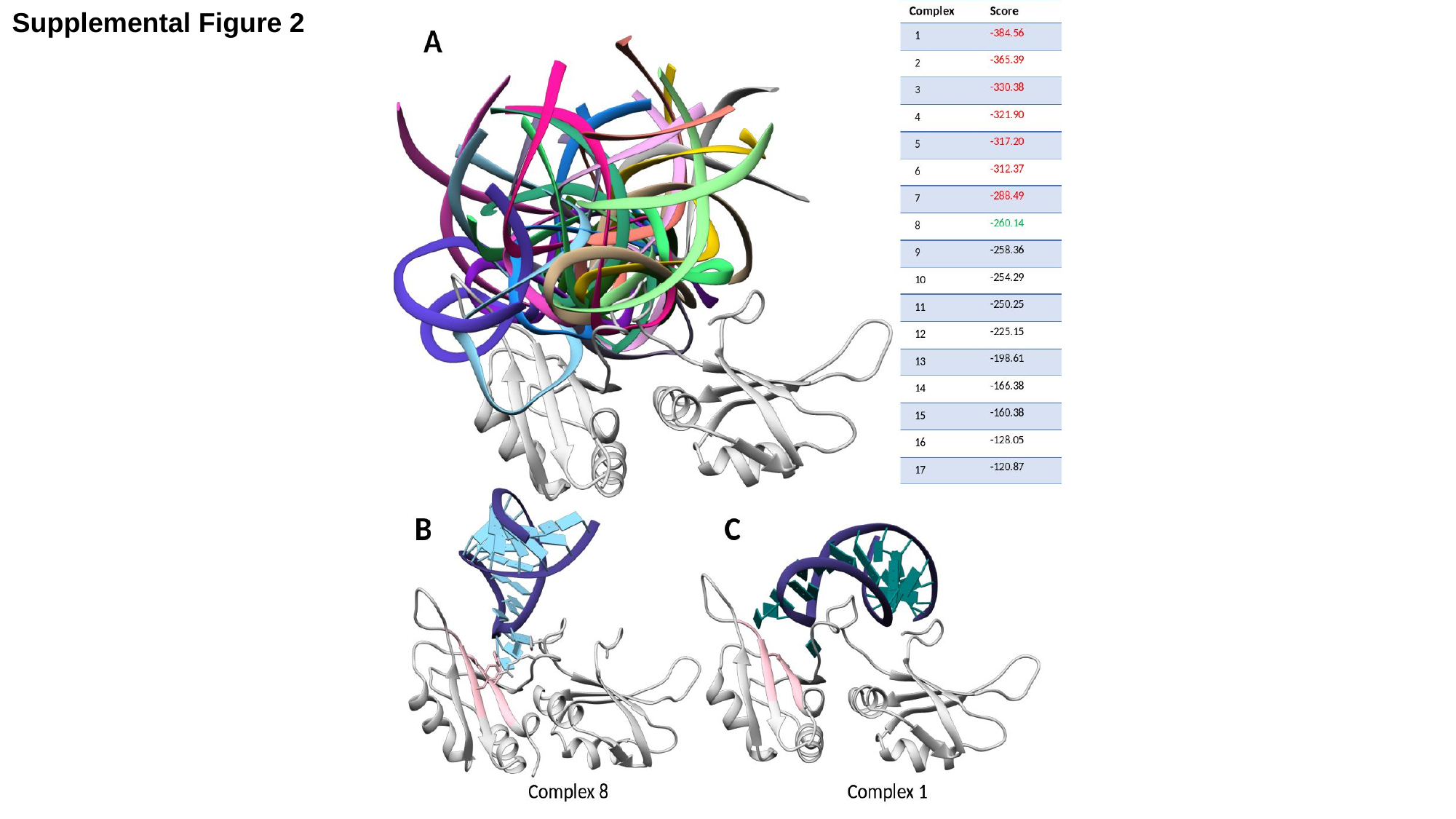

Supplemental Figure 2

## Slide 3
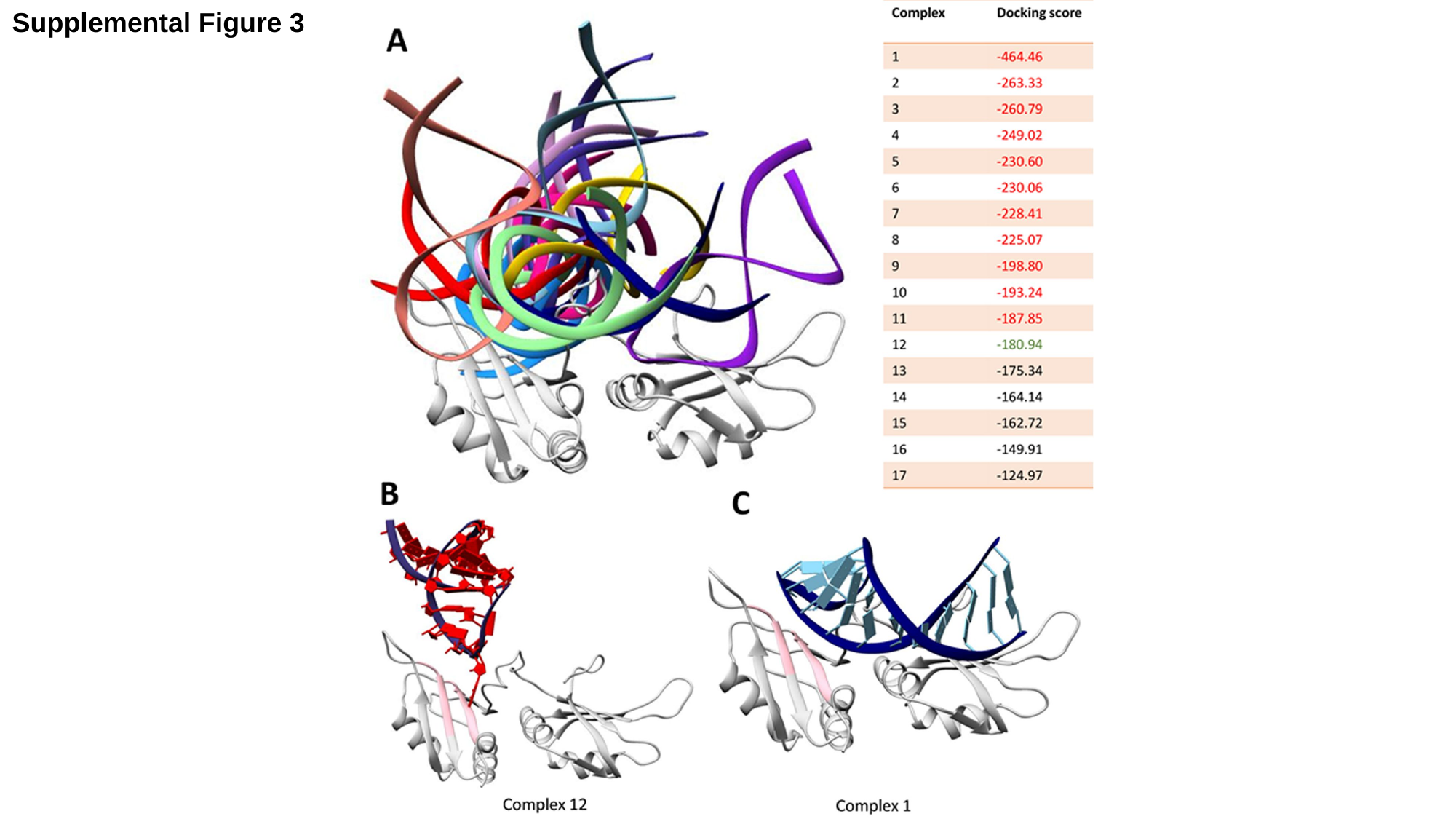

Supplemental Figure 3

## Slide 4
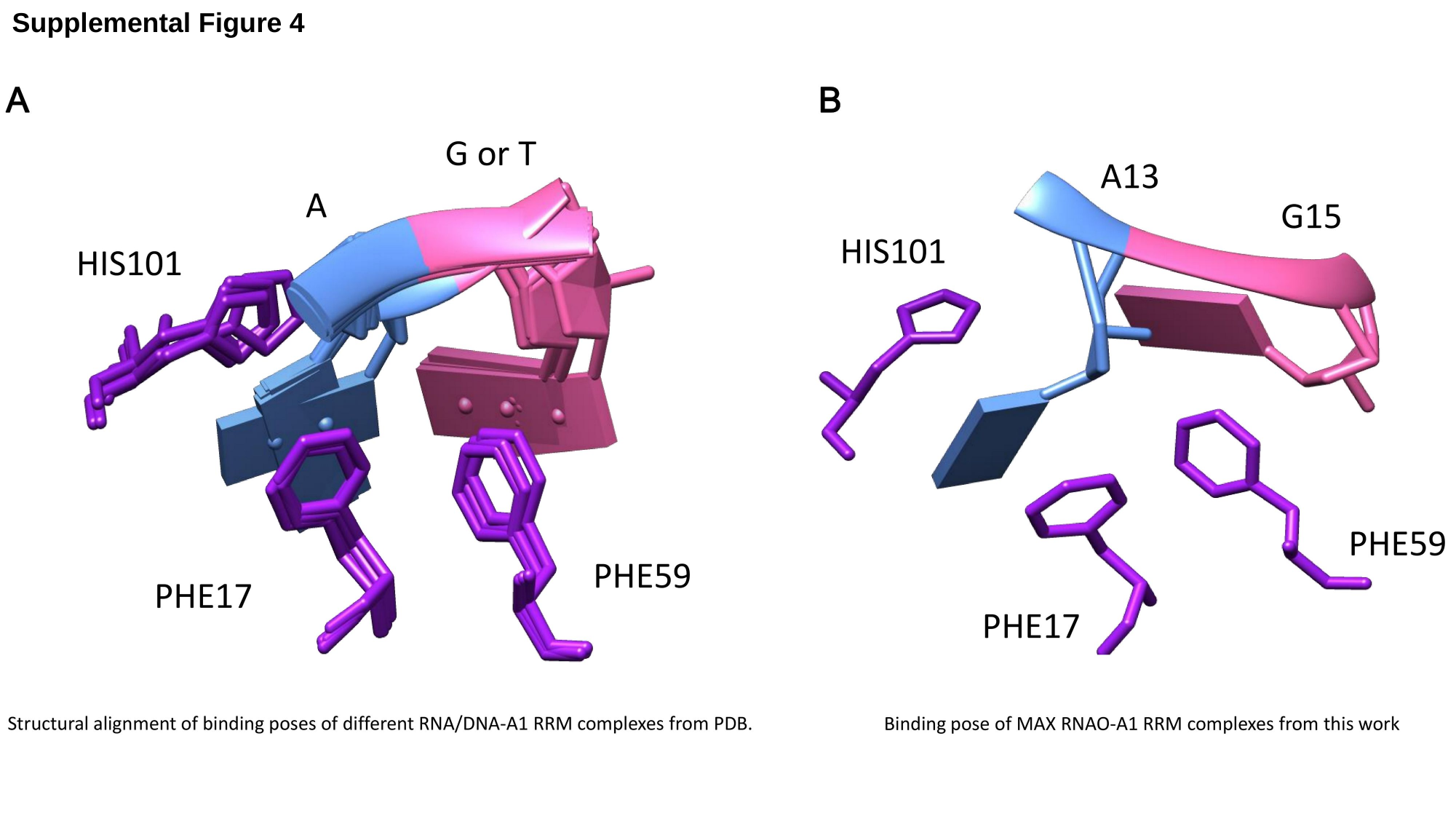

Supplemental Figure 4

## Slide 5
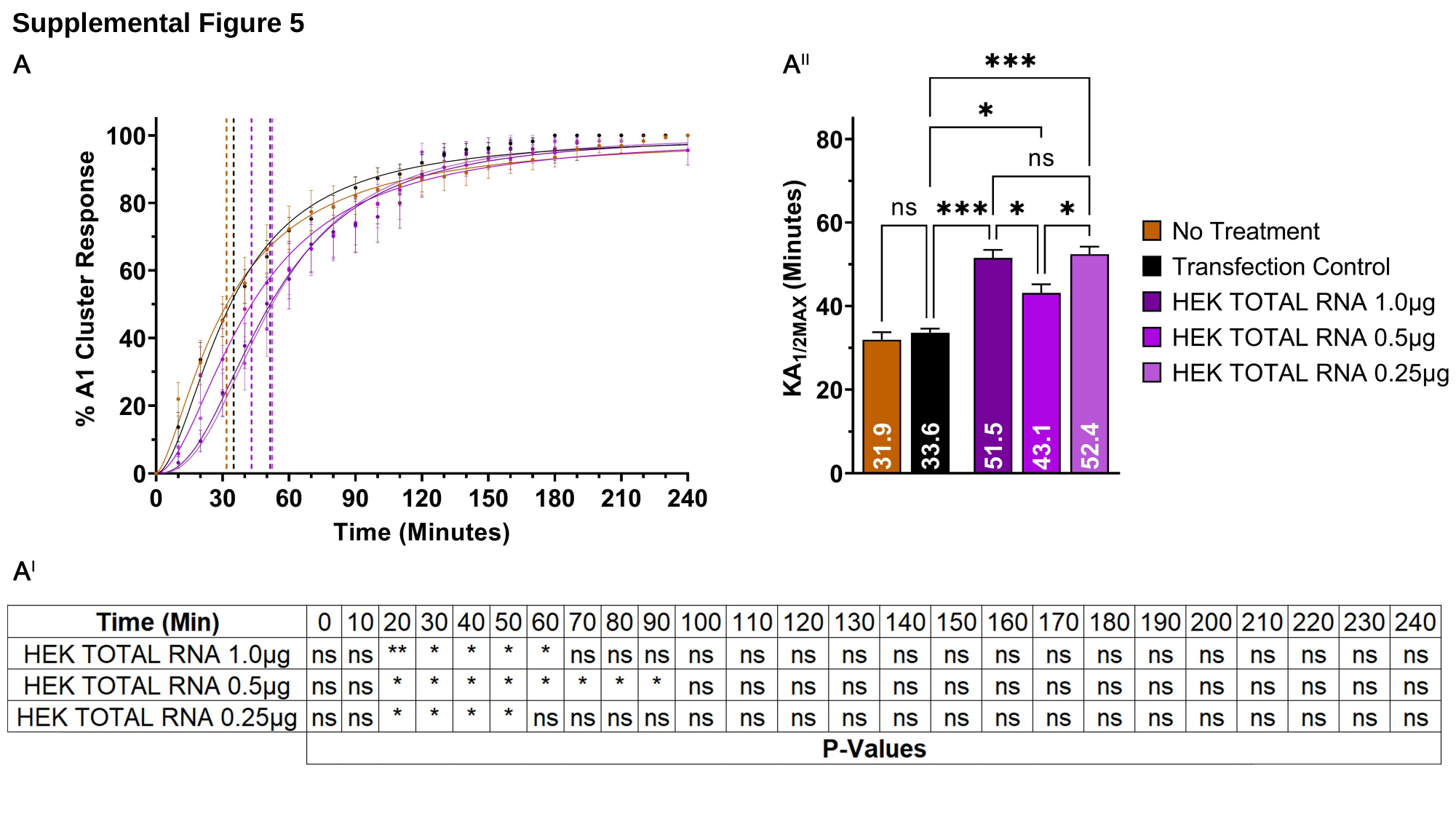

Supplemental Figure 5

## Slide 6
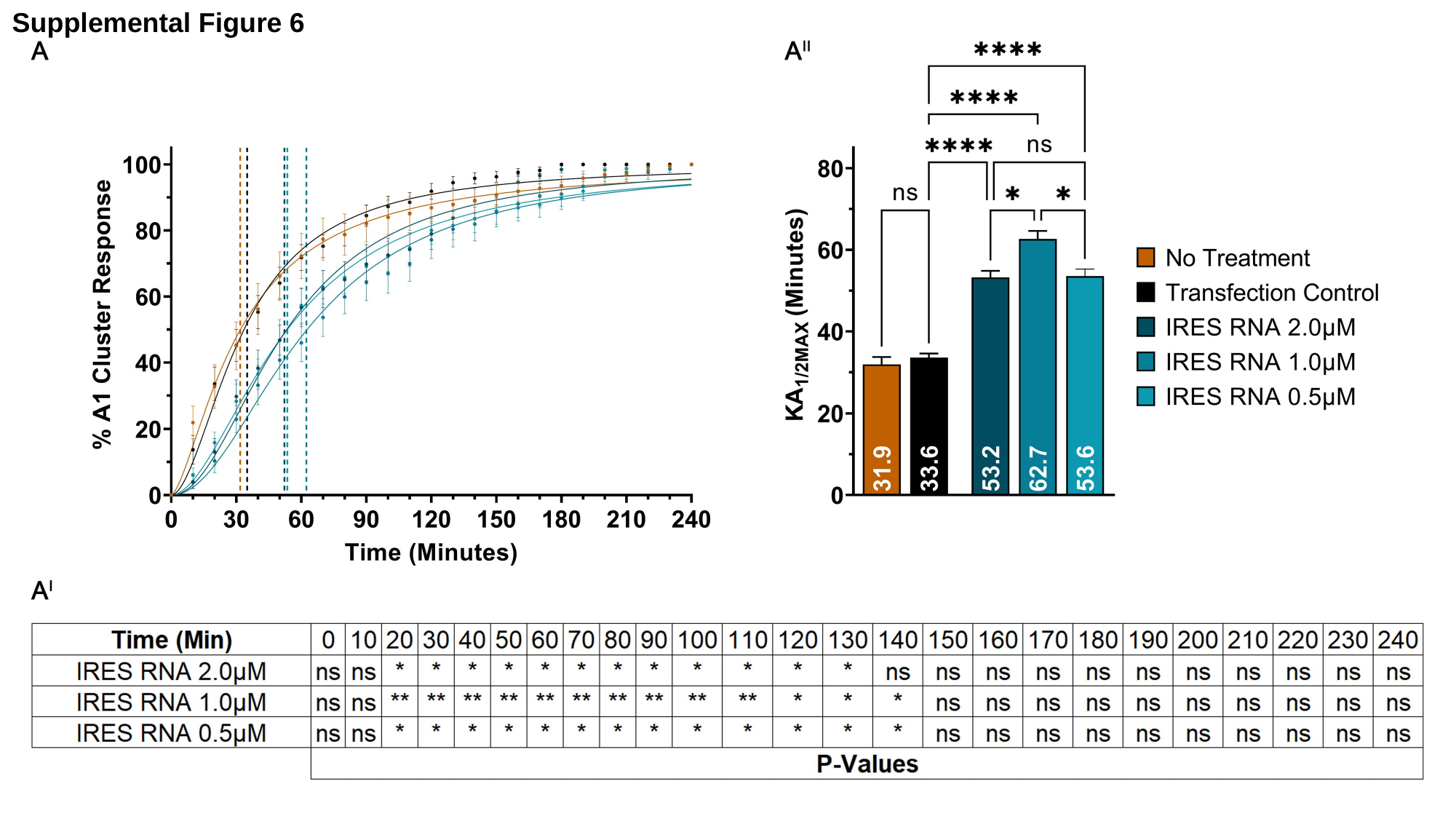

Supplemental Figure 6

## Slide 7
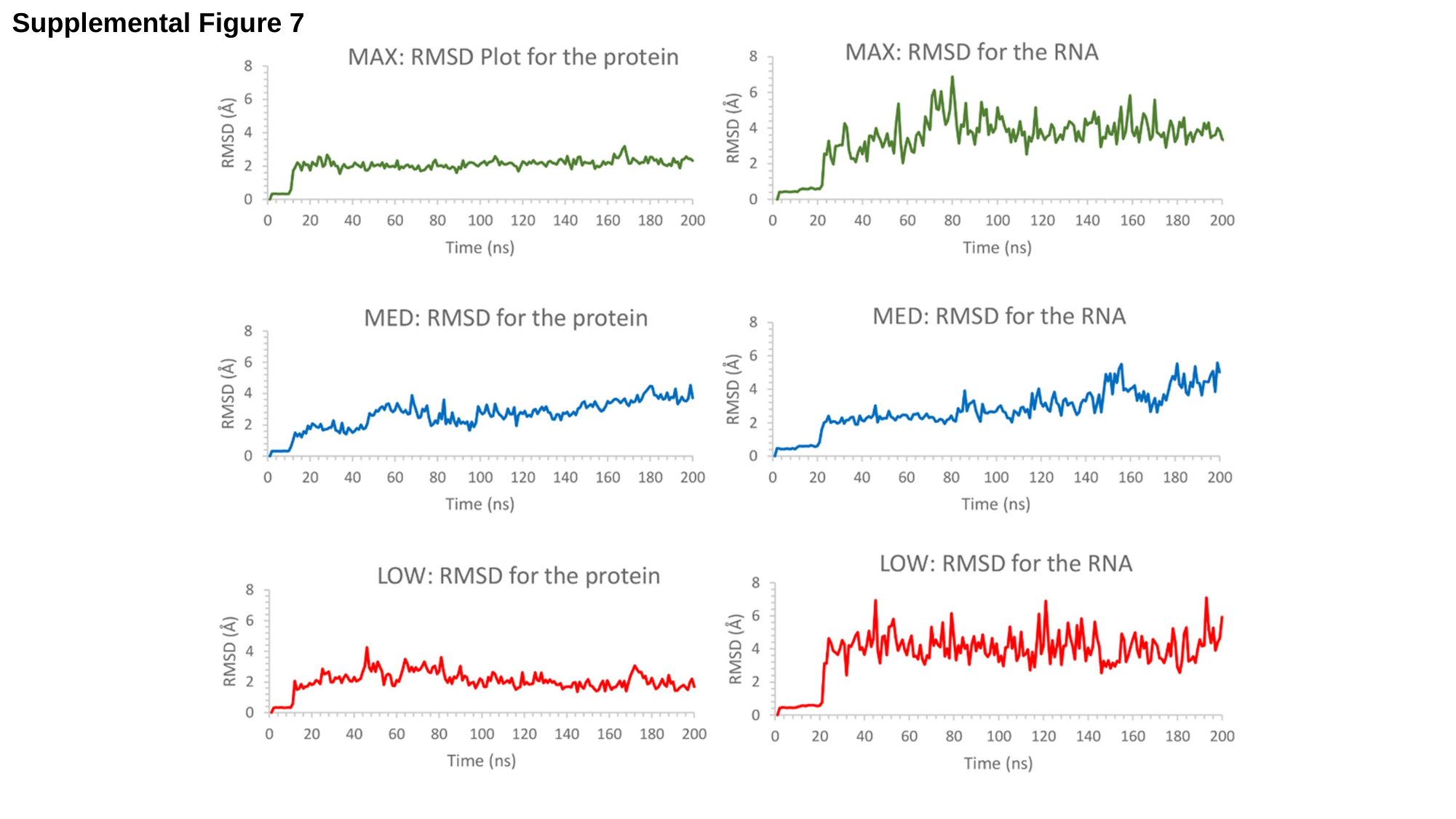

Supplemental Figure 7

## Slide 8
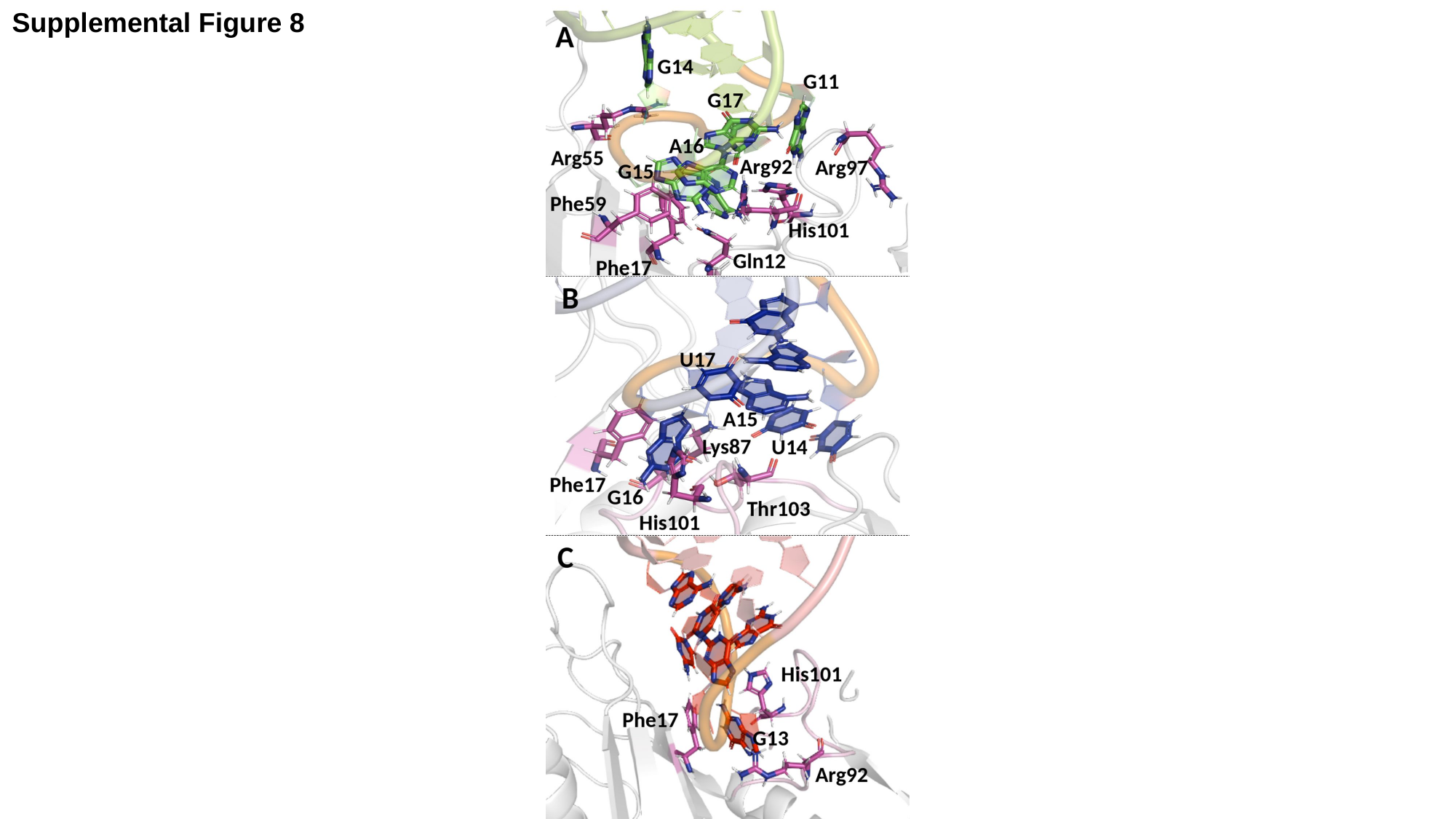

Supplemental Figure 8

## Slide 9
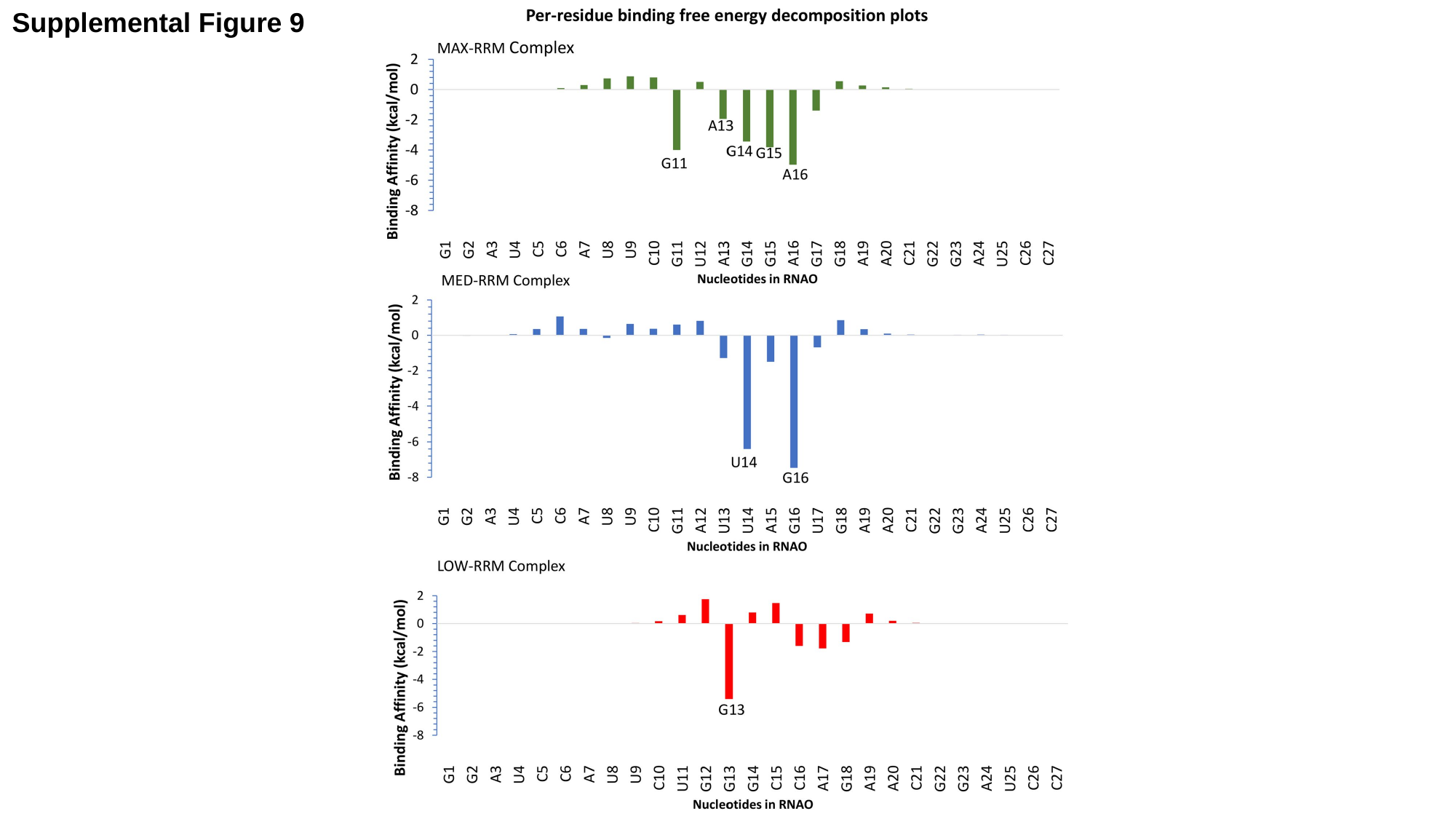

Supplemental Figure 9

## Slide 10
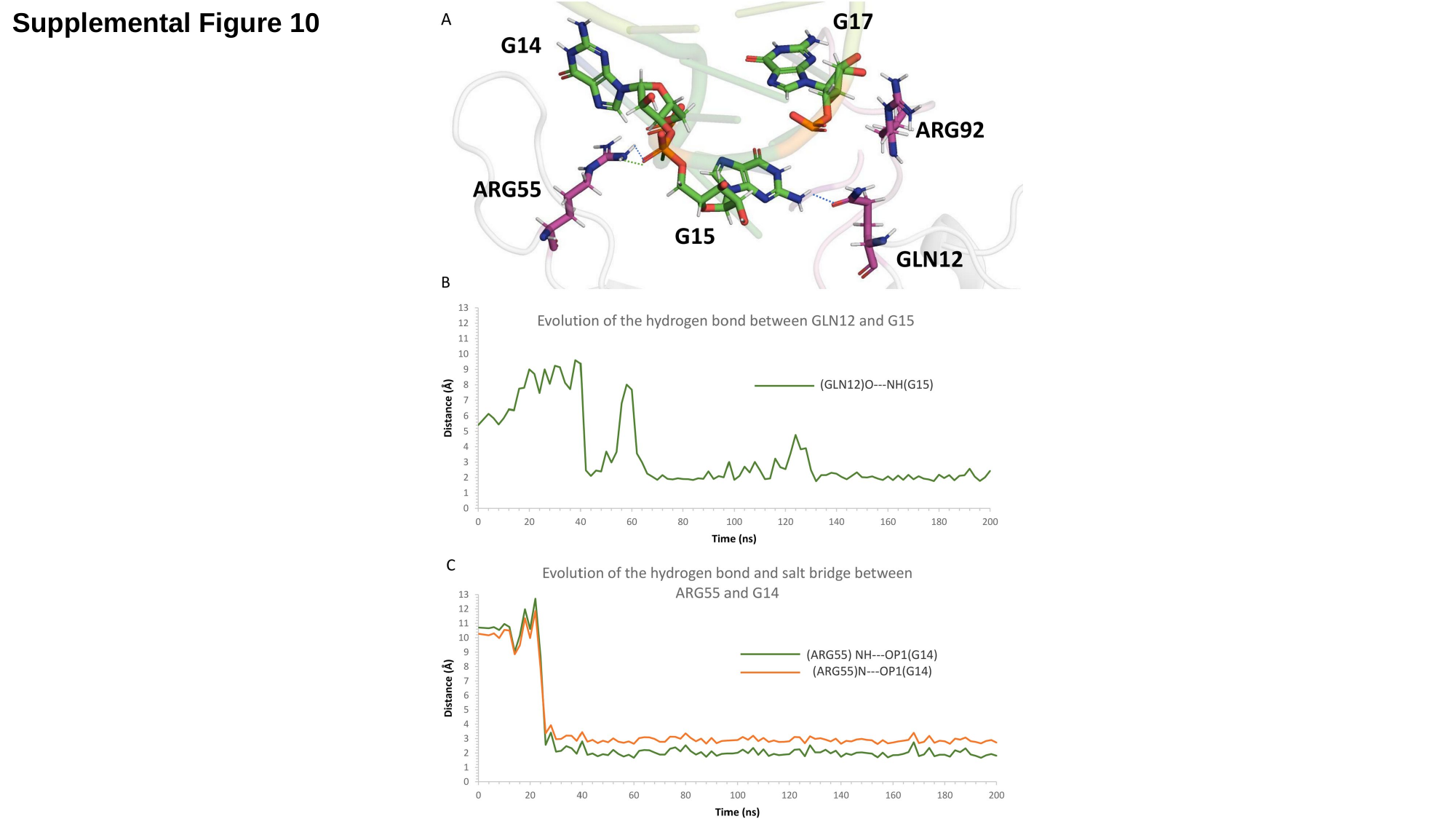

Supplemental Figure 10

## Slide 11
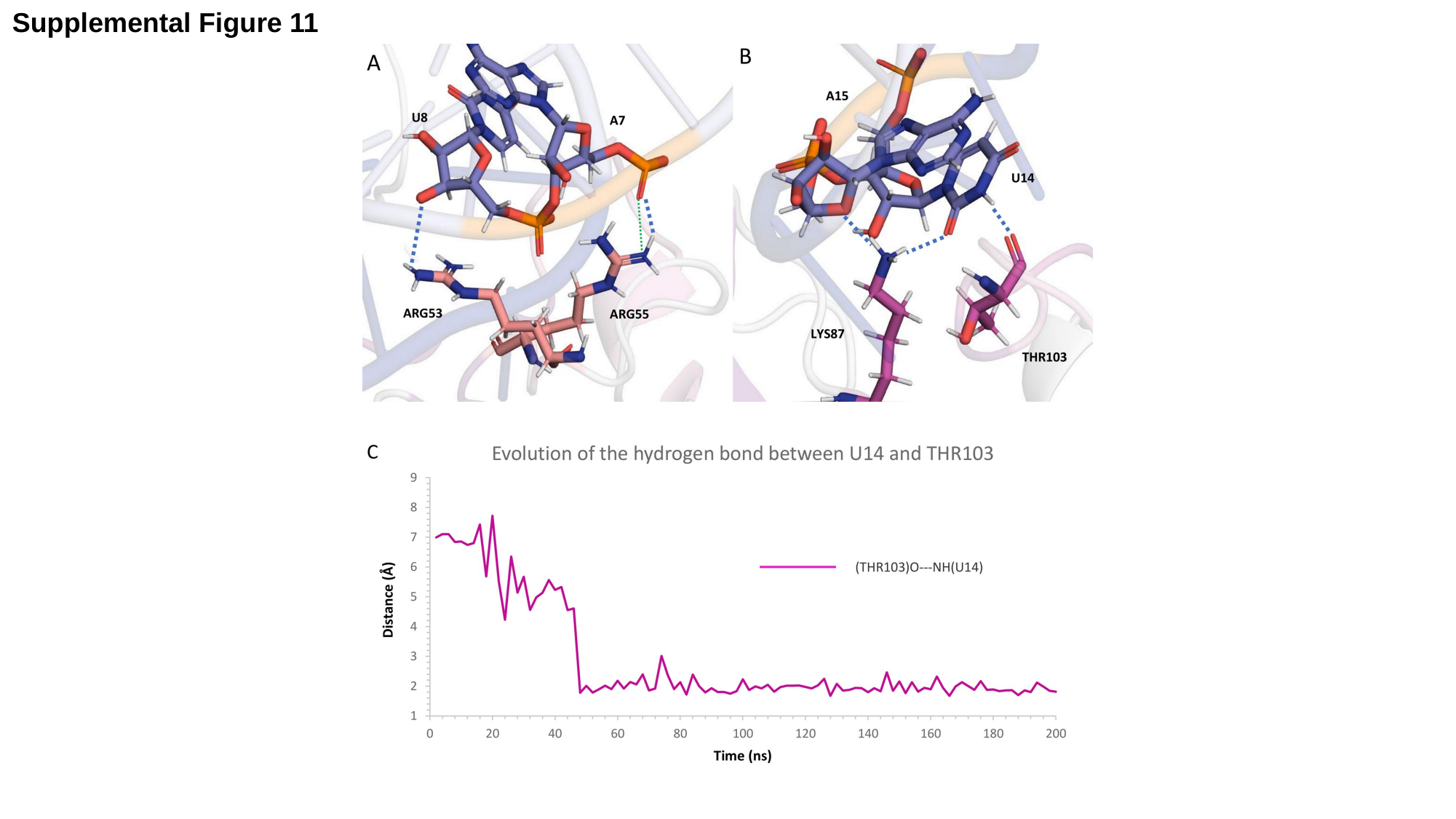

Supplemental Figure 11

## Slide 12
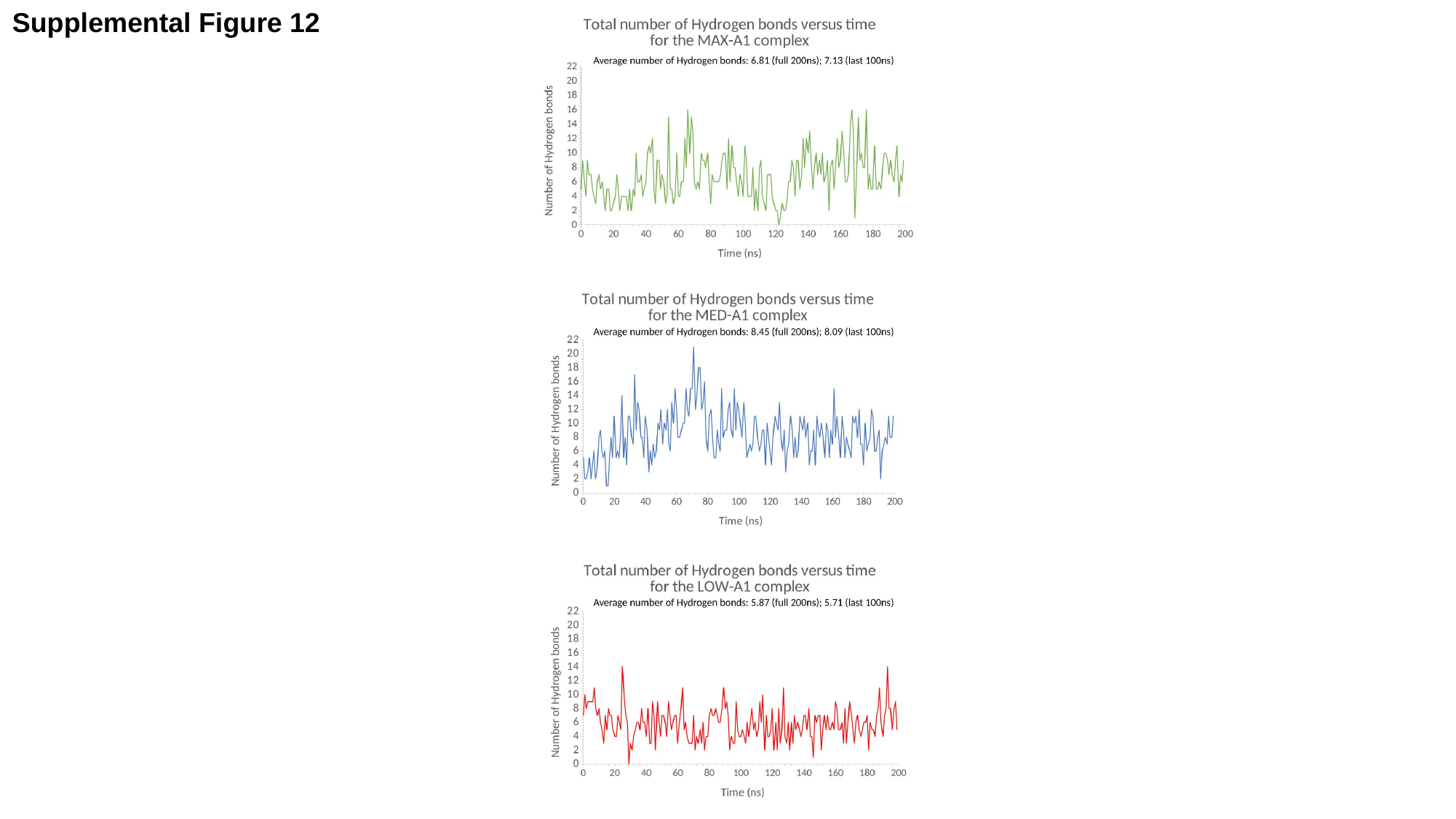

Supplemental Figure 12
