# Supplementary material for: Sequence- and structure-specific RNA oligonucleotide binding attenuates heterogeneous nuclear ribonucleoprotein A1 dysfunction
Source: Front Mol Biosci. 2023 Jun 22;10:1178439. doi: 10.3389/fmolb.2023.1178439 (PMC10325567; doi:10.3389/fmolb.2023.1178439)
Supplement: Supplementary file 5 [file Presentation2.pptx]

## Slide 1
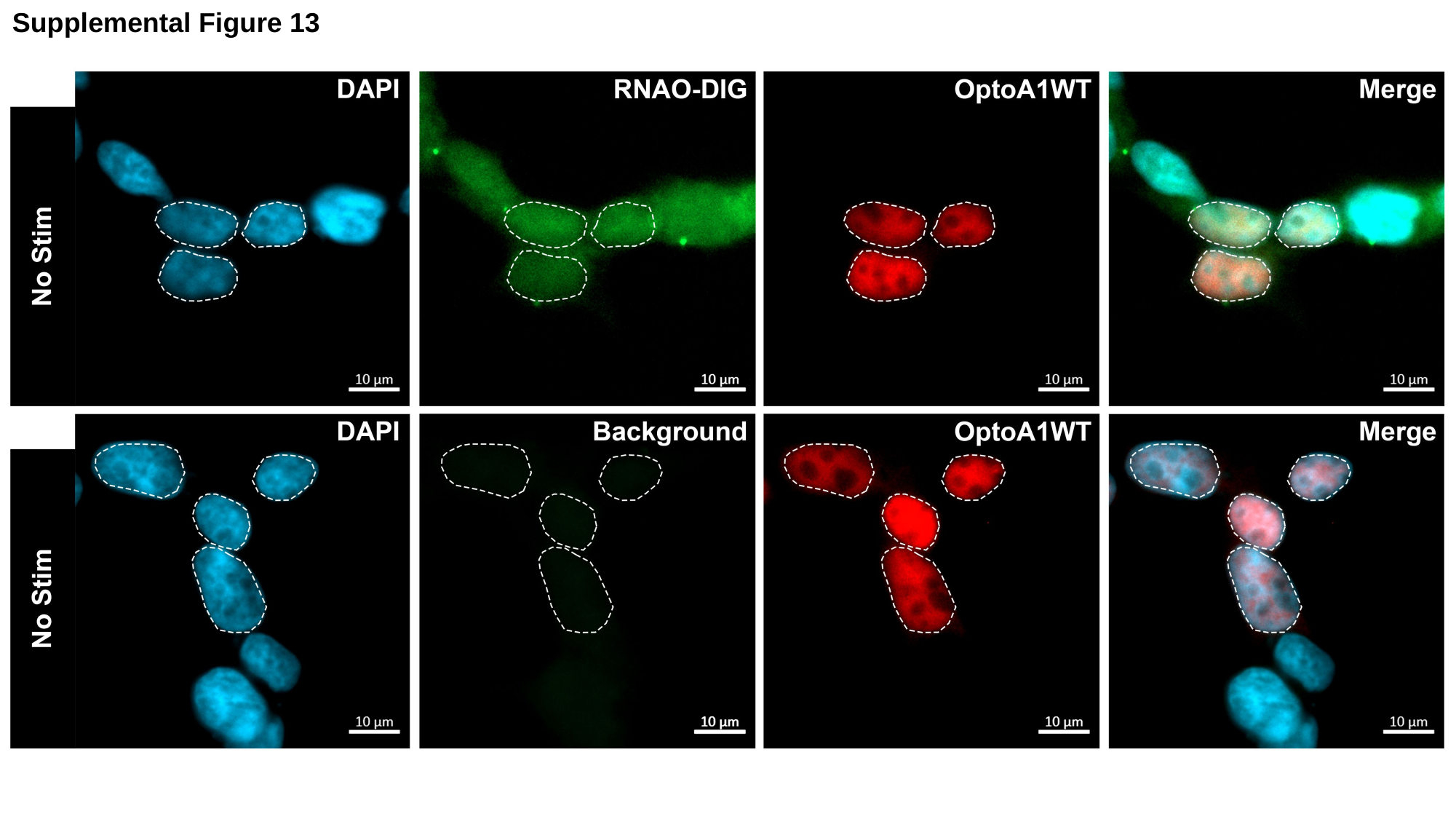

Supplemental Figure 13

## Slide 2
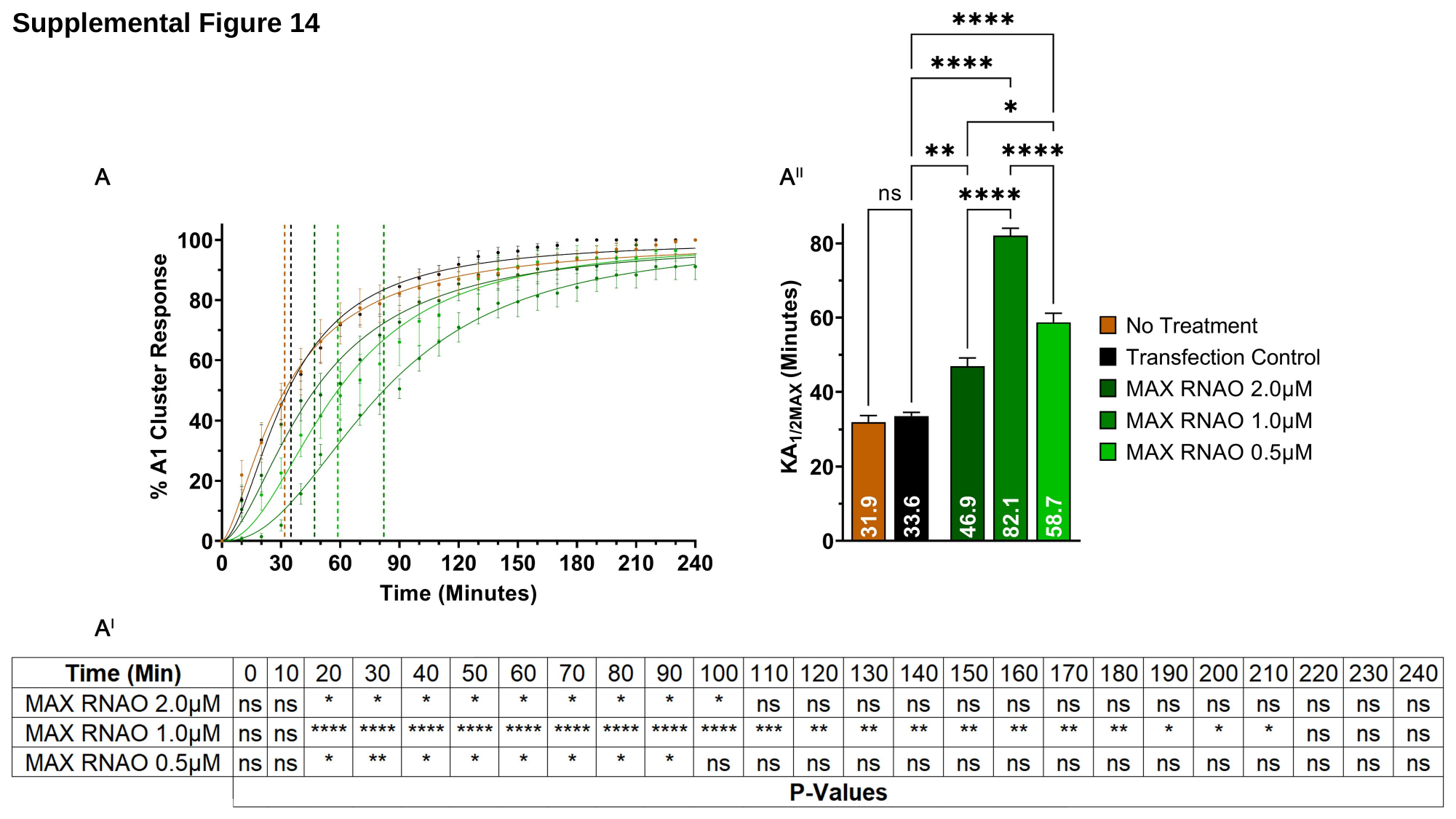

Supplemental Figure 14

## Slide 3
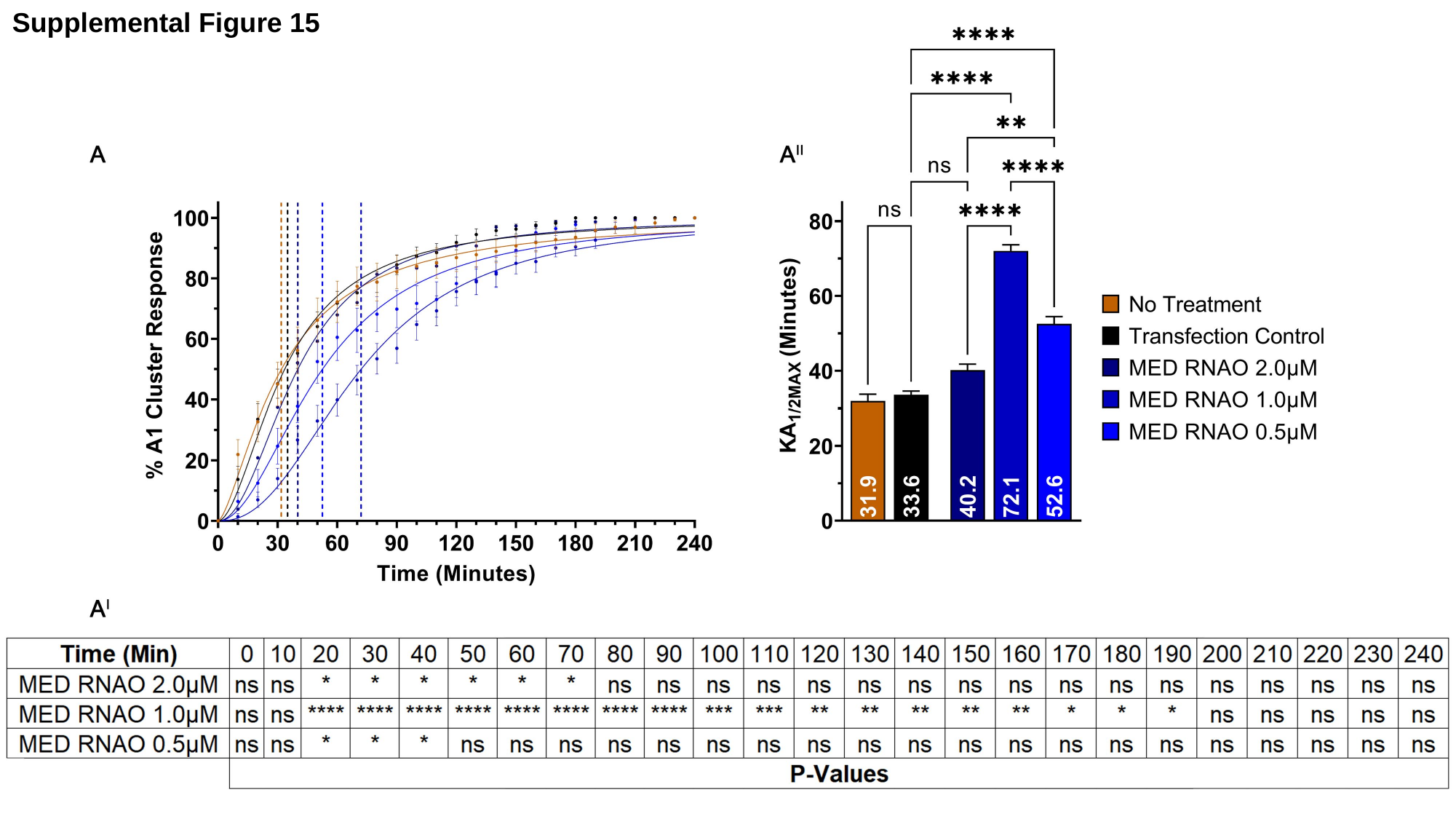

Supplemental Figure 15

## Slide 4
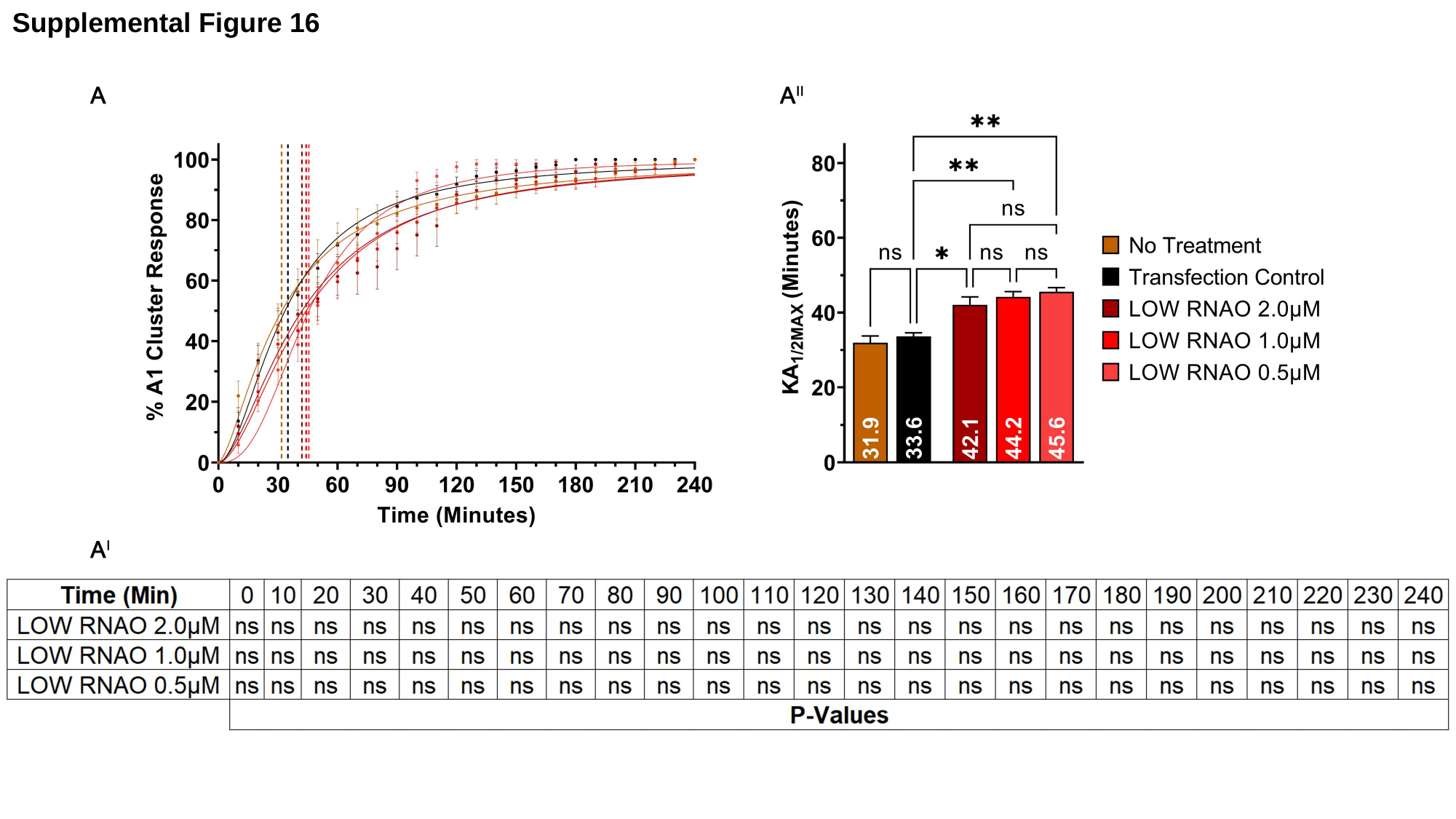

Supplemental Figure 16

## Slide 5
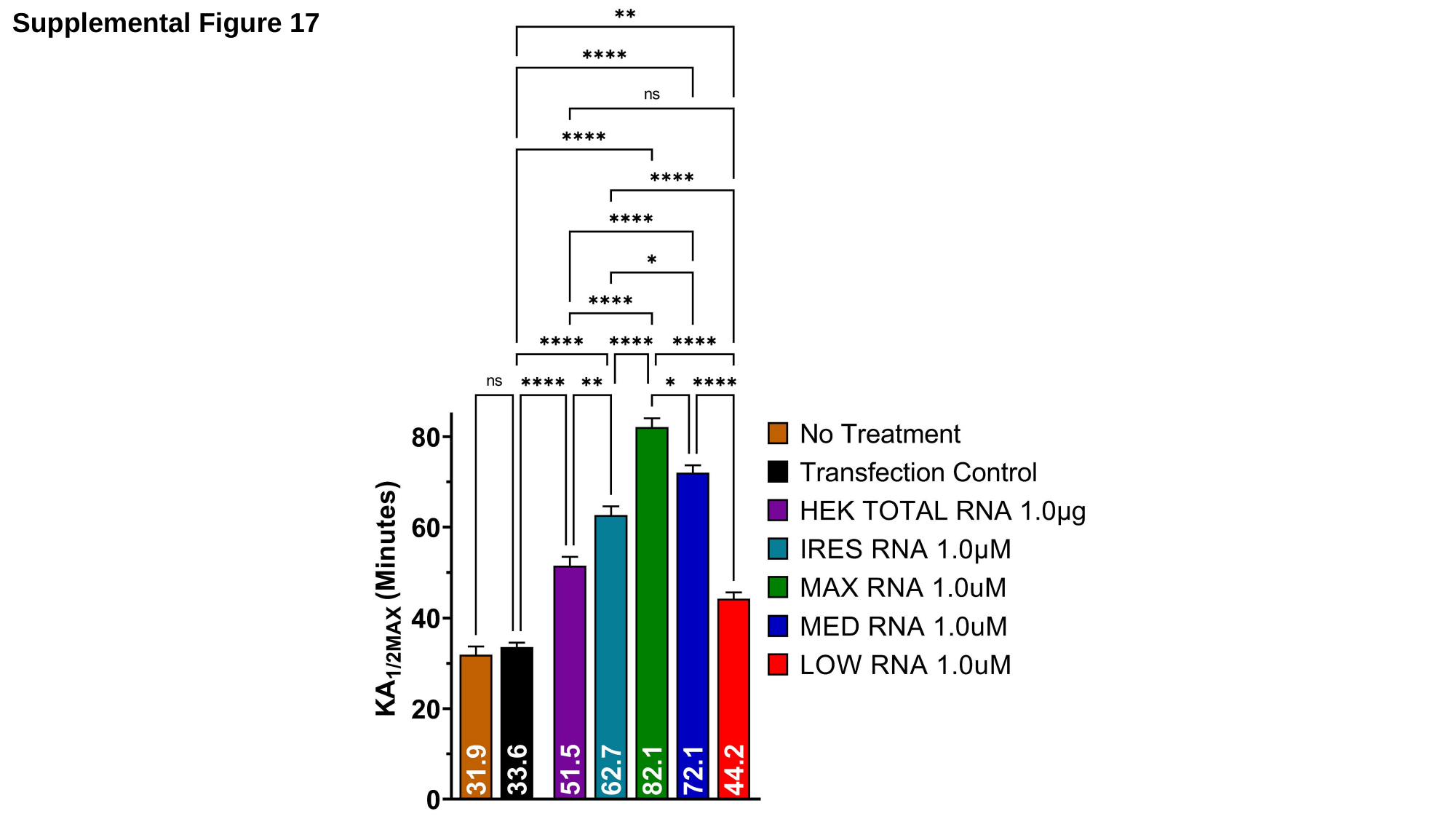

Supplemental Figure 17

## Slide 6
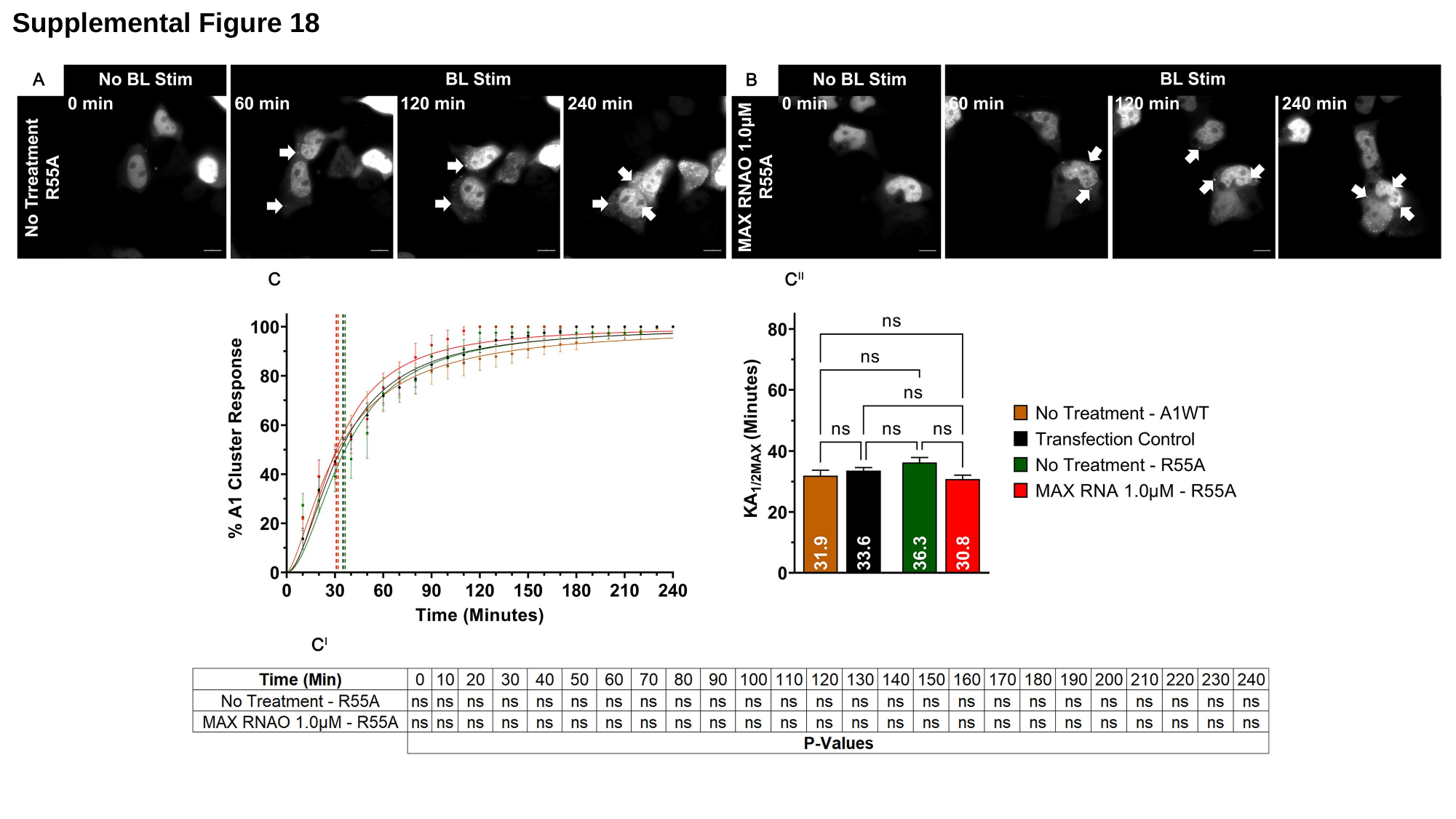

Supplemental Figure 18

## Slide 7
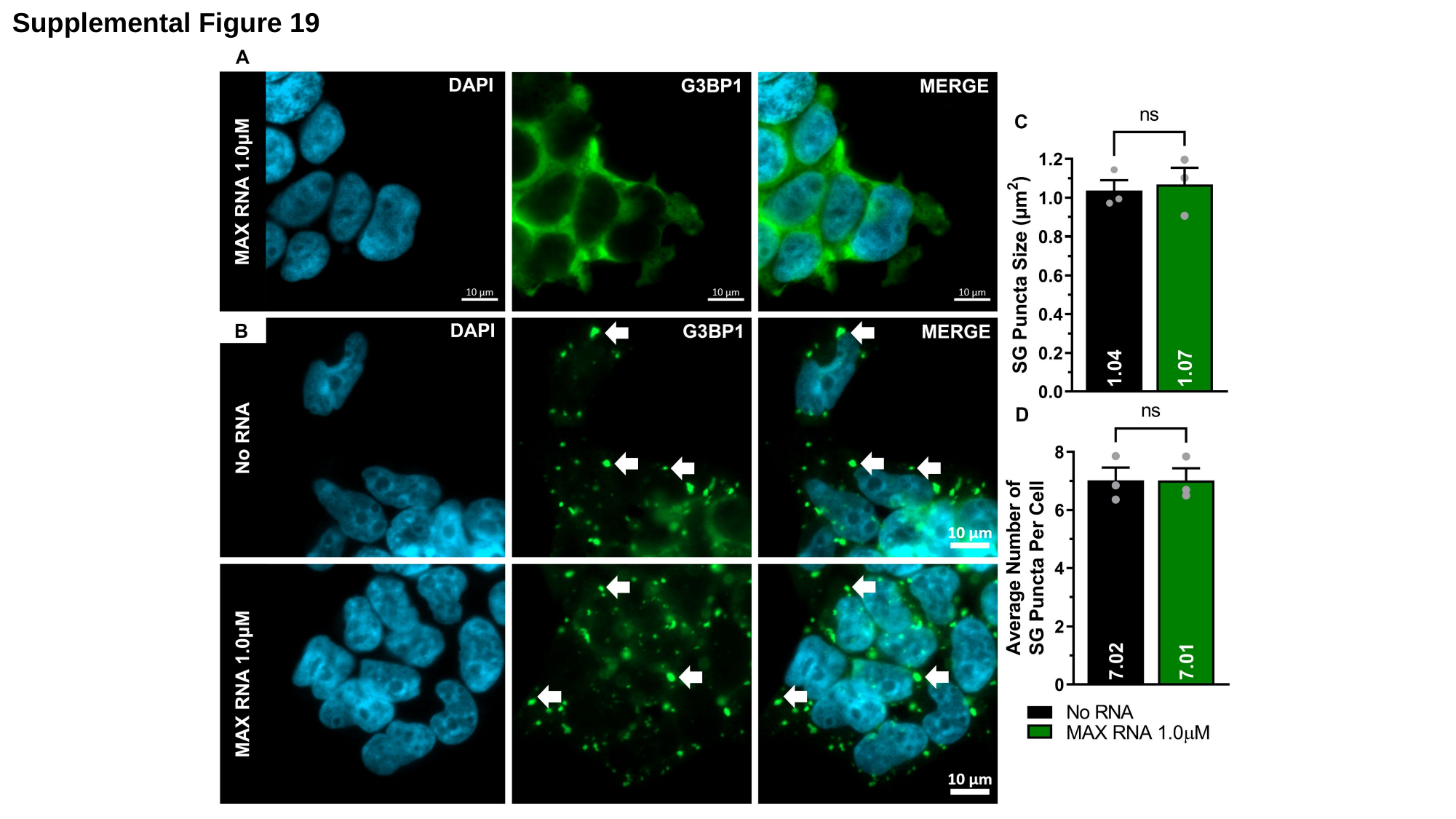

Supplemental Figure 19

## Slide 8
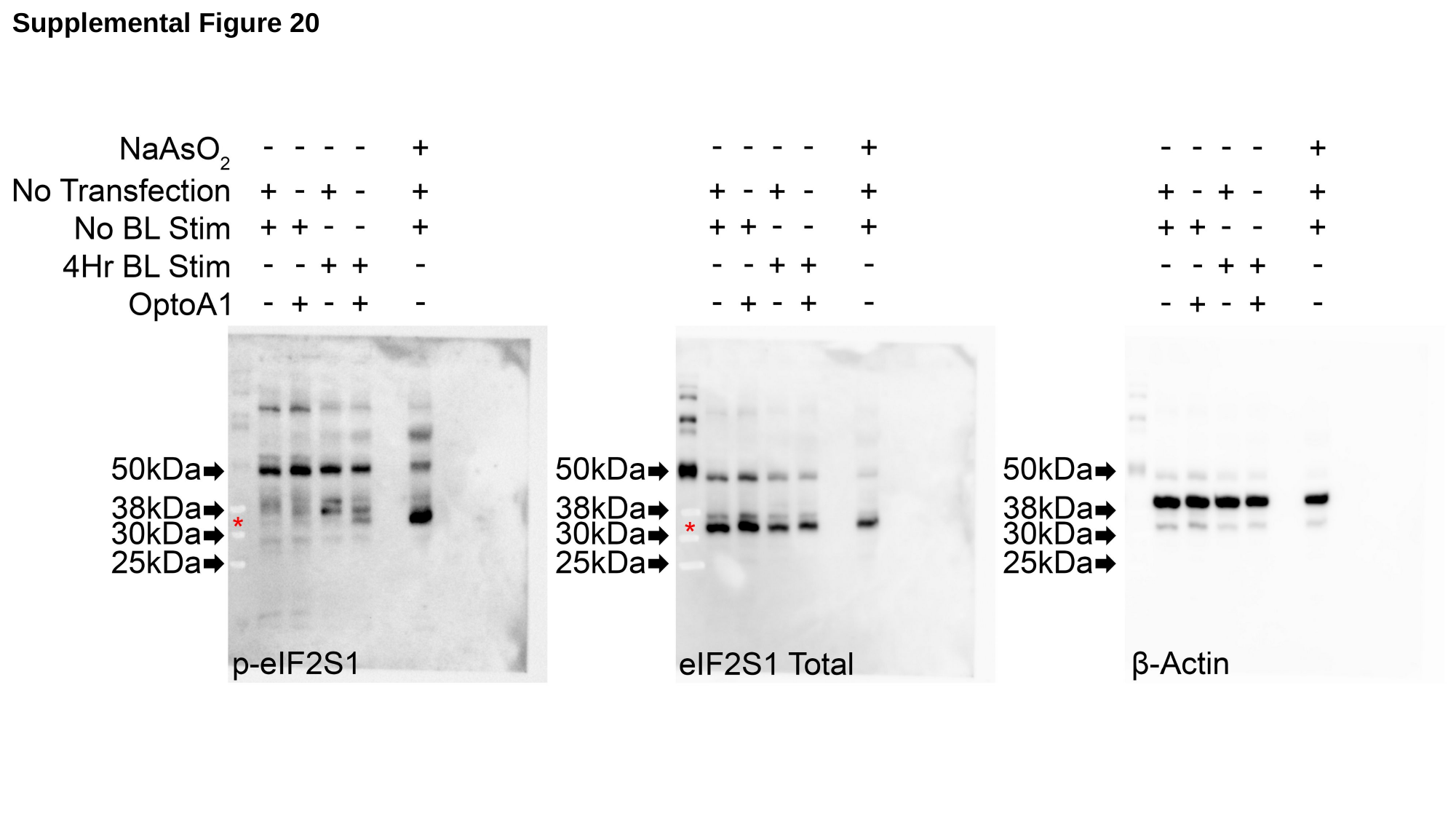

Supplemental Figure 20

## Slide 9
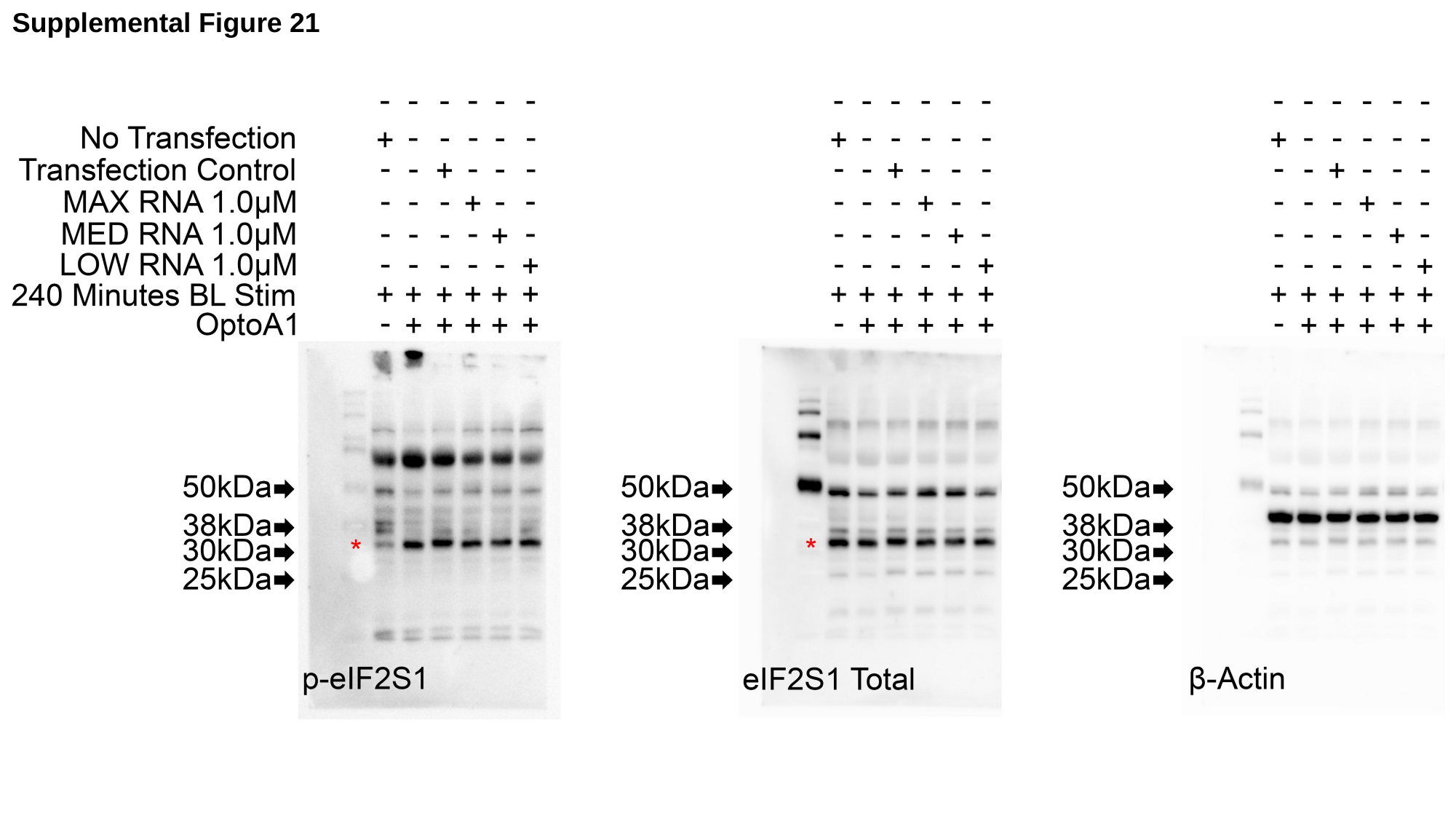

Supplemental Figure 21

## Slide 10
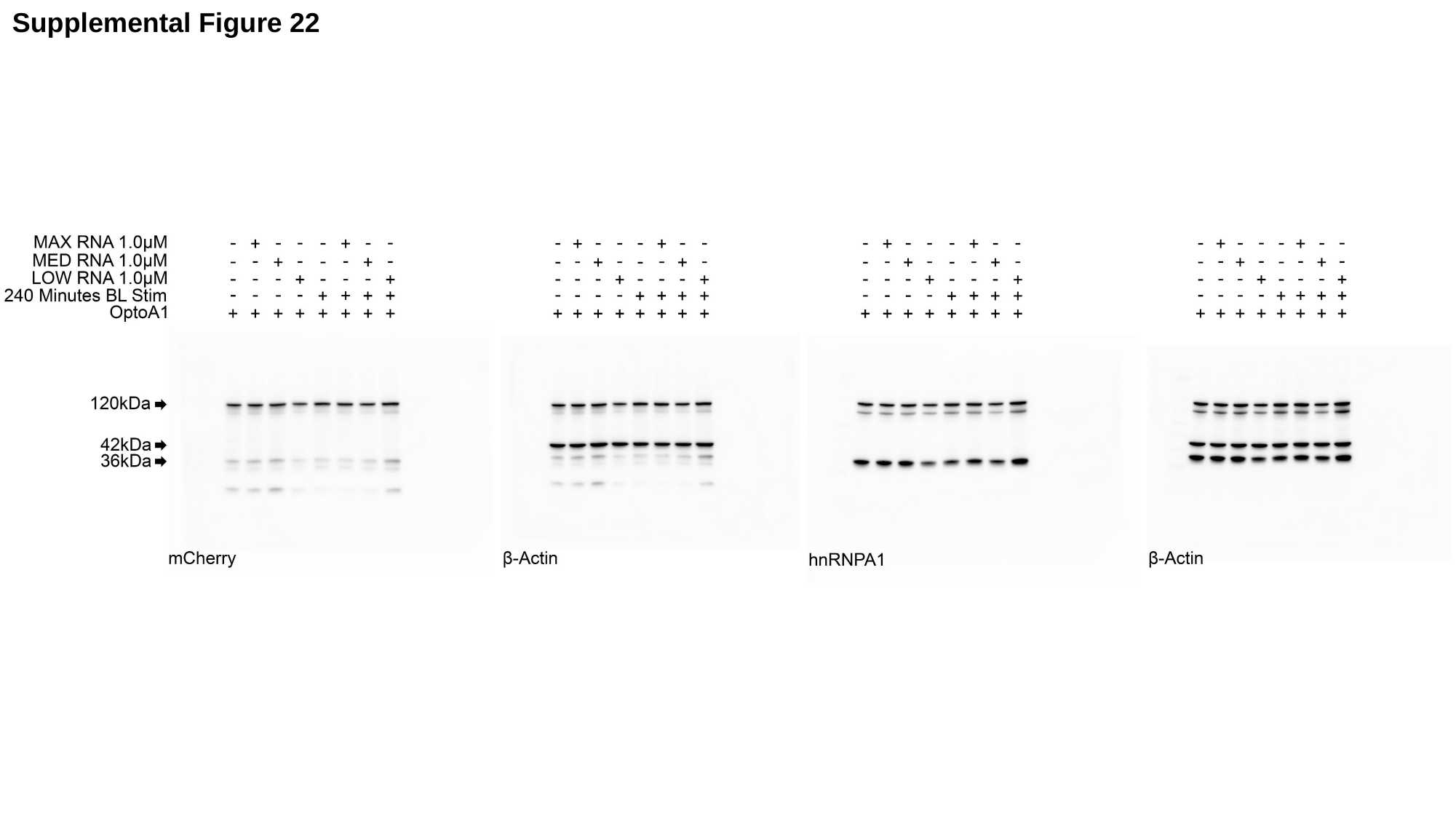

Supplemental Figure 22

## Slide 11
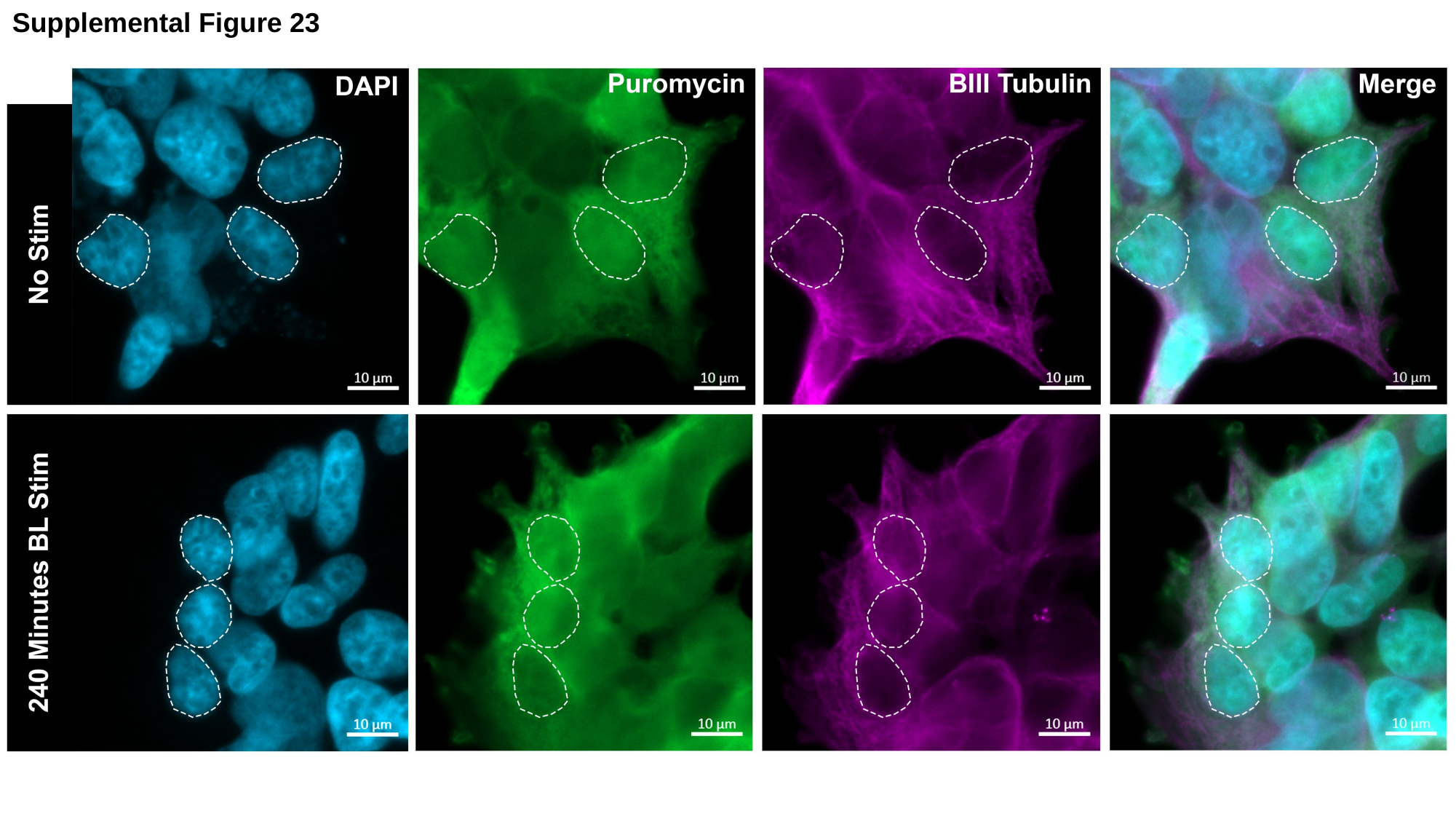

Supplemental Figure 23
