# Supplementary material for: Sequence- and structure-specific RNA oligonucleotide binding attenuates heterogeneous nuclear ribonucleoprotein A1 dysfunction
Source: Front Mol Biosci. 2023 Jun 22;10:1178439. doi: 10.3389/fmolb.2023.1178439 (PMC10325567; doi:10.3389/fmolb.2023.1178439)
Supplement: Supplementary file 9 [file DataSheet1.docx]

**SUPPLEMENTAL TABLE, FIGURE AND VIDEO LEGENDS**

**Supplemental Table 1:** DNA Primer and RNA Oligonucleotide Sequences

**Supplemental Figure 1: The docking poses and their respective scores for MAX RNAO against A1 RRM. (**A) All the complex poses obtained from docking are superposed, while showing all the top RNAO poses and only single RRM1 (grey ribbon). The docking scores of all the poses are shown in the embedded table. The complex coloured in green (complex 5) in this table is chosen for further analyses as it was the first top pose that exhibited native contacts against A1 RRM (B) when compared to the other poses. The orientation for complex 1, a false-positive hit, exhibiting non-specific binding of RNAO is also shown (C).

**Supplemental Figure 2: The docking poses and their respective scores for MED RNAO against A1 RRM. (**A) All the complex poses obtained from docking are superposed, while showing all the top RNAO poses and only single RRM1 (white ribbon). The docking scores of all the poses are shown in the embedded table. The complex coloured in green (complex 8) in this table is chosen for further analyses as it was the first top pose that exhibited native contacts against A1 RRM (B) when compared to the other poses. The orientation for complex 1, a false-positive hit, exhibiting non-specific binding of RNAO is also shown (C).

**Supplemental Figure 3: The docking poses and their respective scores for LOW RNAO against A1 RRM. (**A) All the complex poses obtained from docking are superposed, while showing all the top RNAO poses and only single RRM1 (white ribbon). The docking scores of all the poses are shown in the embedded table. The complex coloured in green (complex 12) in this table is chosen for further analyses as it was the first top pose that exhibited native contacts against A1 RRM (B) when compared to the other poses. The orientation for complex 1, a false-positive hit, exhibiting non-specific binding of RNAO is also shown (C).

**Supplemental Figure 4:** Stacking interactions in the MAX RNAO-RRM model compared to the previously reported structures in PDB. A) Superposed binding poses of oligonucleotide- A1 RRM complexes from crystal structures in PDB (4YOE[28], 1PGZ[55], 1PO6[55], 1U1K[56] and 2UP1[54]) revealed a pair of highly conserved aromatic stacking interactions rendered by an adenine nucleotide with PHE17 and HIS101, and a guanine with PHE59 in A1 RRM1. A, G, T = nucleotides. B) 3D binding poses of the modelled MAX-A1 RRM complex confirmed that this complex formed the two conserved stacking interactions through PHE17-A13-HIS101 network and the G15-PHE19 pair.

**Supplemental Figure 5:** Quantification of OptoA1cluster formation during 240-minute BL stimulation with the addition of **HEK TOTAL RNA** at three concentrations (1.0µg, 0.5µg or 0.25µg). A) Results are plotted as a percent maximum to the highest cluster response at 240 minutes for each RNA treatment, resulting in kinetics curves for association dynamics. No Treatment = no treatment with RNA; Transfection Control = cells only transfected with RNAiMAX. Dashed lines indicate KA_1/2Max_. A^I^) Tabular results of a two-way ANOVA, with a Bonferroni post-hoc test from the curves illustrated in A). A^II^) Bar graphs and one-way ANOVA, with a Tukey post-hoc test analysis of KA_1/2Max_ from the curves illustrated in A). Data shown are mean +/- S.E.M. for three biological replicates. *p<0.05; **p<0.01; ***p<0.001; ****p<0.0001; 95% Confidence Interval.

**Supplemental Figure 6:** Quantification of OptoA1 cluster formation during 240-minute BL stimulation with the addition of **IRES RNA** at three separate concentrations (2.0µM, 1.0µM or 0.5µM). A) Results are plotted as a percent maximum to the highest cluster response at 240 minutes for each RNA treatment, resulting in kinetics curves for association dynamics. No Treatment = no treatment with RNA; Transfection Control = cells only transfected with RNAiMAX. Dashed lines indicate KA_1/2Max_. A^I^) Tabular results of a two-way ANOVA, with a Bonferroni post-hoc test from the curves illustrated in A). A^II^) Bar graphs and one-way ANOVA, with a Tukey post-hoc test analysis of KA_1/2Max_ from the curves illustrated in A). Data shown are mean +/- S.E.M. for three biological replicates. *p<0.05; **p<0.01; ***p<0.001; ****p<0.0001; 95% Confidence Interval.

**Supplemental Figure 7:** Evolution of RMSD of the RNAO-A1 complexes. The stability of the complexes during MD simulation was assessed by plotting the evolution of backbone RMSD of A1 RRMs (on the left-side panel) and all the atoms of RNAOs (on the right-side panel). As seen in the plots on the left, the protein has much lower RMSD values than those of RNAOs (right) for all three complexes. RNAOs underwent more conformation changes to adapt to its binding with A1 RRMs that was stable during simulation.

**Supplemental Figure 8:** Overview of the interactions contributing significantly to the stabilization of each RNAO molecule in the RRM1 binding pocket. (A) As summarized in Figure 3 and Supplemental Figure 12, the MAX RNAO has the greatest number of pi-pi stacking as well as electrostatic interactions that are responsible for its highest binding-free energy value. This is attributed to the two AG motifs available within the apical loop for native RNA-RRM1 contacts. **(**B) The MED RNAO, although having fewer interactions than MAX, is still able to form some pi-pi contacts as it still has an AG motif in the RNP site, as well as some notable hydrogen bonds. (C) Since the LOW RNAO lacks an AG motif, it is not able to form the signature interactions as noted for MAX or MED RNAOs. However, it is still able to bind using nonspecific contacts from the rest of the RNA. This is also apparent with the apical loop residues forming self-stacking interactions and not being available for extensive interactions with RNPs other than G13.

**Supplemental Figure 9:** Energy decomposition plots show the key nucleotides from RNAOs that interact with A1 RRM. The per-nucleotide energy decomposition analyses showed that most of the seven-nucleotide long apical loop (11-17 nucleotide positions) in MAX RNAO contributed to the free energy of its complex with RRMs (top-panel). Only adenines and guanines contributed to the interactions of MAX RNAO with RRMs. Wherein, the MED RNAO-RRM complex (in the middle panel), only U14 and G16 from the apical loop of RNAO contributed significantly to the complex stability. In the bottom-panel, only G13 nucleotide from the LOW RNAO made a significant contribution to its complex with RRMs; other nucleotides exhibited a marginal role in this process.

**Supplemental Figure 10:** Illustration of the prominent electrostatic interactions in the MAX RNAO-A1 complex. A) A 3D representation of key salt-bridge and hydrogen bond interactions between nucleotides such as G14, G15 and G17 against ARG55, GLN12 and ARG92, respectively. The selected amino acids and nucleotides are shown as stick representations as the other segments of the binding pose are shown as cartoon representations in the background. Evolution of distances between the side-chain carbonyl group of GLN12 and purine ring of G15 (B) confirmed that these interactions formed after ~60 ns and remained mostly stable until the end of simulation. Similarly, the distance evolution between the side-chain amino groups of ARG55 and phosphate group of G15 (C) described that this pair established a salt-bridge (N-OP1 shown in orange) and a hydrogen bond (NH-OP1 shown in green) after 20 ns and maintained them throughout the course of MD simulation.

**Supplemental Figure 11:** Illustration of the prominent electrostatic interactions in the MED RNAO-A1 complex. 3D representations of key salt-bridge and hydrogen bond interactions between key nucleotides from MED RNAO and amino acids from RRMs are shown in A-B. ARG53 and ARG55 formed dynamic salt-bridge contacts with A7 and U8 nucleotides from MED RNAO (A); while the side-chain amino group of LYS87 and backbone of THR103 made hydrogen bond contacts with A15 and U14, respectively, (B). The evolution of distance between the backbone carbonyl oxygen atom in THR103 and pyrimidine ring of U14 confirmed the stability of their hydrogen bond during MD simulation.

**Supplemental Figure 12:** The total number of hydrogen bonds calculated for the MAX, MED, and LOW RNAO-A1 complexes. While MAX and MED RNAOs display similar number of hydrogen bonds, LOW RNAO has reduced capacity for them.

**Supplemental Figure 13: RNAOs ubiquitously transfect into HEK293T cells, regardless of OptoA1 transfection.** Representative images of HEK293T cells transfected with OptoA1, and MAX RNAO tagged with digoxigenin (DIG). Top Panel: MAX RNAO DIG was detected using primary mouse monoclonal anti-digoxigenin and secondary mouse monoclonal Alexa Fluor 488. Bottom Panel: For negative control, background only cells, cells were transfected with MAX RNAO DIG, but were only incubated with secondary mouse monoclonal Alexa Fluor 488. Dashed lines outline cellular nuclei in OptoA1 transfected cells. Scale bars = 10 µm.

**Supplemental Figure 14:** Quantification of OptoA1 cluster formation during 240-minute BL stimulation with the addition of **MAX RNAO** at three concentrations (2.0µM, 1.0µM or 0.5µM). A) Results are plotted as a percent maximum to the highest cluster response at 240 minutes for each RNA treatment, resulting in kinetics curves for association dynamics. No Treatment = no treatment with RNA; Transfection Control = cells only transfected with RNAiMAX. Dashed lines indicate KA_1/2Max_. A^I^) Tabular results of a two-way ANOVA, with a Bonferroni post-hoc test from the curves illustrated in A). A^II^) Bar graphs and one-way ANOVA, with a Tukey post-hoc test analysis of KA_1/2Max_ from the curves illustrated in A). Data shown are mean +/- S.E.M. for three biological replicates. *p<0.05; **p<0.01; ***p<0.001; ****p<0.0001; 95% Confidence Interval.

**Supplemental Figure 15:** Quantification of OptoA1 cluster formation during 240-minute BL stimulation with the addition of **MED RNAO** at three concentrations (2.0µM, 1.0µM or 0.5µM). A) Results are plotted as a percent maximum to the highest cluster response at 240 minutes for each RNA treatment, resulting in kinetics curves for association dynamics. No Treatment = no treatment with RNA; Transfection Control = cells only transfected with RNAiMAX. Dashed lines indicate KA_1/2Max_. A^I^) Tabular results of a two-way ANOVA, with a Bonferroni post-hoc test from the curves illustrated in A). A^II^) Bar graphs and one-way ANOVA, with a Tukey post-hoc test analysis of KA_1/2Max_ from the curves illustrated in A). Data shown are mean +/- S.E.M. for three biological replicates. *p<0.05; **p<0.01; ***p<0.001; ****p<0.0001; 95% Confidence Interval.

**Supplemental Figure 16:** Quantification of OptoA1 cluster formation during 240-minute BL stimulation with the addition of **LOW RNAO** at three concentrations (2.0µM, 1.0µM or 0.5µM). A) Results are plotted as a percent maximum to the highest cluster response at 240 minutes for each RNA treatment, resulting in kinetics curves for association dynamics. No Treatment = no treatment with RNA; Transfection Control = cells only transfected with RNAiMAX. Dashed lines indicate KA_1/2Max_. A^I^) Tabular results of a two-way ANOVA, with a Bonferroni post-hoc test from the curves illustrated in A). A^II^) Bar graphs and one-way ANOVA, with a Tukey post-hoc test analysis of KA_1/2Max_ from the curves illustrated in A). Data shown are mean +/- S.E.M. for three biological replicates. *p<0.05; **p<0.01; ***p<0.001; ****p<0.0001; 95% Confidence Interval.

**Supplemental Figure 17:** Comparison of OptoA1 KA_1/2MAX_ during 240-minute BL stimulation with the addition of **HEK TOTAL RNA**, **IRES RNA**, **MAX RNAO**, **MED RNAO**, or **LOW RNAO**. Bar graphs and one-way ANOVA, with a Tukey post-hoc test analysis of KA_1/2Max_ from the curves illustrated in Supplemental Figures 5, 6, 14, 15 and 16. Data shown are mean +/- S.E.M. for three biological replicates. *p<0.05; **p<0.01; ***p<0.001; ****p<0.0001; 95% Confidence Interval.

**Supplemental Figure 18: Mutation of RRM1 inhibits RNAO binding.** Representative images of blue light (BL) stimulated R55A OptoA1 mutant cells with either A) No Treatment or B) treated with 1.0µM MAX RNAO. C) Quantification of A1 cluster formation during a 240-minute BL stimulation protocol with No Treatment (**Green**) or MAX RNAO (**Red**). Results are plotted as a percent maximum to the highest cluster response at 240 minutes for each RNA treatment, resulting in a kinetics curve for association dynamics. No Treatment OptoA1 wild-type cells (**Brown**); Transfection Control OptoA1 wild-type cell (**Black**) = cells only transfected with RNAiMAX. Dashed lines indicate KA_1/2Max_. C^I^) Tabular results of a two-way ANOVA, with a Bonferroni post-hoc test from the curves illustrated in C). C^II^) Bar graphs and one-way ANOVA, with a Tukey post-hoc test analysis of KA_1/2Max_ from the curves illustrated in C). Data shown are mean +/- S.E.M. for three biological replicates. Arrows indicate the formation of OptoA1 clusters. Scale bars = 10 µm. *p<0.05; **p<0.01; ***p<0.001; ****p<0.0001; 95% Confidence Interval.

**Supplemental Figure 19:** RNAOs do not affect SG formation nor SG puncta characteristics. A) Representative images of untreated HEK293T cells transfected with 1.0µM MAX RNA. B) Representative images of NaAsO_2_ treated HEK293T cells transfected with and without 1.0µM MAX RNA. Arrows indicate the formation of SG puncta. Scale bars = 10 µm. Quantification of C) average SG puncta size and D) average number of SG puncta per cell, from one biological experiment. All results analyzed using a one-way ANOVA, with a Tukey post-hoc test. Data shown are mean +/- S.E.M.

**Supplemental Figure 20:** Full, unedited Western immunoblot images from Figure 7A. Red star indicates the bands corresponding to phospho-eIF2S1 and eIF2S1 Total (36kDa). To note, the Western immunoblots were probed sequentially: phospho-eIF2S1 🡪 eIF2S1 Total 🡪 β-Actin.

**Supplemental Figure 21:** Full, unedited Western immunoblot images from Figure 7B. Red star indicates the bands corresponding to phospho-eIF2S1 and eIF2S1 (36kDa). To note, the Western immunoblots were probed sequentially: phospho-eIF2S1 🡪 eIF2S1 Total 🡪 β-Actin.

**Supplemental Figure 22:** Full, unedited Western immunoblot images from Figure 7C. Bands corresponding to OptoA1 – 120kDa; bands corresponding to endogenous A1 – 36kDa. To note, the Western immunoblots were probed sequentially: mCherry 🡪 β-Actin; hnRNPA1 (4B10) 🡪 β-Actin.

**Supplemental Figure 23:** MAX RNAO treatment alone does not perturb protein translation. Representative images of HEK293T cells transfected with MAX RNAO, without OptoA1 transfection, probed for puromycin incorporation and treated with (Bottom Panel) and without (Top Panel) BL stimulation for 240 minutes. Dashed lines outline cellular nuclei. Scale bars = 10 µm.

**Supplemental Video 1:** No treatment, OptoA1 cluster formation over the time-course of a BL experimental paradigm.

**Supplemental Video 2:** Transfection control, OptoA1 cluster formation over the time-course of a BL experimental paradigm.

**Supplemental Video 3:** HEK TOTAL RNA treated, OptoA1 cluster formation over the time-course of a BL experimental paradigm.

**Supplemental Video 4:** IRES RNA treated, OptoA1 cluster formation over the time-course of a BL experimental paradigm.

**Supplemental Video 5:** MAX RNAO treated, OptoA1 cluster formation over the time-course of a BL experimental paradigm.

**Supplemental Video 6:** MED RNAO treated, OptoA1 cluster formation over the time-course of a BL experimental paradigm.

**Supplemental Video 7:** LOW RNAO treated, OptoA1 cluster formation over the time-course of a BL experimental paradigm.
